# Mechanism of the Kinugasa Reaction Revisited

**DOI:** 10.1021/acs.joc.1c01351

**Published:** 2021-07-13

**Authors:** Stefano Santoro, Fahmi Himo

**Affiliations:** † Department of Chemistry, Biology and Biotechnology, University of Perugia, Via Elce di Sotto 8, 06123 Perugia, Italy; ‡ Department of Organic Chemistry, Arrhenius Laboratory, Stockholm University, SE-106 91 Stockholm, Sweden

## Abstract

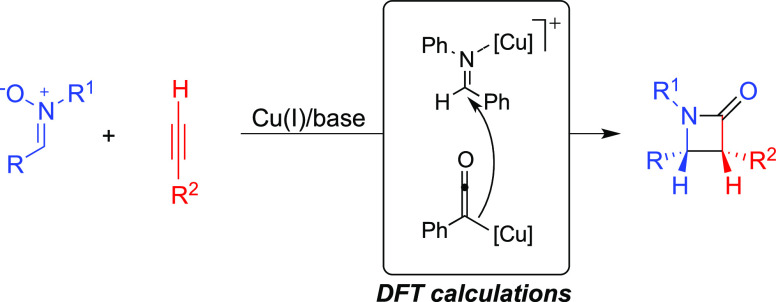

The mechanism of the Kinugasa reaction, that is, the copper-catalyzed formation of β-lactams from nitrones and terminal alkynes, is re-evaluated by means of density functional theory calculations and in light of recent experimental findings. Different possible mechanistic scenarios are investigated using phenanthroline as a ligand and triethylamine as a base. The calculations confirm that after an initial two-step cycloaddition promoted by two copper ions, the resulting five-membered ring intermediate can undergo a fast and irreversible cycloreversion to generate an imine and a dicopper-ketenyl intermediate. From there, the reaction can proceed through a nucleophilic attack of a ketenyl copper intermediate on the imine and an intramolecular cyclization, rather than through the previously suggested (2 + 2) Staudinger synthesis.

## 1 Introduction

**Scheme 1:**
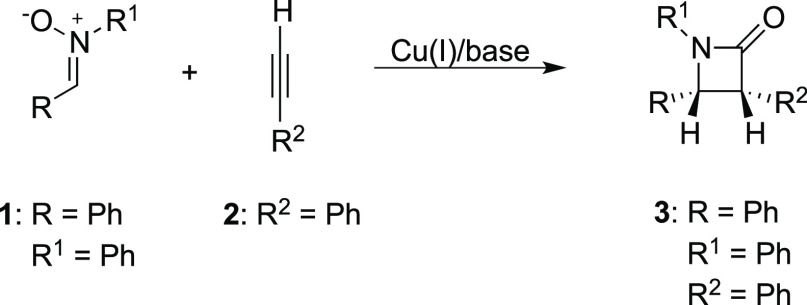
Catalytic Kinugasa Reaction

β-Lactams are among the most important heterocyclic scaffolds in chemistry, representing the core of essential drugs, such as penicillins, cephalosporins, carbapenems, and monobactams.^1^ Moreover, β-lactams can be considered as useful synthons that can be easily converted into other relevant functionalities, such as β-amino acids, amino alcohols, and azetidines.^2^ For these reasons, considerable research efforts have been devoted to the development of effective strategies to access diversely functionalized β-lactams. Among these strategies, the copper-catalyzed reaction between nitrones and alkynes, known as the Kinugasa reaction (Scheme 1), represents certainly an attractive approach.^3^ The reaction was first reported in 1972, making use of preformed copper acetylides,^4^ and was later made catalytic in copper in 1993.^5^ Since then, advances have also been made in the development of enantioselective versions of the reaction,^6^ with reported enantioselectivities of up to 93% ee.^7^

Important limitations of this synthetic methodology are the possible formation of multiple byproducts and the limited availability of highly stereoselective protocols. Despite the considerable research efforts, aimed mainly at expanding the efficiency, scope, and stereoselectivity of the Kinugasa reaction, the mechanism has been relatively poorly understood until recently. A better understanding of the reaction mechanism would certainly help to overcome the shortcomings that still hamper a wider application of the Kinugasa reaction.

**Scheme 2:**
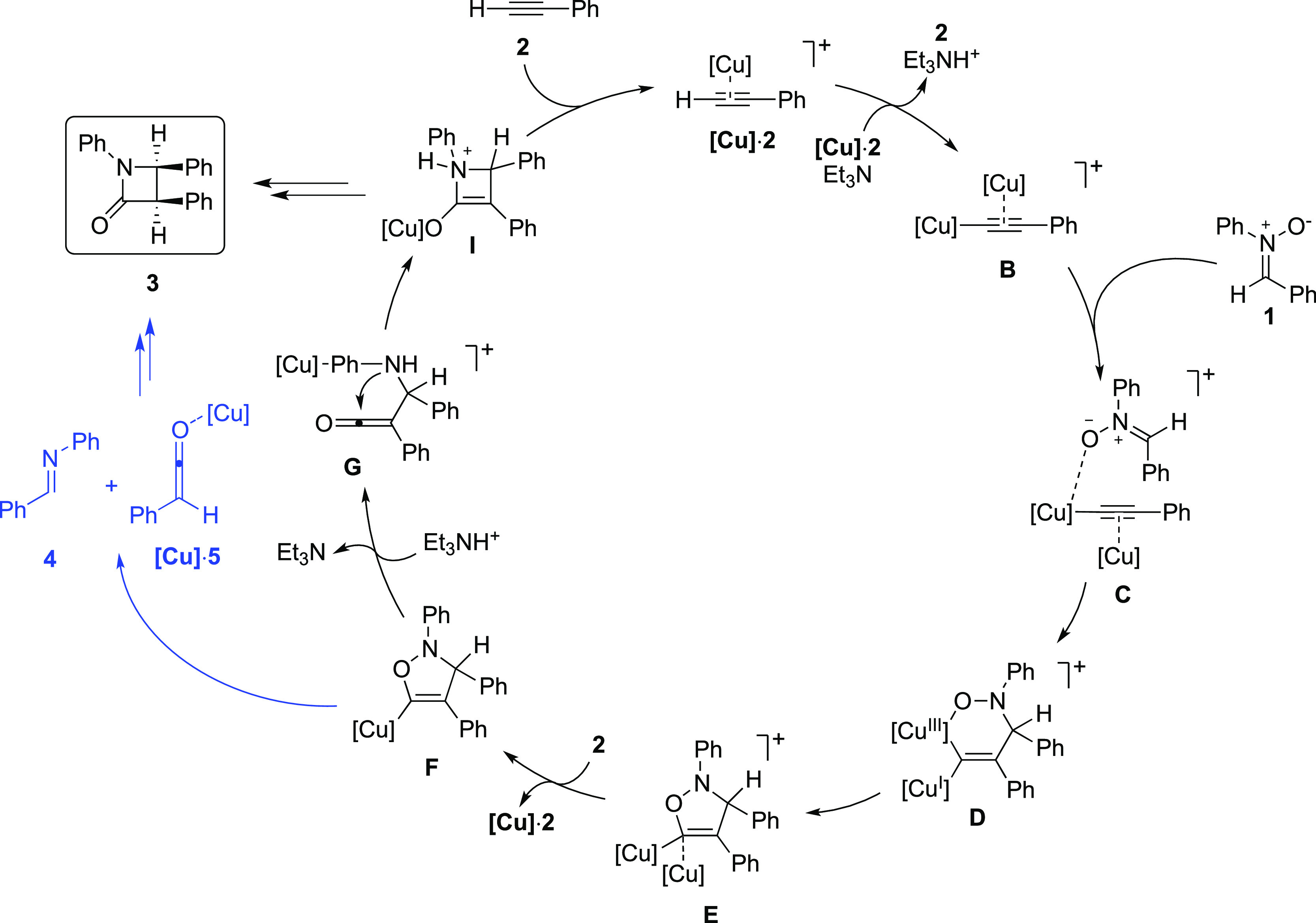
Previously Proposed Reaction Mechanism Based on DFT Calculations (Black),^8^ with the Alternative Pathway Derived from Experiments (Blue)^9^ Note that the naming of the species follows the nomenclature of the previous calculations reported in ref (8).

We reported in 2015 a computational mechanistic investigation based on density functional theory (DFT) calculations, suggesting the involvement of two equivalents of copper in the initial stages of the reaction (Scheme 2).^8^ According to those calculations, the process starts with the deprotonation of the alkyne to generate a dicopper-acetylide (**B**), which then undergoes a stepwise cycloaddition with the nitrone to give a metalated isoxazoline intermediate (**F**). Next, the protonation of the nitrogen of the five-membered ring results in ring opening and the generation of a ketene intermediate (**G**), which can undergo a copper-assisted cyclization to form a four-membered ring intermediate (**I**). A final tautomerization affords the β-lactam product (**3**).^8^

Subsequently, Hein and co-workers reported in 2018 the results of a detailed experimental mechanistic investigation, employing a number of techniques, such as reaction progress kinetic analyses, variable time normalization analyses, and crossover experiments.^9^ The results confirmed that the initial cycloaddition is indeed catalyzed by two copper species. However, the experiments provided strong evidence that copper isoxazolide **F** undergoes a cycloreversion (see the blue path in Scheme 2), affording an imine (**4**) and a ketene intermediate (**[Cu]·5**). It was further suggested that the imine and ketene intermediates could form the final product through a Lewis acid-catalyzed (2 + 2) cycloaddition known as Staudinger synthesis.

The formation of the imine and ketene intermediates helped to rationalize the generation of different byproducts in the reaction.^9^ This proposal has also important implications for understanding the stereoselectivity of the process. Namely, according to this mechanism, the enantioselectivity would be dictated in the final (2 + 2) cycloaddition^9^ rather than in the early cycloaddition between the nitrone and the dicopper-acetylide, as proposed previously.^8^

Considering the limited understanding of the final part of the reaction mechanism, and the disagreement between the proposals put forward on the basis of the theory^8^ and experiments,^9^ we decided to revisit the mechanism of the Kinugasa reaction by performing new DFT calculations to evaluate the various ideas. Since the experiments could confirm the first part of the mechanism, that is, the formation of five-membered ring intermediate **F** through a stepwise cycloaddition catalyzed by two copper species, we focus our attention on the second part, that is, the formation of the β-lactam from the five-membered ring isoxazolide intermediate. With the aim of making the new calculations easily comparable with the previous ones, we consider in the present work the same model reaction between *N*,α-diphenyl nitrone **1** and phenylacetylene **2** with 1,10-phenanthroline as the copper ligand and triethylamine as the base in acetonitrile as the reaction medium.

The new calculations confirm Hein’s finding that the five-membered ring intermediate formed in the initial cycloaddition undergoes a fast and irreversible cycloreversion. However, we found that a mechanism involving a nucleophilic addition of a ketenyl copper intermediate on an imine and the subsequent intramolecular nucleophilic attack of a copper amide on the ketene has lower energy barriers compared to the suggested Staudinger synthesis.

## 2 Results and Discussion

**Figure 1:**
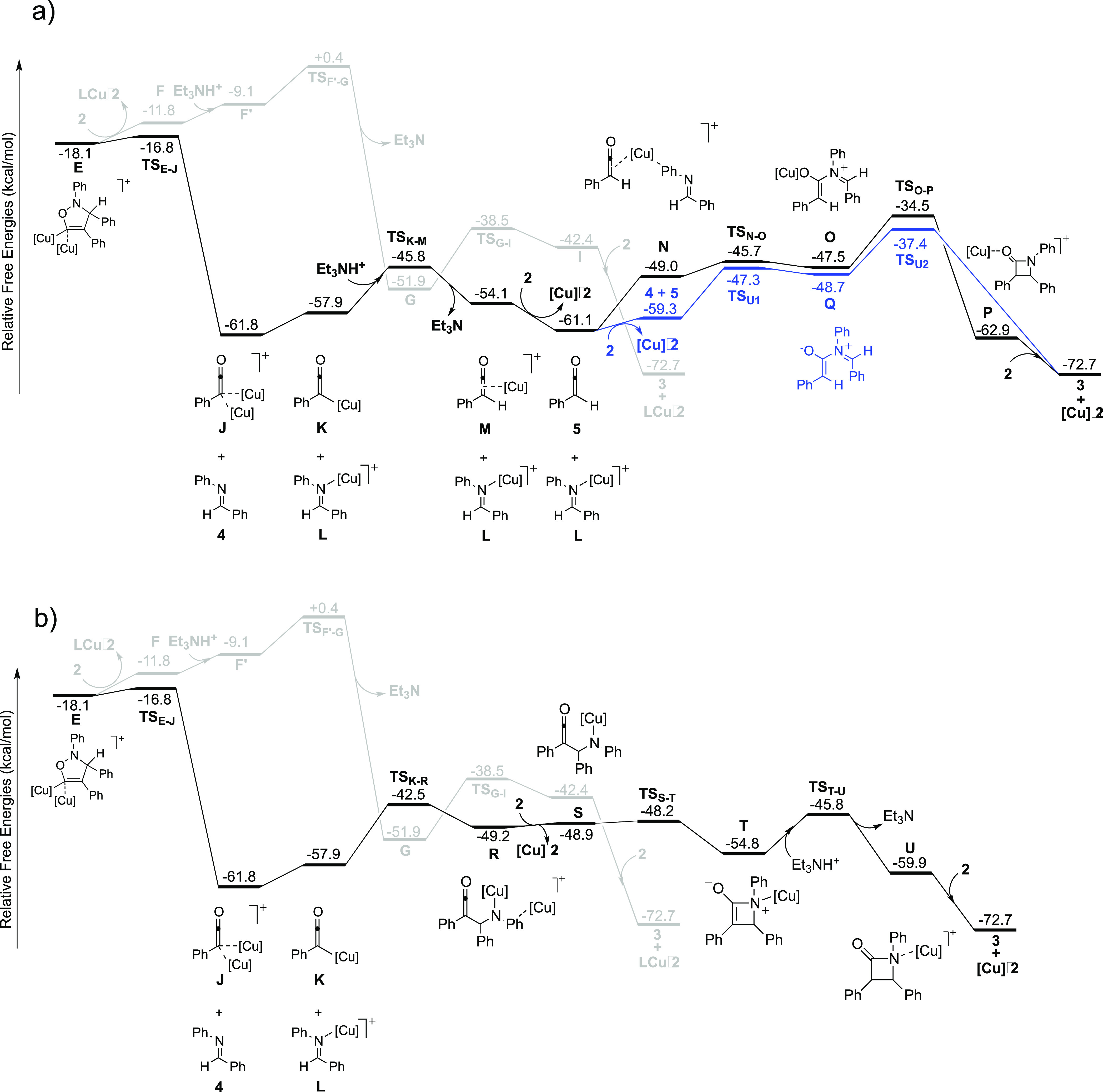
Calculated free-energy profiles for the mechanism of the final part of the reaction. (a) Mechanism via (2 + 2) Staudinger synthesis with and without the involvement of copper (black vs blue, respectively). (b) New mechanism proposed in the present work involving the nucleophilic attack of the ketenyl copper intermediate on the imine and subsequent cyclization. In both cases, the pathway of the previous computational proposal is reported in gray for comparison.

**Figure 2:**
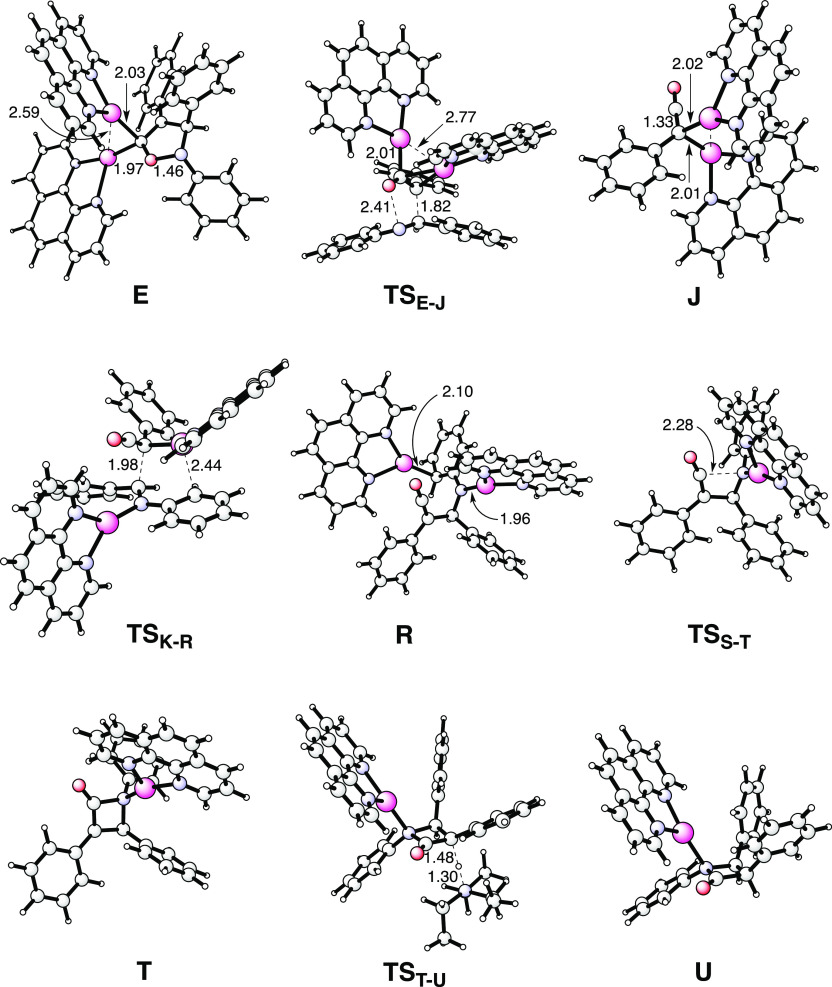
Optimized structures of selected intermediates and transition states for the new mechanistic proposal associated with the lowest energy barriers (cf. free-energy profile in Figure 1b).

We began the investigation by modeling the proposal that the final β-lactam would form through a copper-catalyzed (2 + 2) cycloaddition (see the free-energy profile in Figure 1a). The calculations show that intermediate **E**, the two copper five-membered ring intermediate resulting from the stepwise cycloaddition between nitrone and alkyne, can indeed undergo a highly exergonic cycloreversion through a very low-barrier transition state (**TS**_**E-J**_). This step results in the formation of imine **4** and cationic intermediate **J**, in which a ketenyl anion coordinates to two copper moieties (Figure 2). Next, after a slightly endergonic shift of a phenanthroline–copper complex from **J** to the imine nitrogen, ketenyl copper intermediate **K** can be protonated by Et_3_NH^+^ through **TS**_**K-M**_ to give the copper-coordinated ketene **M**. The energy barrier associated with this step is 16 kcal/mol. Next, after an exergonic dissociation of the copper-ligand moiety (from intermediate **M** to ketene **5**), a stepwise (2 + 2) cycloaddition could occur, initiated by the nucleophilic attack of the imine nitrogen on the copper–ketene complex (**TS**_**N-O**_). The second step of the cycloaddition (**TS**_**O-P**_) is a 4π electron conrotatory electrocyclic reaction, occurring with copper coordinating the oxygen atom. The overall energy barrier for this two-step process, from intermediates **5** + **L** to **TS**_**O-P**_, is 26.6 kcal/mol.

The calculations show thus that this mechanistic proposal is clearly favored over the suggestion based on the previous calculations (gray pathway in Figure 1a) because of the fast and irreversible cycloreversion occurring on intermediate **E** through **TS**_**E-J**_.

From **5** + **L**, there is also the possibility that the (2 + 2) cycloaddition can take place without the participation of copper. Interestingly, this option is associated with a slightly lower barrier (blue pathway in Figure 1a). After a slightly endergonic dissociation of the copper moiety from complex **L**, imine **4** can attack as a nucleophile ketene **5** through **TS**_**U1**_, generating the zwitterionic intermediate **Q**. Next, a 4π electron conrotatory electrocyclic reaction (**TS**_**U2**_) affords the final product **3**. The overall energy barrier for this alternative is 23.7 kcal/mol as compared to the 26.6 kcal/mol obtained for the copper-mediated Staudinger synthesis.

This finding suggests that if this mechanism would be operative, it would not be possible to perform enantioselective Kinugasa reactions using chiral copper ligands since the background non-enantioselective process would be faster. The energy difference with and without copper is, however, rather small and will depend strongly on the nature of the copper ligand. A bulkier ligand would likely result in a further increase of the barrier of the copper-mediated (2 + 2) cycloaddition relative to the one not involving copper.

As discussed above, the calculations demonstrate that the pathway with the Staudinger synthesis is energetically feasible. However, in the course of our investigations, we found an alternative mechanism that is associated with even lower barriers (Figure 1b). Namely, after the irreversible cycloreversion, the ketenyl copper intermediate **K** can perform a nucleophilic attack on the Cu-coordinated imine **L** through **TS**_**K-R**_, resulting in the formation of ketene intermediate **R**. The overall energy barrier for this step, that is, from **J** + **4** to **TS**_**K-R**_, is 19.3 kcal/mol.^10^ At transition state **TS**_**K-R**_, there is a stabilizing interaction between the copper of the nucleophile and the aromatic ring bound to the imine nitrogen (see optimized structures in Figure 2).

From intermediate **R**, a nearly thermoneutral dissociation of the phenyl-coordinated copper moiety generates the neutral ketene intermediate **S**, which can then undergo a very fast intramolecular nucleophilic attack of the nitrogen on the ketene carbonyl through **TS**_**S-T**_ (calculated barrier of 0.7 kcal/mol), resulting in the formation of the four-membered ring intermediate **T**.^11^

To close the catalytic cycle, protonation of the α-position of the lactam enolate **T** is required. This can occur through **TS**_**T-U**_ with the Et_3_NH^+^ species generated from the initial deprotonation of the alkyne. In **TS**_**T-U**_, the protonation occurs on the least hindered face of the lactam enolate, generating thus the kinetically favored *cis*-lactam **U**. This step has a low energy barrier of 9.0 kcal/mol. We also optimized the related protonation TS leading to the formation of the diastereoisomeric *trans*-lactam (see Supporting Information), which was found to be 4.9 kcal/mol higher in energy than **TS**_**T-U**_. This energy difference, which stems from steric interactions between the bulky Et_3_NH^+^ and the phenyl ring in the β-position at the transition state leading to the trans-product, is in agreement with the observed typical preferential formation of the *cis*-lactam in the Kinugasa reaction. Finally, from intermediate **U** an exergonic ligand exchange gives product **3** and the copper-alkyne complex **[Cu]·2**, which can start a new catalytic cycle.

**Scheme 3:**
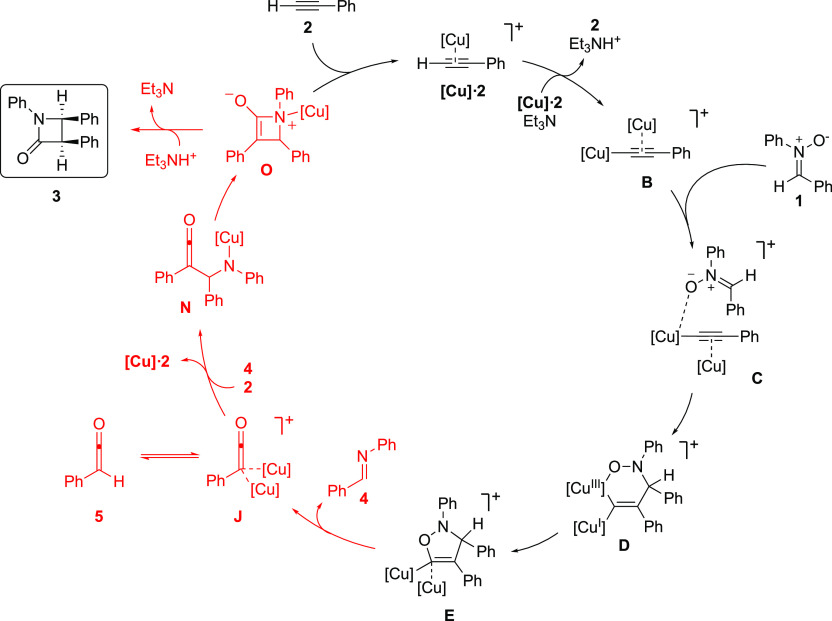
Revised Mechanism for the Catalytic Kinugasa Reaction on the Basis of Present Calculations The new steps are indicated in red.

The new mechanism obtained on the basis of the calculations is summarized in Scheme 3, and the full free-energy profile, including also the initial cycloaddition part, is provided in the Supporting Information. According to the calculations, the highest energy barrier of the cycle is associated with the nucleophilic attack of the ketenyl copper intermediate on the copper-coordinated imine (from **J** and **4** to **TS**_**K-R**_, 19.3 kcal/mol). This represents a significant lowering compared to the (2 + 2) Staudinger alternative, by at least 5 kcal/mol (Figure 1b vs 1a).

An important feature of the mechanism is the equilibrium between dicopper-ketenyl **J** and ketene **5**, which is in line with the evidence of the intermediacy of the ketene observed in the experiments.^9^ Experimentally, it was concluded on the basis of the kinetics and isotope effects that the initial cycloaddition is the turnover-determining step of the reaction.^9^ In the present calculations, we find the barrier for the initial cycloaddition to be 14.8 kcal/mol, which is 4.5 kcal/mol lower than the highest barrier (**TS**_**K-R**_) of the new mechanism. It should, however, be remembered that we here use different ligand and base compared to the experiments (phenanthroline vs tris((1-cyclopentyl-1*H*-1,2,3-triazol-4-yl)methyl)amine and triethylamine vs diisopropylamine), and previous calculations have shown that the energies of the Kinugasa reaction can be quite sensitive to the reaction conditions.^8^ Therefore, we do not believe that the experiments necessarily contradict the calculations.

## 3 Conclusions

In the present work, the mechanism of the catalytic Kinugasa reaction has been revisited by means of DFT calculations, performed on a model reaction involving phenanthroline as a copper ligand and triethylamine as a base. Previous experimental work has demonstrated that the five-membered ring intermediate, formed in the initial cycloaddition between the nitrone and dicopper-acetylide, undergoes cycloreversion to generate ketene and imine intermediates. This is then suggested to be followed by a copper-catalyzed (2 + 2) Staudinger synthesis to generate the final β-lactam product.^9^

The present calculations show that the five-membered ring intermediate can indeed dissociate in a fast and irreversible cycloreversion step, generating an imine and a dicopper-ketenyl intermediate. Furthermore, the copper-catalyzed (2 + 2) Staudinger synthesis was found to be associated with feasible barriers. However, the Staudinger synthesis without the involvement of copper was found to have slightly lower barriers.

More importantly, we found an alternative pathway that has significantly lower barriers. Namely, the calculations show that the final part of the catalytic cycle rather proceeds through nucleophilic addition of a ketenyl copper intermediate on the imine, followed by a cyclization by an intramolecular nucleophilic attack of a copper amide on the ketene carbonyl.

The new mechanism is connected to the Staudinger pathway by a protonation event, which means that the relative energies of the two pathways will depend on the strength of the base used in the experiments (or more accurately the strength of its conjugate acid). Therefore, we emphasize that the energetics of the different mechanistic possibilities can be sensitive to the nature of the ligand and of the base, which should be considered before extending the conclusions of this study to different catalytic systems.

## 4 Computational Methods

All calculations were carried out using DFT with the B3LYP functional,^12^ as implemented in the Gaussian09 program package.^13^ For geometry optimizations, the 6-31G(d,p) basis set was used for the C, N, O, and H elements and the LANL2DZ^14^ pseudopotential was used for Cu. Based on these optimized geometries, single-point calculations were carried out with the 6-311+G(2d,2p) basis set for all elements. The stationary points were confirmed as minima (no imaginary frequencies) or transition states (only one imaginary frequency) by analytical frequency calculations at the same theory level as the geometry optimizations. The reported energies are Gibbs free energies, which include zero-point vibrational corrections, thermal and entropy corrections at 298 K, and solvation energies. The latter are calculated as single-point corrections on the optimized structures with the same basis set combination used for the geometry optimizations, using the SMD continuum solvation model^15^ with the parameters for acetonitrile. All energies were also corrected with single-point dispersion effects using the DFT-D3 method of Grimme^16^ with Becke and Johnson damping.^17^

## Supporting Information Available

Revised energies of the previous mechanistic proposal; additional results on alternative mechanistic possibilities; optimized structures for the Staudinger synthesis pathway; free-energy profile of the complete catalytic cycle; energies and energy corrections of stationary points; and Cartesian coordinates of all stationary points (PDF)
