# Supplementary material for: Mechanism of the Kinugasa Reaction Revisited
Source: J Org Chem. 2021 Jul 13;86(15):10665–71. doi: 10.1021/acs.joc.1c01351 (PMC8389905; doi:10.1021/acs.joc.1c01351)
Supplement: Supplementary file 1 — jo1c01351_si_001.pdf [file jo1c01351_si_001.pdf]

SUPPORTING INFORMATION

Mechanism of the Kinugasa Reaction Revisited

Stefano Santoro,[a] Fahmi Himo*[b]*

[a] Department of Chemistry, Biology and Biotechnology, University of Perugia, Via Elce di Sotto 8, 06123, Perugia, Italy

[b] Department of Organic Chemistry, Arrhenius Laboratory, Stockholm University, SE-106 91 Stockholm, Sweden

Corresponding authors: stefano.santoro@unipg.it ; fahmi.himo@su.se.

Contents

1. Revised energies of previous mechanistic proposal S2
2. Additional results on alternative mechanistic possibilities S3
3. Optimized structures for the Staudinger synthesis pathway S6
4. Free energy profile of the complete catalytic cycle S7
5. Energies and energy corrections of stationary points S8
6. References S9
7. Cartesian coordinates S10

1. Revised energies of previous mechanistic proposal

There are some small differences in the computational details used in the current work compared to our previous computational investigation[1] (SMD solvation method instead of CPCM and D3 dispersion correction instead of D2). We decided therefore to reassess our previously proposed reaction mechanism with the updated computational protocol in order to allow a direct comparison with the newly explored mechanistic pathways. The results are given in Figure S1. The effects on the obtained energy barriers are marginal. However, while in our previous work we only considered the thermodynamics of the protonation of copper isoxazolide **F** by Et_3NH^+, we were now able to locate a transition state for this step (**TS**$_{\mathbf{F'-G}}$, see blue addition to the free energy profile in Figure S1). This is important because it shows that accessing the key ketene intermediate **G** would be energetically feasible, but through a higher energy barrier than reported previously (18.5 kcal/mol, from **E** to **TS**$_{\mathbf{F'-G}}$).[1] It is, however, important to remember that this energy barrier could change significantly depending on the nature of the base used in the reaction.

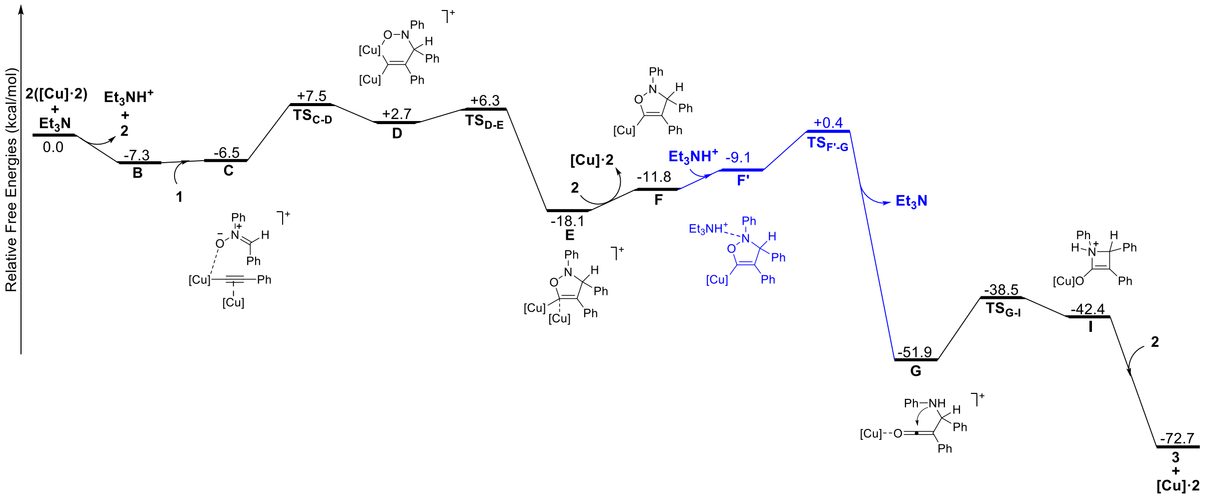

Figure S1. *Calculated free energy profile for the previous mechanistic proposal[1] with the new computational protocol. Blue color indicates a newly located part.*

2. Additional results on alternative mechanistic possibilities

The Staudinger synthesis is in general catalyzed by a Lewis base, through an initial nucleophilic attack on the ketene carbonyl, a subsequent addition of the zwitterionic enolate to the imine and a final cyclization.[2] We decided therefore to also model this mechanistic possibility (see free energy profile in Figure S2 and optimized structures in Figure S3). We use Et_3N as a Lewis base catalyst in the model, since it is known from experiments to be able to promote this reactivity.[3]

We found that Et_3N can attack the copper-coordinated carbonyl of ketene **5** through **TS$_{5\text{-}V}$**, with an activation energy of 23.3 kcal/mol, to give intermediate **V**, in which copper coordinates both the anionic oxygen and the imine nitrogen. This step in endergonic by 17.5 kcal/mol. Next, a nucleophilic attack of the enolate carbon on the copper-coordinated imine can occur through **TS$_{V\text{-}W}$**. The energy barrier for this C–C bond formation is, however, 37.7 kcal/mol (from **5** + **L** to **TS$_{V\text{-}W}$**), which is too high for this pathway to be viable under the typical Kinugasa reaction conditions.

For completeness we also located the TS for the final cyclization through a nucleophilic attack of the nitrogen on the acyl ammonium group (**TS$_{W\text{-}U}$**), which would produce the copper-product complex **U** and regenerate the Lewis base catalyst. This step has an activation energy of 11.8 kcal/mol and is exergonic by 22 kcal/mol.

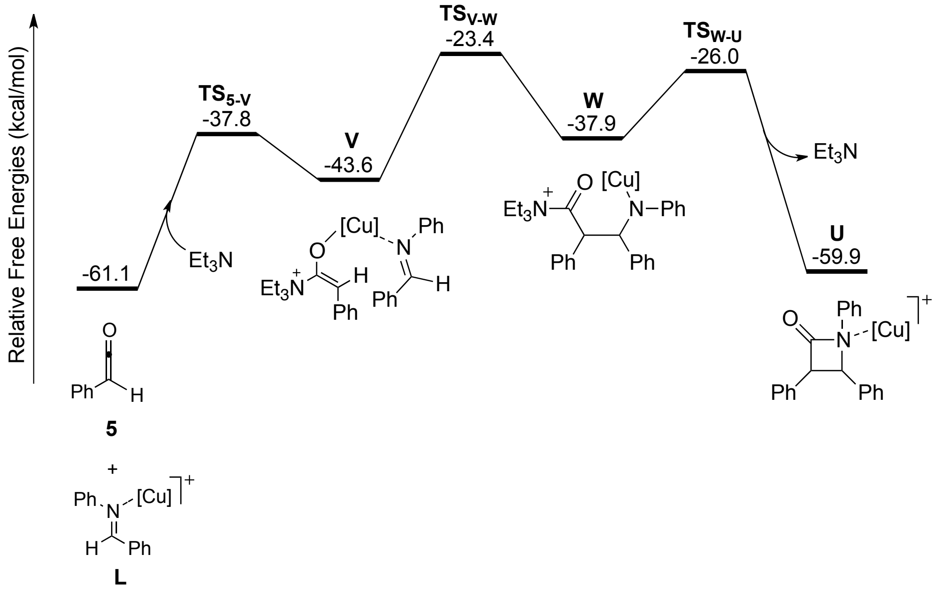

Figure S2. *Free energy profile for an alternative mechanism involving a Lewis base-catalyzed Staudinger synthesis of the β-lactam.*

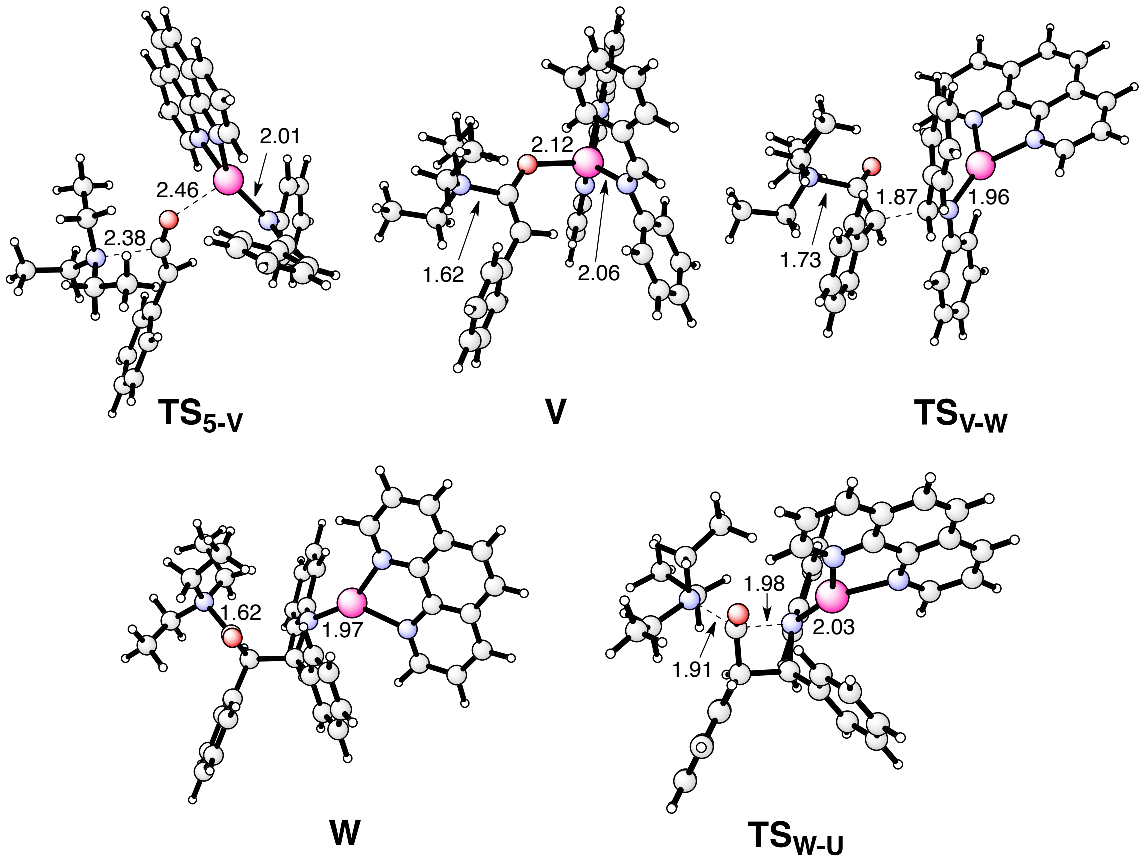

Figure S3. *Optimized structures of selected intermediates and transition states for the alternative mechanisms involving a Lewis base-catalyzed Staudinger synthesis of the β-lactam (cf. free energy profile in Figure S2).*

As mentioned in the paper, we also located a TS for the nucleophilic addition of ketenyl copper intermediate **K** on the free imine **4**, as an alternative to $\mathbf{TS_{K\text{-}R}}$. The energy of this TS ($\mathbf{TS_{K\text{-}S}}$, see Figure S4) is, however, 10.6 kcal/mol higher than the energy of $\mathbf{TS_{K\text{-}R}}$.

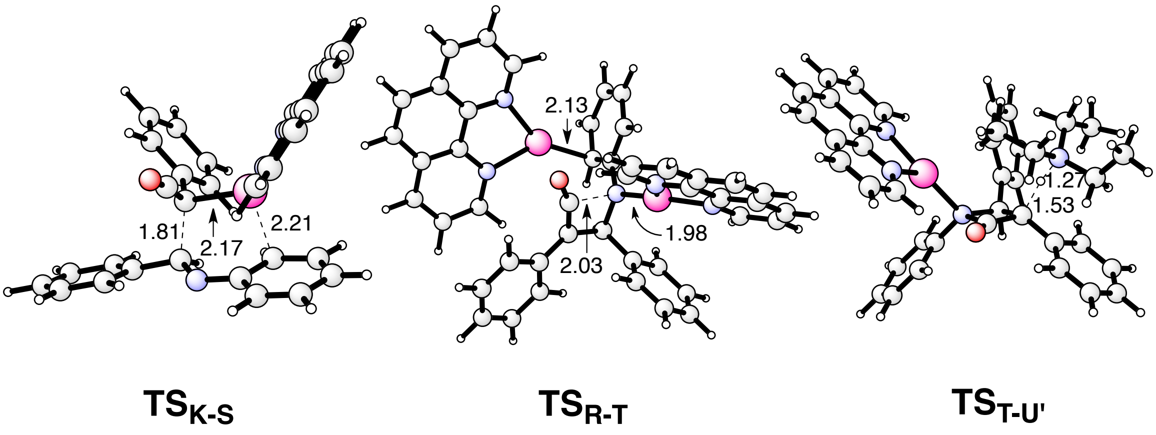

Figure S4. *Additional optimized structures for alternative mechanistic possibilities.*

In addition, we have also optimized a TS for the final cyclization occurring on intermediate **R**, without the prior dissociation of one of the copper moieties, as an alternative to **TS$_{\mathbf{S\text{-}T}}$**. This transition state (**TS$_{\mathbf{R\text{-}T}}$**, see Figure S4) is 1.0 kcal/mol higher in energy compared to **TS$_{\mathbf{S\text{-}T}}$**.

Finally, as mentioned in the paper, we also located a TS for the protonation of lactam enolate **T** (**TS$_{\mathbf{T\text{-}U'}}$**, see Figure S4), similar to **TS$_{\mathbf{T\text{-}U}}$** but leading to the formation of the diastereoisomeric *trans*-lactam. This TS is however 4.9 kcal/mol higher in energy than **TS$_{\mathbf{T\text{-}U}}$**. The calculated energy difference derives from steric interactions in **TS$_{\mathbf{T\text{-}U'}}$** between the bulky Et_3NH^+ and the phenyl ring in the β-position.

3. Optimized structures for the Staudinger synthesis pathway

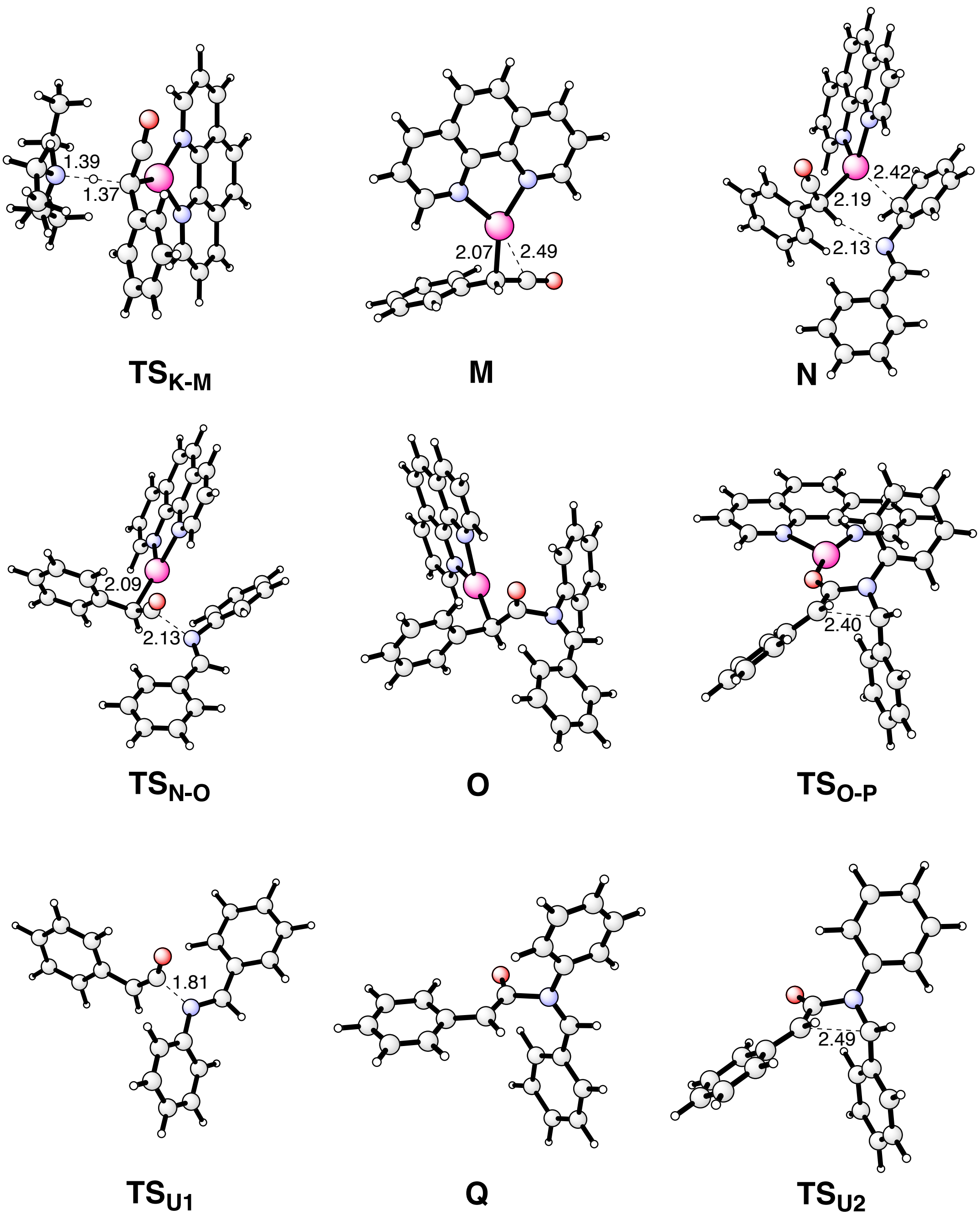

Figure S5. *Optimized structures of selected intermediates and transition states for the alternative mechanisms involving Staudinger (2 + 2) cycloadditions (cf. free energy profiles in Figure 1a in the paper).*

4. Free energy profile of the complete catalytic cycle

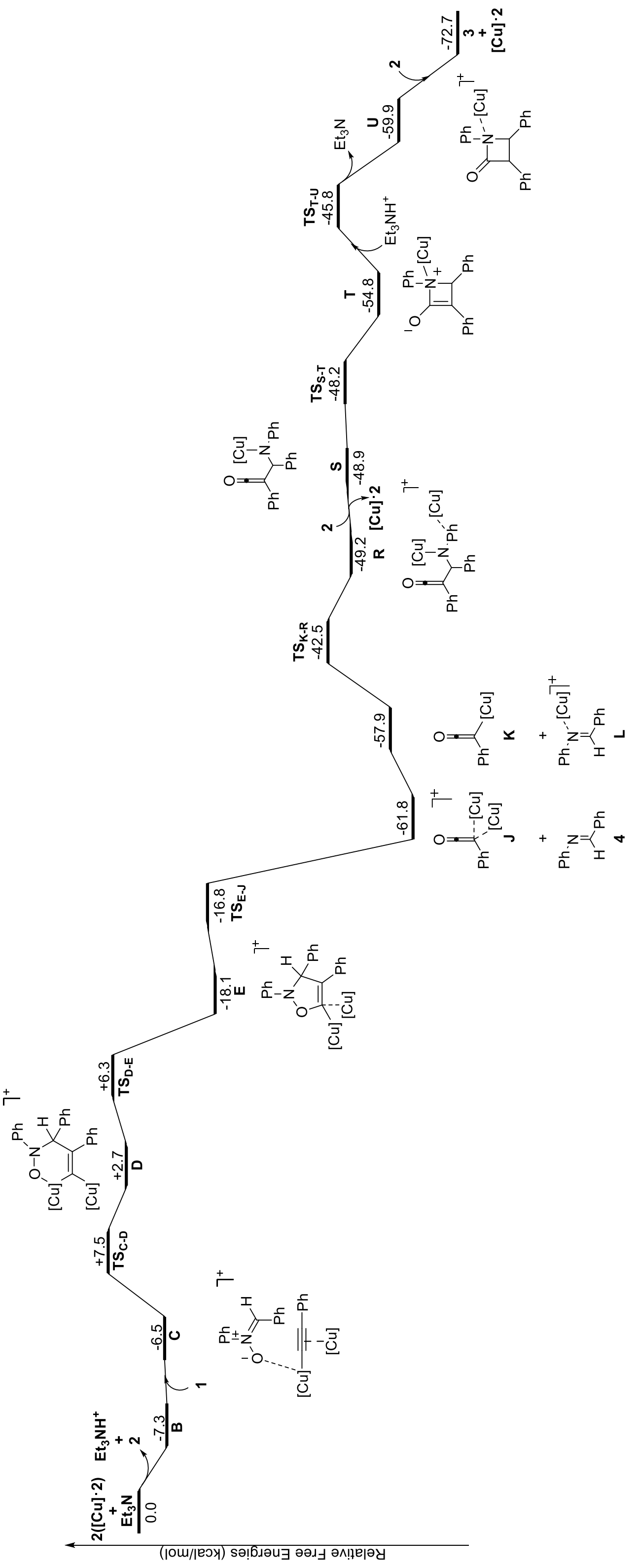

Figure S6. *Calculated free energy profile of the complete catalytic cycle for the reaction involving a nucleophilic attack of the ketenyl copper intermediate on the imine and subsequent cyclization.*

5. Energies and energy corrections of stationary points

Stationary point	B3LYP/ 6-311+G(2d,2p) (a.u.)	Thermal correction to Gibbs free energy (a.u.)	Solvation correction (kcal/mol)	D3 correction (kcal/mol)
**1**	-632.105566	0.169442	-11.98	-33.30
**2**	-308.488312	0.079096	-6.24	-15.38
**3**	-940.719988	0.274306	-16.74	-59.89
**4**	-556.913964	0.165077	-10.51	-31.24
**5**	-383.775174	0.082008	-6.76	-17.04
**[Cu]·2**	-2520.650868	0.234289	-53.16	-59.02
**Et_3N**	-292.513919	0.171981	-4.06	-18.63
**Et_3NH^+**	-292.901278	0.188189	-59.85	-20.19
**B**	-4732.418376	0.376352	-58.37	-101.04
**C**	-5364.529657	0.562888	-63.32	-147.79
**TS_{C-D}**	-5364.501390	0.571905	-64.88	-155.60
**D**	-5364.507310	0.574964	-66.76	-156.66
**TS_{D-E}**	-5364.499176	0.571419	-66.86	-155.87
**E**	-5364.542904	0.574587	-66.02	-155.64
**F**	-3152.360197	0.415815	-33.00	-102.25
**F'**	-3445.306115	0.627479	-60.90	-138.41
**$TS_{F'-G}$**	-3445.291747	0.623716	-56.20	-140.23
**G**	-3152.845688	0.424024	-59.68	-106.45
**TS_{G-I}**	-3152.830274	0.428670	-58.85	-106.38
**I**	-3152.845024	0.429407	-58.51	-101.89
**TS_{E-J}**	-5364.522771	0.569557	-71.86	-158.00
**J**	-4807.693516	0.382041	-59.30	-106.53
**K**	-2595.506434	0.224830	-24.58	-59.23
**L**	-2769.081108	0.320520	-54.79	-76.53
**TS_{K-M}**	-2888.431713	0.428762	-54.29	-92.34
**M**	-2595.920372	0.236553	-56.91	-60.95
**N**	-3152.843282	0.421093	-55.74	-107.11
**TS_{N-O}**	-3152.837905	0.423679	-58.14	-106.46
**O**	-3152.844977	0.428445	-58.47	-106.46
**TS_{O-P}**	-3152.833296	0.427889	-55.15	-103.79
**P**	-3152.878154	0.429595	-56.34	-103.88
**TS_{U1}**	-940.674777	0.270600	-20.46	-56.77
**Q**	-940.673907	0.271811	-22.65	-57.30
**TS_{U2}**	-940.663266	0.272659	-17.71	-58.16
**TS_{K-R}**	-5364.576735	0.570540	-62.19	-160.14
**R**	-5364.587835	0.569238	-63.49	-157.77
**S**	-3152.413576	0.414823	-29.87	-108.36
**TS_{S-T}**	-3152.411168	0.414442	-30.41	-108.39
**T**	-3152.419978	0.417849	-34.16	-107.84
**TS_{T-U}**	-3445.356227	0.624255	-58.70	-143.78
**U**	-3152.857279	0.431415	-60.27	-111.20
**TS_{5-V}**	-3445.356949	0.618657	-54.91	-135.62
**V**	-3445.364220	0.622519	-55.79	-138.43
**TS_{V-W}**	-3445.321884	0.627712	-60.98	-142.82
**W**	-3445.338453	0.629087	-62.67	-146.09
**TS_{W-U}**	-3445.328331	0.631835	-56.66	-148.31
**TS_{K-S}**	-3152.377126	0.413550	-31.45	-111.80
**TS_{R-T}**	-5364.582410	0.571692	-64.42	-159.73
**$TS_{T-U'}$**	-3445.344963	0.624244	-59.81	-144.80

6. References

[1] Santoro, S.; Liao, R.-Z.; Marcelli, T.; Hammar, P.; Himo, F. Theoretical Study of Mechanism and Stereoselectivity of Catalytic Kinugasa Reaction. *J. Org. Chem.* **2015**, *80*, 2649−2660.

[2] (a) Hodous, B. L.; Fu, G. C. Enantioselective Staudinger Synthesis of β-Lactams Catalyzed by a Planar-Chiral Nucleophile. *J. Am. Chem. Soc.* **2002**, *124*, 1578−1579. (b) Taggi, A. E.; Hafez, A. M.; Wack, H.; Young, B.; Drury, W. J.; Lectka, T. Catalytic, Asymmetric Synthesis of β-Lactams. *J. Am. Chem. Soc.* **2000**, *122*, 7831−7832.

[3] (a) Zarei, M.; Jarrahpour, A. A Mild and Efficient Route to 2-Azetidinones Using the Cyanuric Chloride-DMF Complex. *Synlett* **2011**, 2572−2576. (b) Van Brabandt, W.; Vanwalleghem, M.; D'hoogh, M.; De Kimpe, N. Asymmetric Synthesis of 1-(2- and 3-Haloalkyl)azetidin-2-ones as Precursors for Novel Piperazine, Morpholine, and 1,4-Diazepane Annulated Beta-Lactams. *J. Org. Chem.* **2006**, *71*, 7083−7086.

7. Cartesian coordinates

1

C	0.45158600	0.41909600	0.11872400
H	0.07486600	1.40248800	0.36518900
C	1.88480200	0.22836800	0.07085500
C	2.68823200	1.36109100	0.33585800
C	2.52496700	-0.99644000	-0.22278500
C	4.07484300	1.27892000	0.30824700
H	2.20947800	2.31050400	0.56385000
C	3.91640200	-1.06562800	-0.25021000
H	1.91415400	-1.86543800	-0.42197300
C	4.69717100	0.06161900	0.01373100
H	4.67220500	2.16224700	0.51525900
H	4.39538900	-2.01366300	-0.47870600
H	5.78111600	-0.00549000	-0.00823900
C	-1.88530600	-0.12256000	-0.04291900
C	-2.78420200	-1.08303100	0.42228300
C	-2.33012300	1.12654900	-0.48114400
C	-4.14003800	-0.77082200	0.48853600
H	-2.39946700	-2.05261200	0.71345500
C	-3.69169200	1.42548600	-0.41520900
H	-1.63409100	1.84537600	-0.89948400
C	-4.59753700	0.48315500	0.07454700
H	-4.84200300	-1.51104500	0.86068400
H	-4.04335200	2.39194800	-0.76360700
H	-5.65629300	0.71980800	0.12092400
N	-0.47659900	-0.50179800	-0.09029900
O	-0.24880500	-1.74329500	-0.30729400

2

C	3.23449300	0.00000100	0.00047000
H	4.29967000	-0.00000200	0.00062400
C	2.02421300	0.00000000	0.00027500
C	0.59430900	-0.00000400	0.00006200
C	-0.11993300	-1.21339400	-0.00001700
C	-0.11992700	1.21338800	-0.00002100
C	-1.51262800	-1.20868300	-0.00020700
H	0.42840500	-2.14987700	0.00006700
C	-1.51262400	1.20868700	-0.00021000
H	0.42841400	2.14986900	0.00006100
C	-2.21310800	0.00000500	-0.00030900
H	-2.05303400	-2.15087000	-0.00027400
H	-2.05301900	2.15088000	-0.00028000
H	-3.29920500	0.00000100	-0.00045500

3

C	-0.38540200	1.35513800	1.01242600
C	-0.27585800	1.33904900	-0.38438600
C	-0.60819200	2.49478100	-1.10226500
C	-1.05133200	3.64227500	-0.44256300
C	-1.15886300	3.64814800	0.94847700
C	-0.82235900	2.50215600	1.67312800
C	0.16680100	0.10534600	-1.13225500
N	1.17514400	-0.74477600	-0.46627200
C	2.52503900	-0.50499200	-0.15799800
C	3.30395800	-1.51911000	0.42545900
C	4.64375000	-1.27038100	0.71344600
C	5.22045200	-0.02888500	0.43303600
C	4.43936500	0.97340600	-0.14335700
C	3.09620400	0.74429300	-0.44008000
O	0.60137200	-3.00174300	-0.01274900
C	0.39146000	-1.88426300	-0.43734100
C	-0.77608100	-1.17711600	-1.14850500
C	-2.13370000	-1.14603300	-0.48961000
C	-3.15669600	-0.36185400	-1.04337900
C	-4.42186000	-0.32656800	-0.46050600
C	-4.68738900	-1.08370400	0.68335900
C	-3.68000600	-1.87497500	1.23435900
C	-2.41002700	-1.90736800	0.65299200
H	-1.63646100	-2.54204400	1.07260500
H	-3.87975000	-2.47399800	2.11838900
H	-5.67421400	-1.05961800	1.13674100
H	-5.20113800	0.28989000	-0.89973400
H	-2.95803900	0.23028700	-1.93305900
H	0.48138200	0.38500800	-2.14509400
H	2.85091200	-2.48001800	0.63673400
H	5.24220100	-2.05817400	1.16227100
H	6.26550600	0.15482100	0.66307400
H	4.87325200	1.94451500	-0.36400600
H	2.49227400	1.53214600	-0.87697400
H	-0.51702900	2.49674400	-2.18633700
H	-1.30410900	4.53071800	-1.01418400
H	-1.49777000	4.54090800	1.46600300
H	-0.89948400	2.50172300	2.75655200
H	-0.12175900	0.46964300	1.58204100
H	-0.86833300	-1.55122300	-2.17575000

4

C	-2.37723100	-1.05095900	0.33917300
C	-1.84995600	0.18241500	-0.08287800
C	-2.72956300	1.22051100	-0.42725100
C	-4.10962400	1.03459100	-0.35237800
C	-4.62372300	-0.19357900	0.06687400
C	-3.75350900	-1.23490800	0.41142100
C	-0.40153500	0.40618700	-0.17079300
N	0.45639100	-0.50161000	0.11396100
C	1.83171000	-0.21409000	0.06778300
C	2.69460700	-1.22833100	-0.38013900
C	4.06760500	-1.00845900	-0.44757600
C	4.60729000	0.21465700	-0.03835800
C	3.75943700	1.21583200	0.44000100
C	2.38154900	1.00880300	0.49297300
H	-0.09575300	1.40709900	-0.51174900
H	2.26161100	-2.17657900	-0.68228600
H	4.72117700	-1.79705300	-0.80999300
H	5.68011800	0.37932200	-0.07638600
H	4.17265700	2.16075600	0.78225200
H	1.73046900	1.77816200	0.89735400
H	-2.32590900	2.17586500	-0.75454300
H	-4.78157000	1.84462400	-0.62079000
H	-5.69832600	-0.34146800	0.12555000
H	-4.15481900	-2.19023500	0.73754900
H	-1.68673100	-1.84524800	0.60219800

5

C	-0.97256100	-1.29742700	-0.00006600
C	0.08706300	-0.37434900	0.00015800
C	-0.22236900	0.99842500	0.00025200
C	-1.54660400	1.42735500	0.00008300
C	-2.59326200	0.50119400	-0.00014600
C	-2.29690500	-0.86244400	-0.00020900
C	1.46933600	-0.87328600	0.00033400
H	1.65562200	-1.94322000	0.00066300
H	0.57869500	1.73378500	0.00047500
H	-1.76211900	2.49219200	0.00015500
H	-3.62500400	0.83930900	-0.00025800
H	-3.09942000	-1.59482100	-0.00038300
H	-0.75325100	-2.36184200	-0.00013200
C	2.55315300	-0.11458200	0.00006500
O	3.51729600	0.55066100	-0.00041900

[Cu]·2

Cu	-0.25204300	-1.18497900	-0.04678700
C	3.98007100	-2.41874800	0.01976900
C	4.60069000	-1.18534900	0.04618400
C	3.81781200	-0.00793700	0.04018100
C	2.41068900	-0.16390600	0.00688400
C	2.57520200	-2.47509800	-0.01226600
C	4.38040600	1.31291400	0.06586000
C	1.56393900	1.00558600	-0.00163700
C	2.15084300	2.29401700	0.02283500
C	3.58095200	2.41685100	0.05747500
C	1.27631300	3.40501000	0.01088900
H	1.68707000	4.41029900	0.02911300
C	-0.08888600	3.19977000	-0.02509700
C	-0.58240600	1.88302500	-0.04739700
H	5.46088200	1.41546700	0.09176600
H	4.55325900	-3.33895600	0.02342300
H	5.68378600	-1.10872700	0.07134800
H	2.06045000	-3.43066100	-0.03296400
H	4.01500300	3.41169800	0.07662500
H	-0.78367300	4.03191100	-0.03633100
H	-1.65014000	1.68859000	-0.07880800
N	1.81171000	-1.38539000	-0.01840700
N	0.21630000	0.81786800	-0.03479100
C	-1.50659400	-2.74691300	-0.05962500
H	-1.22185900	-3.78148900	-0.08968200
C	-2.30668400	-1.79905300	-0.04380000
C	-3.35022300	-0.81571500	-0.00818500
C	-3.81205500	-0.22665900	-1.20296100
C	-3.93345300	-0.45360400	1.22297000
C	-4.84623600	0.70445000	-1.15995700
H	-3.36726400	-0.51450200	-2.15028800
C	-4.96707100	0.47826100	1.25075200
H	-3.57982600	-0.91391700	2.13982900
C	-5.42376400	1.05788400	0.06339600
H	-5.20762200	1.14909000	-2.08178300
H	-5.42099000	0.74873300	2.19876100
H	-6.23315600	1.78078800	0.09055900

Et_3N

N	0.00063600	0.00009400	0.02057000
C	1.35193600	-0.38512300	0.44637900
H	1.42339100	-1.47597900	0.39644900
C	-0.34113800	1.36327000	0.44545300
H	0.56719900	1.97125400	0.39202800
C	-1.00845500	-0.97724800	0.44747300
H	-1.98881100	-0.49338900	0.39939200
H	-0.86439100	-1.26844200	1.50657300
C	-1.04716600	-2.22388600	-0.43889300
H	-0.10323600	-2.77789800	-0.41686900
H	-1.83524800	-2.90670700	-0.10321600
H	-1.24624400	-1.94398500	-1.47750000
C	-1.40589700	2.01645600	-0.43822600
H	-1.60278700	3.04104400	-0.10445400
H	-1.06846300	2.04636000	-1.47838000
H	-2.35690800	1.47494500	-0.41048100
H	-0.66114400	1.38635500	1.50581400
H	1.53220200	-0.11648500	1.50590500
C	2.45047900	0.20656900	-0.43916800
H	2.45794300	1.30096000	-0.41498000
H	3.43590900	-0.13491200	-0.10408500
H	2.30758300	-0.10400400	-1.47829600

Et_3NH^+

N	0.00000000	0.00000000	0.01283600
C	-1.40048600	-0.40715900	0.45603200
H	-2.01655100	0.49172700	0.40991800
C	1.05285300	-1.00927700	0.45603200
H	0.58242700	-1.99224800	0.40991800
C	0.34763300	1.41643600	0.45603200
H	1.43412400	1.50052100	0.40991800
H	0.04479700	1.48562700	1.50406500
C	-0.30978400	2.48134400	-0.41108900
H	-1.40178000	2.45165400	-0.37376800
H	0.00000000	3.46436700	-0.04674000
H	0.01109400	2.40539300	-1.45537800
C	2.30379900	-0.97239100	-0.41108900
H	3.00023000	-1.73218400	-0.04674000
H	2.07758400	-1.21230400	-1.45537800
H	2.82408500	-0.01185000	-0.37376800
H	1.26419200	-0.78160900	1.50406500
H	-1.30899000	-0.70401800	1.50406500
C	-1.99401500	-1.50895300	-0.41108900
H	-1.42230400	-2.43980400	-0.37376800
H	-3.00023000	-1.73218400	-0.04674000
H	-2.08867800	-1.19308900	-1.45537800
H	0.00000000	0.00000000	-1.01312800

B

C	2.29214700	4.06501100	-0.74661000
C	2.03218400	3.25639300	0.37735200
C	2.75850700	3.48667200	1.56287600
C	3.71499200	4.49900400	1.61888800
C	3.96462500	5.29487000	0.49777700
C	3.24956700	5.07515100	-0.68241300
C	1.03384600	2.21703000	0.31413000
C	0.00610400	1.50296900	0.26087000
Cu	-1.73483500	0.75184100	0.24794300
N	-3.41846500	0.92915800	-1.11189700
C	-4.52777300	0.32571800	-0.61803400

C	-5.77599800	0.34564500	-1.28754200
C	-5.83451500	1.03129900	-2.52258900
C	-4.69913400	1.64342400	-3.01592600
C	-3.50484700	1.56822500	-2.27310900
C	-6.90520800	-0.31272100	-0.69312000
C	-6.79495800	-0.95066600	0.50646200
C	-5.54583400	-0.98578700	1.21468100
C	-4.40992500	-0.35191400	0.65245700
N	-3.19668200	-0.34165900	1.26541500
C	-3.07486500	-0.94525200	2.44552800
C	-4.14455400	-1.60002800	3.08285900
C	-5.38014500	-1.62189500	2.46581300
H	-2.09298200	-0.90625000	2.90767500
H	-3.98702800	-2.07321500	4.04580600
H	-6.22691400	-2.11760500	2.93204900
H	-2.59522100	2.04250000	-2.63044000
H	-4.71335200	2.17957800	-3.95846200
H	-6.77220700	1.07248900	-3.06940100
H	-7.85529500	-0.28916900	-1.21840900
H	-7.65570700	-1.44188800	0.95038300
H	2.55457200	2.87371600	2.43546500
H	4.26116400	4.67184800	2.54153300
H	4.70801100	6.08479100	0.54513200
H	3.43608400	5.69468200	-1.55458100
H	1.73310600	3.89354200	-1.66092400
Cu	1.59296200	0.19241500	0.07938800
N	3.63472100	-0.29731200	-0.01985300
C	3.83371800	-1.62327100	-0.23752100
C	5.12501400	-2.19130700	-0.36380300
C	6.23041300	-1.31693200	-0.25367900
C	6.01484700	0.02839700	-0.03045700
C	4.69370000	0.50149400	0.08143800
C	5.25553300	-3.60266100	-0.59421000
C	4.15712400	-4.40357900	-0.69272200
C	2.83333600	-3.86130700	-0.56847900
C	2.66880700	-2.47227700	-0.34060000
N	1.44746600	-1.89207000	-0.21202200
C	0.36356300	-2.65525400	-0.30328000
C	0.42529200	-4.04267700	-0.52909500
C	1.66109100	-4.64514700	-0.66187800
H	-0.59095100	-2.14797200	-0.19159400
H	-0.49139900	-4.61816900	-0.59678900
H	1.74366700	-5.71395900	-0.83740100
H	4.49065600	1.55292700	0.26193500
H	6.84168400	0.72405900	0.05964100
H	7.23802100	-1.71238800	-0.34505000
H	6.25266600	-4.02185700	-0.68949200
H	4.26655000	-5.46968600	-0.86755400

C

C	0.40522500	-4.20949800	1.15911900
C	1.01044000	-3.49308000	0.10700500
C	1.87396200	-4.18109300	-0.76833200
C	2.12419200	-5.54174400	-0.59473900
C	1.52023800	-6.24048500	0.45323900
C	0.65962000	-5.56922700	1.32590500
C	0.73029500	-2.08775800	-0.06475900
C	0.17317600	-0.96585400	-0.16014500
Cu	-1.00153800	0.54968500	-0.25557200
N	-1.22605000	2.13417900	1.28851700
C	-1.70158700	3.29310300	0.77047000
C	-1.97555100	4.43446100	1.56752500
C	-1.71704100	4.33248700	2.95293400
C	-1.22219000	3.14983600	3.46787200
C	-0.99355800	2.06891800	2.59442600
C	-2.50395900	5.61785000	0.94919500
C	-2.75369600	5.65725200	-0.38994700
C	-2.48499500	4.52237200	-1.22830600
C	-1.94421000	3.34324600	-0.65481900
N	-1.64461800	2.24128200	-1.38966200
C	-1.90454200	2.25950400	-2.69336400
C	-2.45425100	3.37575300	-3.35067200
C	-2.73675000	4.51192200	-2.61842800
H	-1.67367300	1.35218300	-3.24302700
H	-2.64461300	3.33079800	-4.41740700
H	-3.15519200	5.39393800	-3.09501300
H	-0.61687500	1.12058900	2.96765000
H	-1.01368500	3.04052000	4.52674500
H	-1.91278100	5.18373100	3.59903800
H	-2.71124100	6.48160200	1.57415600
H	-3.16495800	6.55238600	-0.84734300
H	2.33118500	-3.64052800	-1.59212200
H	2.78560700	-6.05927400	-1.28371800
H	1.71552600	-7.30017700	0.58681500
H	0.18503100	-6.10764500	2.14122700
H	-0.26292900	-3.68663800	1.83604400
Cu	2.20089100	-0.59003400	-0.11228900
N	4.24624300	-1.03961800	0.13940600
C	5.02967400	0.06697700	0.05369700
C	6.43762600	0.02093600	0.20280900
C	7.02049200	-1.24128300	0.45765500
C	6.21333100	-2.35793600	0.54780400
C	4.82314700	-2.21391900	0.37957500
C	7.19865600	1.23364900	0.09035600
C	6.58806400	2.42714200	-0.15601400
C	5.16260400	2.51071700	-0.30815500
C	4.38194600	1.33317300	-0.20096400
N	3.03075200	1.33714900	-0.32738100
C	2.41059800	2.48769600	-0.56506200
C	3.10038200	3.70828900	-0.69201500
C	4.47559800	3.71960800	-0.56250300
H	1.32893600	2.43635400	-0.65574600
H	2.54723500	4.62028900	-0.88834500
H	5.03650400	4.64551700	-0.65376200
H	4.16200900	-3.07391700	0.43330800
H	6.62993500	-3.33993100	0.74294000
H	8.09704200	-1.32026800	0.57950200
H	8.27714600	1.18052900	0.20551300
H	7.17318400	3.33802500	-0.24061100
O	-3.11253700	-0.22403900	-0.43323400
N	-3.42585500	-1.48277100	-0.54525900
C	-3.70362900	-2.28501200	0.46016700
H	-3.89008000	-3.31208200	0.17057100
C	-3.78842300	-1.94741400	1.86471100
C	-4.03203400	-3.01452600	2.75876500
C	-3.67182900	-0.64028900	2.38640800
C	-4.14941200	-2.78892500	4.12483600
H	-4.13114100	-4.02426800	2.36867100

C	-3.80065300	-0.42533500	3.75699900
H	-3.49009100	0.17785900	1.70447000
C	-4.03428400	-1.49018000	4.63018500
H	-4.33621000	-3.62155500	4.79609000
H	-3.72067200	0.58571800	4.14641400
H	-4.13136700	-1.31136300	5.69692700
C	-3.47995000	-2.00237400	-1.90238400
C	-2.37570800	-1.81715200	-2.73613500
C	-4.63284400	-2.64521000	-2.35280600
C	-2.42953800	-2.30590900	-4.04018200
H	-1.48777000	-1.33306800	-2.34380700
C	-4.67590500	-3.12229900	-3.66449000
H	-5.49058200	-2.74857800	-1.69596300
C	-3.57686600	-2.95481000	-4.50743000
H	-1.56931500	-2.18775300	-4.69236900
H	-5.57266600	-3.61558500	-4.02611500
H	-3.61293400	-3.32802500	-5.52625500

TS$_{C-D}$

Cu	0.31347400	1.08797100	0.23176400
C	-1.44023200	3.67128600	3.36650200
C	-1.79469500	4.80972500	2.66993200
C	-1.60157300	4.85847100	1.27128500
C	-1.03951200	3.71406000	0.64781800
C	-0.88815600	2.58694300	2.66118700
C	-1.94503000	6.00248500	0.47395900
C	-0.82844100	3.71625700	-0.78413600
C	-1.18121000	4.86465100	-1.53949100
C	-1.74292600	6.00588300	-0.87360200
C	-0.95226500	4.81704500	-2.93342900
H	-1.20543700	5.67655900	-3.54786200
C	-0.40605800	3.67966900	-3.49471000
C	-0.08990000	2.59069400	-2.65875600
H	-2.36860900	6.87057900	0.97036700
H	-1.57628200	3.60102800	4.44011300
H	-2.21905100	5.66821600	3.18287900
H	-0.59566700	1.67947500	3.18144300
H	-2.00254600	6.87741700	-1.46741400
H	-0.21552100	3.61337500	-4.56042400
H	0.35451300	1.68733700	-3.06674100
N	-0.68839000	2.60245100	1.34565200
N	-0.29518300	2.60489500	-1.34696400
C	0.67791800	-0.77103300	-0.10491400
C	1.75940500	-1.43580000	-0.23048400
C	2.18046700	-2.82524300	-0.44555600
C	1.80352700	-3.49799900	-1.62334800
C	2.94686100	-3.51842900	0.50999200
C	2.17574800	-4.82608100	-1.83202600
H	1.22587000	-2.96424400	-2.37101500
C	3.31020400	-4.84881300	0.29833100
H	3.24249000	-3.01453400	1.42592700
C	2.92850700	-5.50755100	-0.87283100
H	1.88118000	-5.32717400	-2.74975600
H	3.89539300	-5.37056000	1.05009400
H	3.22084200	-6.53990300	-1.03899000
Cu	-1.23669600	-0.96716300	-0.08761200
C	-2.10340600	-5.31254500	0.73021400
C	-3.48132000	-5.32059700	0.64363200
C	-4.16979600	-4.11897500	0.35763500
C	-3.38515100	-2.95430500	0.17465000
C	-1.41032500	-4.10216800	0.52962200
C	-5.59862200	-4.03124700	0.24553600
C	-4.03246400	-1.69643100	-0.11907900
C	-5.44479400	-1.64549100	-0.22596300
C	-6.20986700	-2.84562800	-0.03472400
C	-6.02836600	-0.39292800	-0.52152100
H	-7.10790400	-0.31279200	-0.61253300
C	-5.21930300	0.71333700	-0.69365600
C	-3.82656600	0.56679300	-0.56682100
H	-6.18662700	-4.93302100	0.38806700
H	-1.54783700	-6.21825700	0.94677100
H	-4.04360000	-6.23818500	0.79153700
H	-0.32662100	-4.06060700	0.58256700
H	-7.29085400	-2.78931300	-0.11936200
H	-5.63805800	1.68662000	-0.92428200
H	-3.16048400	1.41386700	-0.69673400
N	-2.03225400	-2.95982200	0.26109600
N	-3.24665000	-0.59829800	-0.28685500
O	2.28194300	1.57192800	0.51336500
N	3.16696500	0.62700600	0.76753800
C	3.39160900	-0.32570400	-0.18710800
H	4.07173400	-1.09238400	0.16532000
C	3.63163500	0.03175300	-1.61255100
C	4.29377000	-0.92200300	-2.40721400
C	3.27986500	1.26286100	-2.19248300
C	4.58778400	-0.66098200	-3.74394700
H	4.58493400	-1.87351300	-1.97187300
C	3.59137000	1.52379600	-3.52704000
H	2.78483600	2.00718700	-1.58325300
C	4.23672000	0.56507400	-4.31109900
H	5.10175700	-1.41100200	-4.33765000
H	3.33387400	2.48911800	-3.95471800
H	4.47336800	0.77484900	-5.34998600
C	3.50360500	0.41090800	2.13790800
C	2.73890700	1.03771500	3.13059700
C	4.61709800	-0.36337300	2.50427700
C	3.05617600	0.84725800	4.47440800
H	1.92114700	1.67705800	2.82851700
C	4.91851100	-0.54923900	3.85159600
H	5.26988000	-0.79116600	1.75157700
C	4.13790400	0.04709300	4.84461900
H	2.45688600	1.33535100	5.23775000
H	5.78323900	-1.14688400	4.12357700
H	4.38395800	-0.09409400	5.89221100

D

Cu	1.09149800	0.45689200	0.37397800
C	2.17964300	3.53872300	3.31534700
C	2.90250700	4.44476300	2.56470500
C	3.05686000	4.23353100	1.17635500
C	2.44942000	3.07901500	0.61832400
C	1.61696900	2.42051400	2.67500700
C	3.79648200	5.12459000	0.32693900
C	2.58180000	2.82175600	-0.79967600
C	3.32373300	3.72167400	-1.60708400
C	3.92563300	4.87890500	-1.00726700
C	3.43170400	3.41527900	-2.98293800
H	3.99381900	4.07646400	-3.63649700

C	2.82674500	2.27639700	-3.47713300
C	2.10952300	1.44642400	-2.59290800
H	4.25786000	6.00042900	0.77345200
H	2.04408400	3.67071400	4.38309500
H	3.35656100	5.31609600	3.02816800
H	1.05057000	1.68596400	3.23961400
H	4.49251900	5.55609300	-1.63941500
H	2.89763600	2.01123900	-4.52639900
H	1.63267000	0.53587900	-2.94462200
N	1.74471000	2.19144100	1.36990000
N	1.98595100	1.71218000	-1.29845900
C	-0.08789600	-0.94109100	-0.12983000
C	0.14240700	-2.22152400	-0.28115700
C	-0.92495000	-3.19411000	-0.66679800
C	-1.62655400	-3.03689500	-1.87419400
C	-1.26512900	-4.26825100	0.17508900
C	-2.64116800	-3.92714400	-2.22891500
H	-1.35717100	-2.21925900	-2.53490900
C	-2.28650500	-5.15197100	-0.17891800
H	-0.74169900	-4.39896100	1.11829200
C	-2.97710100	-4.98615200	-1.38227400
H	-3.16380800	-3.79757200	-3.17237100
H	-2.53937900	-5.97284700	0.48589800
H	-3.76222200	-5.68212300	-1.66178100
Cu	-1.48525700	0.36101000	-0.16746900
C	-5.45645000	-1.61302400	0.56807100
C	-6.30379900	-0.52632000	0.48826000
C	-5.76983200	0.75675400	0.22685500
C	-4.36658100	0.84976400	0.06119500
C	-4.07276000	-1.42108500	0.38394100
C	-6.57225800	1.94291800	0.12363900
C	-3.76383000	2.13490900	-0.20476100
C	-4.58495000	3.28582700	-0.30355200
C	-6.00454800	3.15557700	-0.13130300
C	-3.94641600	4.51730000	-0.57184800
H	-4.54129800	5.42223600	-0.65587700
C	-2.57427600	4.55525700	-0.72612300
C	-1.84091000	3.36147300	-0.60899600
H	-7.64690500	1.85504200	0.25255400
H	-5.83556800	-2.60931600	0.76610600
H	-7.37532900	-0.64374500	0.62283400
H	-3.38080800	-2.25587600	0.43212100
H	-6.61935900	4.04697300	-0.20941000
H	-2.05675900	5.48483000	-0.93524100
H	-0.76181000	3.35544800	-0.72548400
N	-3.54483000	-0.22661100	0.13946600
N	-2.41222300	2.18598900	-0.35493000
O	2.49771700	-0.90869200	0.76089700
N	2.27240800	-2.19615600	0.99458800
C	1.59636400	-2.88642700	-0.05820100
H	1.39415500	-3.90164800	0.28124100
C	2.33322300	-2.98648200	-1.38833800
C	2.03286200	-4.07977900	-2.21488600
C	3.28632800	-2.05430600	-1.82282900
C	2.65894400	-4.23613400	-3.45184500
H	1.30675700	-4.81901000	-1.88754000
C	3.92090600	-2.22147600	-3.05532800
H	3.53545500	-1.21763000	-1.18275800
C	3.60711300	-3.30531300	-3.87766900
H	2.41413000	-5.09176900	-4.07441100
H	4.67323100	-1.50274200	-3.36928300
H	4.10451000	-3.42975100	-4.83509300
C	2.08345800	-2.57557500	2.34646000
C	2.07780800	-1.58984400	3.34664300
C	1.97533300	-3.92957500	2.71797400
C	1.91908800	-1.95183000	4.68326700
H	2.21856300	-0.55809000	3.05600100
C	1.81415500	-4.27429000	4.05725200
H	2.05439300	-4.71683100	1.97616700
C	1.77683400	-3.29082200	5.04932600
H	1.91527600	-1.17853300	5.44662100
H	1.73618200	-5.32321100	4.32766200
H	1.65933700	-3.56760700	6.09210800

TS$_{D-E}$

Cu	-1.02702000	-0.72836400	-0.00075000
C	-3.67682000	0.06625300	-3.47194400
C	-4.92570700	0.10698800	-2.88319500
C	-5.04455800	-0.09039300	-1.48908800
C	-3.84974400	-0.31344000	-0.75913000
C	-2.54944600	-0.17401800	-2.66534900
C	-6.30420100	-0.09029300	-0.79851300
C	-3.91940600	-0.53199700	0.66829400
C	-5.18166700	-0.55405900	1.31339100
C	-6.37103700	-0.31869100	0.54365500
C	-5.19253300	-0.82061000	2.70159300
H	-6.13879600	-0.85046200	3.23453300
C	-4.00074800	-1.05285800	3.36118200
C	-2.79619400	-0.99851600	2.63390400
H	-7.20923800	0.08369900	-1.37304000
H	-3.55239600	0.20743300	-4.53993300
H	-5.81732800	0.28231300	-3.47854000
H	-1.55581600	-0.22538000	-3.10038600
H	-7.33012900	-0.33114100	1.05296100
H	-3.97894300	-1.27609200	4.42227000
H	-1.84057900	-1.18441400	3.11577500
N	-2.62767100	-0.35801100	-1.35045100
N	-2.75473100	-0.73145200	1.33261000
C	0.91152400	-0.57778500	0.03864400
C	2.00913800	-1.28471700	-0.15713400
C	3.26152300	-0.69185300	-0.71820200
C	4.08615000	0.12309800	0.07335900
C	3.64511800	-0.96268400	-2.04249800
C	5.26387900	0.65968500	-0.45045100
H	3.80735900	0.31495100	1.10448400
C	4.81946900	-0.41836600	-2.56591400
H	3.01757500	-1.59848600	-2.66135500
C	5.63276700	0.39435900	-1.77122700
H	5.89799800	1.27814100	0.17829900
H	5.10248700	-0.63614000	-3.59180300
H	6.55280600	0.80654600	-2.17497200
Cu	0.55365800	1.31937800	0.19724500
C	3.12756600	3.96938800	-2.36124500
C	2.71743000	5.21885100	-1.94182200
C	1.71799800	5.32683500	-0.94720400
C	1.18476100	4.11958100	-0.43326800
C	2.54053300	2.82484800	-1.78631800
C	1.23340900	6.58222600	-0.44617100

C	0.16124400	4.16960400	0.58489200
C	-0.29262900	5.42556500	1.05985400
C	0.26976500	6.62990700	0.51666500
C	-1.29041800	5.41858900	2.06058700
H	-1.66300100	6.36073300	2.45258200
C	-1.77831800	4.21432900	2.52886100
C	-1.26853400	3.01749400	1.99462200
H	1.65309700	7.49869900	-0.85033600
H	3.89436600	3.85299800	-3.11898800
H	3.15338300	6.12005200	-2.36368300
H	2.84997200	1.82888700	-2.08678200
H	-0.08894900	7.58383700	0.89117700
H	-2.54219200	4.17685100	3.29750800
H	-1.63322600	2.05426600	2.33723300
N	1.59659100	2.89713300	-0.85373400
N	-0.32830900	2.99039700	1.05290500
O	-0.02791900	-2.35698900	0.94826800
N	0.71342600	-3.25579200	0.28776100
C	2.08504000	-2.79885800	0.22928600
H	2.61647500	-3.33108400	-0.56146400
C	2.86147200	-2.99238300	1.52674700
C	3.98991400	-3.81972500	1.52869900
C	2.47729700	-2.36407100	2.71953400
C	4.72184000	-4.02553800	2.69982500
H	4.30044500	-4.31030500	0.60927500
C	3.20307000	-2.57611000	3.89099100
H	1.60293100	-1.72336300	2.71987200
C	4.32825200	-3.40485700	3.88493700
H	5.59540100	-4.67045300	2.68406900
H	2.89205200	-2.09158800	4.81235400
H	4.89397400	-3.56388200	4.79817200
C	0.10221800	-3.91501200	-0.80273400
C	-1.29459800	-3.83964500	-0.95206100
C	0.83711100	-4.72097700	-1.69399900
C	-1.92577200	-4.50738700	-2.00153100
H	-1.87220600	-3.29182200	-0.21941200
C	0.19178000	-5.37769400	-2.73822700
H	1.90407700	-4.86289200	-1.56681000
C	-1.19117600	-5.27030500	-2.90894800
H	-3.00663500	-4.44380200	-2.09526100
H	0.77738300	-5.99105200	-3.41681700
H	-1.68718800	-5.79499900	-3.71920900

E

Cu	-1.59503300	-0.14016400	0.38431300
C	-3.53980900	-3.28718300	2.68022300
C	-4.77751400	-3.37738300	2.07415300
C	-5.08416500	-2.53250100	0.98410300
C	-4.08402800	-1.62048800	0.56271000
C	-2.60917600	-2.35311500	2.19289600
C	-6.34684800	-2.56168500	0.30037400
C	-4.35368500	-0.73161800	-0.54538800
C	-5.60937400	-0.78987700	-1.19940400
C	-6.59859200	-1.72700700	-0.74719400
C	-5.81477900	0.09902300	-2.28023400
H	-6.76375700	0.08888100	-2.80894700
C	-4.80739100	0.96830600	-2.64956400
C	-3.59343600	0.95152300	-1.93380700
H	-7.10109900	-3.26629700	0.63748700
H	-3.27588000	-3.91967900	3.52046400
H	-5.51953000	-4.08831600	2.42618600
H	-1.62924800	-2.25397300	2.64926000
H	-7.55661200	-1.75789100	-1.25765400
H	-4.93603800	1.66135000	-3.47377900
H	-2.77872200	1.62385900	-2.18802100
N	-2.86587700	-1.54415300	1.16612400
N	-3.37559100	0.13038000	-0.91320800
C	-0.08248700	1.10280200	0.14967400
C	0.85908800	1.64735300	0.98342100
C	1.19910400	1.19727700	2.34713100
C	2.50835000	1.29935400	2.85818600
C	0.20568000	0.65960700	3.19323600
C	2.81417100	0.85357200	4.14426000
H	3.29426100	1.71514800	2.23786800
C	0.51707700	0.20155700	4.47306300
H	-0.82299600	0.63950400	2.84675400
C	1.82534900	0.29331900	4.95629100
H	3.83175100	0.94647100	4.51296600
H	-0.27057000	-0.19412900	5.10887300
H	2.06466300	-0.04348900	5.96052200
Cu	0.89594100	-0.65475500	-0.12260600
C	3.19584400	-3.55047600	2.25667900
C	3.71387700	-4.37086000	1.27405500
C	3.41240000	-4.11125900	-0.08234400
C	2.57498300	-3.00287100	-0.36157800
C	2.36791000	-2.47476800	1.88517300
C	3.91500000	-4.90775100	-1.16645300
C	2.25411600	-2.68097800	-1.73469500
C	2.77130300	-3.48601100	-2.78028400
C	3.60611200	-4.60948800	-2.45993200
C	2.43358900	-3.12283100	-4.10417600
H	2.81024700	-3.71373800	-4.93427800
C	1.63343100	-2.01917600	-4.32647600
C	1.17015000	-1.27941200	-3.22202400
H	4.55236800	-5.75558100	-0.93367800
H	3.41613000	-3.71885400	3.30502400
H	4.35435500	-5.21036400	1.52927400
H	1.94843500	-1.80862800	2.63321500
H	3.99312200	-5.21667100	-3.27279400
H	1.36172700	-1.71340200	-5.33083200
H	0.54656300	-0.40024400	-3.35855700
N	2.06075900	-2.21175300	0.61686800
N	1.46830600	-1.59898700	-1.96683900
O	-0.26567100	1.89908300	-0.97639400
N	0.48013800	3.14522900	-0.79821000
C	1.43526200	2.90722200	0.32519800
H	1.37180200	3.75521200	1.01670000
C	2.87185400	2.80028200	-0.17040200
C	3.86772500	3.61487700	0.37983000
C	3.21822000	1.90628000	-1.19255400
C	5.18953300	3.52741300	-0.06554700
H	3.60852100	4.32472400	1.16205000
C	4.53459400	1.82211200	-1.64393900
H	2.44720000	1.29292000	-1.64932900
C	5.52636800	2.62891600	-1.07759600
H	5.95024000	4.16691300	0.37247400
H	4.78861100	1.13385000	-2.44559100
H	6.55115500	2.56325100	-1.43076400

C	-0.42004600	4.24090200	-0.61998100
C	-1.79523200	4.09066800	-0.40383200
C	0.13221800	5.52971500	-0.69391500
C	-2.60165700	5.22163400	-0.25669600
H	-2.22296100	3.09820000	-0.34283700
C	-0.68021500	6.64892100	-0.52898000
H	1.19293000	5.64273000	-0.89583500
C	-2.05295200	6.50261700	-0.31333600
H	-3.66748700	5.09530600	-0.08633400
H	-0.23995500	7.64008000	-0.58650400
H	-2.68585800	7.37674500	-0.19683900

F

C	-3.73575000	-0.43643500	2.16824900
C	-3.97576000	-0.53073400	0.79136900
C	-5.16359200	-1.12419400	0.35441600
C	-6.09448800	-1.62347400	1.26975200
C	-5.84615300	-1.52655400	2.63817100
C	-4.66323800	-0.92803500	3.08421100
C	-2.94993200	-0.00871000	-0.21159700
N	-2.54976300	1.38230700	0.09091700
C	-3.01924100	2.43341700	-0.71093800
C	-2.20123900	3.52148100	-1.06670300
C	-2.72436600	4.57305300	-1.81619700
C	-4.05967500	4.57567700	-2.22430500
C	-4.87412100	3.50082800	-1.86358300
C	-4.37005700	2.44050700	-1.11315800
O	-1.08520200	1.41761200	0.21942000
Cu	1.31870900	-0.02220700	0.11584300
N	2.89924000	1.38316200	0.81229200
C	4.13615000	0.92956700	0.50206100
C	5.31788500	1.65912300	0.78402300
C	5.16270200	2.91173800	1.42058000
C	3.89564000	3.36445400	1.73303900
C	2.78303800	2.56296300	1.40716900
C	4.22811100	-0.35871800	-0.14712000
C	5.50009500	-0.87626900	-0.49634100
C	6.67740000	-0.10932600	-0.19741900
C	6.59078300	1.10507400	0.41639100
C	5.53323200	-2.13928700	-1.12919500
C	4.34992500	-2.80772700	-1.37474200
C	3.13294700	-2.21408000	-0.98909600
N	3.07055800	-1.02456100	-0.39531300
C	-0.60246700	0.14236500	0.06318100
C	-1.60737700	-0.74079600	-0.17585700
C	-1.46724500	-2.17393600	-0.42900700
C	-2.36724900	-2.87065900	-1.26111800
C	-2.20665700	-4.22928900	-1.53128200
C	-1.14455500	-4.94478300	-0.97615600
C	-0.25158800	-4.27874400	-0.13047600
C	-0.41701600	-2.92355300	0.14792800
H	1.77077300	2.88758800	1.63245800
H	3.74463800	4.32150700	2.22068700
H	6.04146400	3.50579700	1.65647100
H	2.18446400	-2.71672800	-1.15702800
H	4.34089700	-3.77902100	-1.85723600
H	6.48843900	-2.57149000	-1.41468500
H	7.64496000	-0.51939900	-0.47261300
H	7.48852200	1.67567000	0.63693000
H	0.25186900	-2.42585100	0.84372900
H	0.56108100	-4.82726000	0.34131200
H	-1.02526400	-6.00485700	-1.18160200
H	-2.91755000	-4.73131300	-2.18276000
H	-3.19464800	-2.33693700	-1.71764800
H	-3.40777100	-0.04962200	-1.21468900
H	-1.16345500	3.52352000	-0.76154200
H	-2.07300600	5.40019600	-2.08765400
H	-4.45855900	5.40012000	-2.80760200
H	-5.91892300	3.48405900	-2.16307200
H	-5.02595200	1.63003600	-0.81518200
H	-5.36555400	-1.19379400	-0.71248400
H	-7.01201800	-2.08285500	0.91194800
H	-6.56804100	-1.91055700	3.35375300
H	-4.46569000	-0.84520600	4.14979800
H	-2.81871400	0.03350200	2.51110600

F’

C	-2.78274200	1.46095400	-2.08514200
C	-3.09351400	1.64463800	-0.73070500
C	-4.30758700	2.26060100	-0.39853000
C	-5.20279000	2.66540000	-1.39156700
C	-4.89322200	2.45667400	-2.73684700
C	-3.67810700	1.85712400	-3.08110300
C	-2.13815700	1.20258200	0.37274700
N	-2.26286200	-0.26866800	0.69069000
C	-2.33925700	-0.53949400	2.10995800
C	-1.30426300	-1.14991300	2.82686200
C	-1.46878500	-1.40908800	4.19023500
C	-2.65036700	-1.06548500	4.84728700
C	-3.68179100	-0.45732100	4.12822800
C	-3.53390600	-0.20235500	2.76514000
O	-1.02208300	-0.88521400	0.15581200
Cu	1.75049100	-0.42320900	-0.36755000
N	3.33646300	-1.55767900	-1.02581300
C	4.56190200	-1.19993000	-0.55338500
C	5.74552700	-1.90068300	-0.89775200
C	5.61877600	-3.00486800	-1.76933500
C	4.37121200	-3.35625500	-2.24613800
C	3.25324200	-2.60341400	-1.84689900
C	4.64265600	-0.05826300	0.32942600
C	5.90186500	0.34134600	0.84218400
C	7.07910300	-0.39464700	0.47720100
C	7.00425800	-1.46875700	-0.35808900
C	5.92078100	1.46714300	1.69721000
C	4.73969300	2.12061800	1.98786700
C	3.53846300	1.64595200	1.42363100
N	3.49073200	0.58908000	0.62265900
C	-0.08488600	0.12268300	-0.09610800
C	-0.65825700	1.34333700	0.03389500
C	-0.02195900	2.65494700	-0.15407400
C	-0.48638400	3.79851400	0.52485400
C	0.13100200	5.03844400	0.36146800
C	1.22963900	5.17510900	-0.48811900
C	1.69617200	4.05569100	-1.18264800
C	1.07411300	2.81905600	-1.02702000
H	2.25962400	-2.85606200	-2.20321600
H	4.24033200	-4.19740000	-2.91789800
H	6.50361900	-3.56578200	-2.05645800

H	2.59346600	2.14429600	1.61946500
H	4.72226200	2.98989800	2.63622600
H	6.86432300	1.80826400	2.11379100
H	8.03629500	-0.07603300	0.87928900
H	7.89987200	-2.01847800	-0.63156600
H	1.41998500	1.96417400	-1.60079900
H	2.53555300	4.15106300	-1.86638400
H	1.70586500	6.14196000	-0.61977400
H	-0.24992800	5.90090800	0.90110200
H	-1.33423400	3.71957900	1.19896500
H	-2.40891400	1.74139400	1.28675500
H	-0.38552300	-1.41144600	2.31805300
H	-0.65867000	-1.87831900	4.74080700
H	-2.76819100	-1.26875700	5.90690300
H	-4.60760100	-0.18405500	4.62599100
H	-4.34052700	0.26699400	2.20874000
H	-4.54674800	2.44324400	0.64700300
H	-6.13281600	3.15399300	-1.11564400
H	-5.58164200	2.77992600	-3.51209500
H	-3.41733700	1.72116400	-4.12711900
H	-1.81674400	1.04251400	-2.35248500
N	-3.78435000	-2.31528500	-0.70056800
C	-4.86184200	-1.72492100	-1.58930000
H	-5.21228700	-2.53794400	-2.23326900
C	-2.74756400	-3.07017600	-1.50893300
H	-3.23168400	-3.99464900	-1.84068600
C	-4.38217700	-3.19937700	0.37836100
H	-5.05852900	-3.89207000	-0.13260400
H	-4.97922600	-2.54133000	1.00943500
C	-3.36900400	-3.95197300	1.23320400
H	-2.63342400	-3.27936200	1.68106600
H	-3.91570200	-4.43113700	2.05030700
H	-2.85013800	-4.73970800	0.68269000
C	-2.16071600	-2.29704500	-2.68217900
H	-1.36809400	-2.91180900	-3.11900300
H	-2.88698500	-2.09405100	-3.47198500
H	-1.70809600	-1.36292400	-2.34578300
H	-1.95205300	-3.32058600	-0.80900500
H	-4.36633400	-0.97742000	-2.20865100
C	-6.02062400	-1.07881200	-0.83879300
H	-5.67088100	-0.31874500	-0.13602800
H	-6.64968700	-0.56861500	-1.57287700
H	-6.64693600	-1.80210600	-0.31249700
H	-3.29274200	-1.50617400	-0.21965500

TS$_{F'-G}$

C	-2.76741100	1.55052200	-2.06846200
C	-3.15773400	1.54023300	-0.72265000
C	-4.42806500	2.02702300	-0.38403300
C	-5.30349900	2.48584900	-1.36988500
C	-4.91399900	2.46753000	-2.71044000
C	-3.64243300	2.00308800	-3.05665000
C	-2.21352700	1.08117200	0.37756500
N	-2.23986200	-0.43155600	0.64266000
C	-2.41275800	-0.73297000	2.07078000
C	-1.33479100	-1.09258200	2.87863600
C	-1.56035600	-1.34944400	4.23322000
C	-2.84232300	-1.24740400	4.77422900
C	-3.91279200	-0.88284200	3.95515400
C	-3.70226700	-0.62466700	2.60032900
O	-0.90386100	-0.93333300	0.20678600
Cu	1.81630700	-0.28971900	-0.21745500
N	3.31762200	-1.55961100	-0.90308700
C	4.57628800	-1.20239500	-0.53317200
C	5.72049600	-1.96073800	-0.88668000
C	5.51420100	-3.12629300	-1.65808600
C	4.23190600	-3.47743200	-2.03172600
C	3.15795600	-2.66312100	-1.63012500
C	4.73388400	0.00275700	0.24836200
C	6.02946800	0.40651400	0.65612100
C	7.16631200	-0.38827600	0.28522000
C	7.01833400	-1.52260300	-0.45562000
C	6.12508900	1.59452200	1.41653900
C	4.97881300	2.30161600	1.72066700
C	3.73614100	1.81970200	1.26454600
N	3.61495100	0.70440500	0.55338000
C	-0.05436700	0.15928700	0.01128800
C	-0.73168100	1.32480900	0.11636800
C	-0.19203000	2.68653600	-0.02026700
C	-0.77894600	3.77761100	0.64794100
C	-0.25134500	5.06361800	0.53264700
C	0.87618000	5.29687700	-0.25584900
C	1.46363700	4.22833600	-0.93858600
C	0.93122800	2.94530700	-0.83161000
H	2.13861900	-2.91452200	-1.90606400
H	4.04066100	-4.36494100	-2.62470900
H	6.36593300	-3.73397800	-1.95011100
H	2.81801100	2.36095100	1.47204000
H	5.01957500	3.21826900	2.29876500
H	7.09910900	1.94012300	1.75124600
H	8.15216400	-0.06529600	0.60630700
H	7.88389700	-2.11638700	-0.73374500
H	1.37135800	2.12840000	-1.39565700
H	2.32704400	4.39904000	-1.57595200
H	1.28259800	6.29928700	-0.34983100
H	-0.72465900	5.88560900	1.06198400
H	-1.65272000	3.62449100	1.27462100
H	-2.54536600	1.53431200	1.31616400
H	-0.34386200	-1.17468400	2.45204200
H	-0.72395100	-1.62993700	4.86581500
H	-3.00768900	-1.45019600	5.82753000
H	-4.91378600	-0.79917700	4.36663500
H	-4.53337600	-0.33381900	1.96570500
H	-4.72821100	2.06679800	0.66099400
H	-6.28071100	2.86816700	-1.09044100
H	-5.58921100	2.83185600	-3.47881500
H	-3.32468100	2.01231800	-4.09506400
H	-1.76462400	1.23150100	-2.33259100
N	-3.59087000	-2.23717200	-0.78449100
C	-4.73948800	-1.69534400	-1.58270000
H	-5.05163300	-2.46850600	-2.30046100
C	-2.58592800	-2.85857500	-1.70989700
H	-3.04519000	-3.75515800	-2.15359900
C	-4.08794500	-3.28492900	0.17023000
H	-4.72826800	-3.98287400	-0.38929100
H	-4.72146900	-2.77869500	0.89986100
C	-3.00881700	-4.07121800	0.91433500
H	-2.28039800	-3.41634600	1.39869800

H	-3.49376000	-4.66091200	1.69774600
H	-2.47447300	-4.76994900	0.26705100
C	-2.04808500	-1.95535600	-2.81492300
H	-1.29593800	-2.51801500	-3.37705000
H	-2.81394400	-1.64078500	-3.52717700
H	-1.55914600	-1.07130300	-2.40285600
H	-1.74795600	-3.18439100	-1.09358900
H	-4.36143400	-0.84737400	-2.15558300
C	-5.95283000	-1.24204100	-0.77304000
H	-5.68478100	-0.50369300	-0.01329500
H	-6.65609100	-0.75503200	-1.45424400
H	-6.48095100	-2.06921700	-0.29340500
H	-2.94466900	-1.16792800	-0.03564500

G

C	-4.41822000	-0.64995400	-1.88266800
C	-4.25209000	-0.75705500	-0.49706600
C	-5.31832600	-1.22021900	0.28645200
C	-6.53276100	-1.56819700	-0.30540100
C	-6.69041700	-1.46020500	-1.68868900
C	-5.63195000	-1.00132700	-2.47506600
C	-2.91613700	-0.44943000	0.17365900
N	-2.08375600	-1.66673000	0.12624100
C	-1.13011400	-1.98530400	1.06750200
C	-0.57524400	-1.00979000	1.94428200
C	0.37103000	-1.39940000	2.92775800
C	0.82656100	-2.72349900	3.00148600
C	0.28792600	-3.67232800	2.12152100
C	-0.66948100	-3.31833900	1.17758800
O	-0.11370600	0.21775100	-1.75894100
Cu	1.45979300	-0.67296000	1.12947300
N	2.66586700	-1.37339900	-0.41847100
C	3.61089300	-0.46006200	-0.76447600
C	4.54069100	-0.69115700	-1.80887900
C	4.45473800	-1.92341900	-2.49626500
C	3.48881400	-2.84110700	-2.13367600
C	2.60957300	-2.52388700	-1.08306300
C	3.65900200	0.78727600	-0.03258000
C	4.63709100	1.75586400	-0.37156600
C	5.56339900	1.48679000	-1.43475700
C	5.51685600	0.31291200	-2.12453000
C	4.64689500	2.95883500	0.37064600
C	3.72279300	3.14636400	1.37924300
C	2.78933600	2.12826300	1.64293600
N	2.75433300	0.98545300	0.96309500
C	-1.08852800	0.45616900	-1.14989900
C	-2.15793100	0.74164200	-0.41870100
C	-2.55178100	2.14820000	-0.16561900
C	-3.77005800	2.44754400	0.46666000
C	-4.12768300	3.77169700	0.72825700
C	-3.28854400	4.82096200	0.35809400
C	-2.07796900	4.53446500	-0.27956500
C	-1.71116900	3.21708700	-0.53340800
H	1.83939500	-3.22310900	-0.77189800
H	3.39748100	-3.79472700	-2.64162000
H	5.15016200	-2.13586000	-3.30331000
H	2.05071800	2.24868500	2.42983400
H	3.70437500	4.05758900	1.96669300
H	5.38203200	3.72438100	0.13962100
H	6.30486800	2.24140800	-1.67913800
H	6.22033100	0.11666400	-2.92798000
H	-0.76309900	3.01604400	-1.02741200
H	-1.41552500	5.34145800	-0.57888700
H	-3.57353100	5.84906400	0.55786400
H	-5.07529100	3.97817600	1.21693300
H	-4.45708400	1.65416300	0.73875800
H	-3.12159800	-0.20664300	1.22717300
H	-0.98527200	-0.00578800	1.96516800
H	0.68033400	-0.67469500	3.67626300
H	1.54915200	-3.01327200	3.75611100
H	0.60670100	-4.70832400	2.18728400
H	-1.09582700	-4.07856000	0.52821200
H	-5.19871900	-1.30448000	1.36433200
H	-7.35418900	-1.91837500	0.31223900
H	-7.63619000	-1.72637400	-2.15068800
H	-5.75297700	-0.90936900	-3.55015200
H	-3.60447600	-0.27409500	-2.49528700
H	-2.53438500	-2.45372800	-0.32063200

TS$_{G-I}$

C	-4.85707400	-0.93022900	-1.39493500
C	-4.43674200	-1.05266400	-0.06323300
C	-5.23209500	-1.76601300	0.84433300
C	-6.42735700	-2.35187500	0.42753700
C	-6.83679800	-2.23081100	-0.90223200
C	-6.05113100	-1.51896300	-1.81151500
C	-3.11570400	-0.46962800	0.39115200
N	-1.94609400	-1.35842100	-0.03413800
C	-0.95793400	-1.83196500	0.86653300
C	-0.28757300	-0.91919700	1.71638600
C	0.72445000	-1.39161900	2.57236600
C	1.09616800	-2.73766900	2.55998800
C	0.44251200	-3.62529000	1.70177100
C	-0.57238700	-3.17889700	0.85425900
O	-0.29695600	0.41063400	-1.25995300
Cu	1.24932700	-0.22299800	0.13077600
N	2.92240600	-1.14769700	-0.73884300
C	4.03519000	-0.37579100	-0.61367100
C	5.28842600	-0.76420900	-1.15269500
C	5.35090800	-1.99960400	-1.83626100
C	4.21286400	-2.77158500	-1.95551000
C	3.01375000	-2.30440000	-1.38896700
C	3.92530500	0.88559600	0.09027600
C	5.07246800	1.70958600	0.22208800
C	6.32409400	1.28141200	-0.33508300
C	6.42790600	0.09393400	-0.99384100
C	4.92026900	2.93641500	0.90719000
C	3.68470200	3.28636500	1.41354600
C	2.60440700	2.40380900	1.23538700
N	2.71513900	1.24091300	0.59945500
C	-1.41617500	0.34196900	-0.80325700
C	-2.58242900	0.77599700	-0.28064900
C	-3.11997000	2.13720200	-0.28363700
C	-4.31618200	2.42278600	0.39871700
C	-4.82641400	3.72051900	0.42164200
C	-4.15853900	4.75390000	-0.23584600
C	-2.97177500	4.47898100	-0.92188900
C	-2.45599300	3.18677300	-0.94848300

H	2.10178700	-2.88850400	-1.46615400
H	4.22735400	-3.72252000	-2.47636000
H	6.29429700	-2.32790700	-2.26289800
H	1.62018400	2.65427900	1.62075300
H	3.53389200	4.22171400	1.94094200
H	5.77751200	3.59279500	1.02610500
H	7.18945400	1.92733300	-0.22156400
H	7.37742500	-0.22306600	-1.41434400
H	-1.53912100	2.98333500	-1.49525900
H	-2.44876700	5.27503900	-1.44381200
H	-4.56001800	5.76230600	-0.21986000
H	-5.75210300	3.92111600	0.95288200
H	-4.85553100	1.62798400	0.90507700
H	-3.11020600	-0.40131500	1.48645500
H	-0.65366500	0.10077100	1.81043900
H	1.20258800	-0.69910100	3.25788600
H	1.87775900	-3.09304300	3.22306100
H	0.71495500	-4.67615100	1.69712000
H	-1.08598300	-3.87936700	0.20102200
H	-4.91711800	-1.85818600	1.88127800
H	-7.03952700	-2.89710100	1.13941100
H	-7.76924700	-2.68256900	-1.22666200
H	-6.37323300	-1.41328300	-2.84296800
H	-4.25801700	-0.35690800	-2.09678800
H	-2.25655400	-2.07838000	-0.68275600

I

C	-4.65538400	0.77839900	-1.70268000
C	-4.69136100	0.24331200	-0.40622600
C	-5.92783500	-0.08691200	0.16661600
C	-7.11089000	0.11107000	-0.54497200
C	-7.06655100	0.63881700	-1.83712400
C	-5.83852200	0.97226300	-2.41429600
C	-3.42247400	-0.01101300	0.36091500
N	-2.64249400	-1.29166300	-0.13779600
C	-2.50851500	-2.45036500	0.73803700
C	-1.85382600	-2.31196700	1.96256500
C	-1.74798200	-3.42152000	2.79953000
C	-2.28626900	-4.65187900	2.41218500
C	-2.93385300	-4.77583700	1.18229600
C	-3.04869100	-3.67067800	0.33724800
O	-0.31946400	-0.73031300	-0.73869700
Cu	1.46751300	-0.12368600	-0.37099800
N	3.21166800	-1.26994200	-1.03210200
C	4.33707200	-0.66520000	-0.57611800
C	5.63358700	-1.18590300	-0.81614500
C	5.72273300	-2.38215000	-1.56467200
C	4.56968800	-2.98612600	-2.02510200
C	3.32721100	-2.39177300	-1.73280700
C	4.19174300	0.55956700	0.18057100
C	5.34796200	1.21673600	0.67081800
C	6.64466900	0.65744800	0.41143600
C	6.78213200	-0.49496800	-0.30227000
C	5.15932300	2.41205500	1.40106600
C	3.88121200	2.89307200	1.60390800
C	2.79094700	2.17623700	1.08024200
N	2.93691900	1.04345900	0.39363100
C	-1.42927000	-0.30972300	-0.33131500
C	-2.15863300	0.77244700	0.06783000
C	-1.87387700	2.19162900	0.19473900
C	-2.75484900	3.03622600	0.89627100
C	-2.48526700	4.39719800	1.02809100
C	-1.33331000	4.94791300	0.46279100
C	-0.45456800	4.12325700	-0.24551800
C	-0.71984900	2.76184700	-0.38255900
H	2.40128600	-2.84219500	-2.07944100
H	4.60577300	-3.90287300	-2.60327600
H	6.69830300	-2.81332300	-1.77076600
H	1.77487200	2.53485800	1.21213800
H	3.70322200	3.80888500	2.15653700
H	6.02289900	2.94162700	1.79327000
H	7.51766100	1.17545800	0.79681800
H	7.76683600	-0.91025400	-0.49484800
H	-0.05041200	2.13699200	-0.96731300
H	0.43169000	4.54634600	-0.71072800
H	-1.12871600	6.00945600	0.56172300
H	-3.17966900	5.03032800	1.57239100
H	-3.65816200	2.62483500	1.33768300
H	-3.64333900	-0.18588000	1.41938100
H	-1.42880900	-1.35526000	2.25027600
H	-1.24168800	-3.32497700	3.75460700
H	-2.19894200	-5.51227800	3.06786500
H	-3.35242100	-5.72947000	0.87742100
H	-3.55658800	-3.76213100	-0.61951100
H	-5.96308000	-0.49454700	1.17413500
H	-8.06408600	-0.14233700	-0.09135000
H	-7.98703000	0.79681600	-2.39081100
H	-5.80413300	1.39249200	-3.41476200
H	-3.70227700	1.05989300	-2.14167000
H	-3.02701300	-1.58691000	-1.03947100

TS$_{E-J}$

Cu	-1.53406700	-0.22403800	0.22564700
C	-2.93086000	-3.60874600	2.62740600
C	-4.17301900	-3.85085700	2.07467200
C	-4.62568800	-3.05549600	0.99784700
C	-3.76161300	-2.03195500	0.53429400
C	-2.14098200	-2.57154100	2.10098400
C	-5.90437700	-3.23970200	0.37004300
C	-4.18684500	-1.18915000	-0.56201700
C	-5.45495500	-1.39687900	-1.15743400
C	-6.30249900	-2.44497800	-0.66336600
C	-5.81342900	-0.53884300	-2.22374100
H	-6.77796900	-0.66205400	-2.70815300
C	-4.93599400	0.44513100	-2.63609200
C	-3.69501100	0.57384800	-1.98053100
H	-6.55227600	-4.02887800	0.73935200
H	-2.55766000	-4.19933300	3.45657500
H	-4.80804900	-4.64396800	2.45901700
H	-1.16146600	-2.35372800	2.51463600
H	-7.27219500	-2.59279300	-1.12928400
H	-5.18647400	1.11676100	-3.44990700
H	-2.96915700	1.33066100	-2.26789600
N	-2.53721500	-1.80542700	1.08676000
N	-3.33831500	-0.21934400	-0.97792200
C	-0.38377200	1.33860000	-0.31514500
C	0.52919100	1.62921800	0.69274200
C	0.20756800	1.76295200	2.14133500

C	1.19937600	1.55130700	3.11665300
C	-1.07125500	2.16096100	2.57990400
C	0.92454800	1.71886900	4.47385700
H	2.19558400	1.26091400	2.79691700
C	-1.35245000	2.30888300	3.93838100
H	-1.84273200	2.36522100	1.84243300
C	-0.35566900	2.08949300	4.89202800
H	1.70979400	1.55752400	5.20716900
H	-2.34776800	2.61151200	4.25127400
H	-0.57176200	2.21504200	5.94864400
Cu	1.23239000	-0.24690800	0.14920500
C	3.82666100	-3.01002100	2.46760200
C	4.17280900	-3.93324600	1.50065300
C	3.65129200	-3.80268600	0.19384000
C	2.78610400	-2.70807400	-0.05499700
C	2.95909700	-1.95659900	2.12527500
C	3.96012600	-4.71529700	-0.87151400
C	2.23245900	-2.52849500	-1.37811500
C	2.55986500	-3.44511700	-2.40761300
C	3.43662300	-4.54493400	-2.11837900
C	1.99661800	-3.21130200	-3.68316600
H	2.22366200	-3.88927400	-4.50107500
C	1.16982500	-2.12119900	-3.87627600
C	0.90214200	-1.26485900	-2.79094600
H	4.62544300	-5.54779800	-0.66293500
H	4.21203800	-3.08137300	3.47872100
H	4.84215600	-4.75713600	1.73123300
H	2.66753000	-1.21438100	2.86232900
H	3.67949200	-5.23956500	-2.91700200
H	0.73026500	-1.91343800	-4.84568800
H	0.26632700	-0.38975200	-2.89894600
N	2.45133900	-1.80624400	0.90537900
N	1.41670400	-1.46586700	-1.58171300
O	-0.51948100	1.75594900	-1.46335100
N	1.12544100	3.47292700	-1.09300700
C	1.65076100	2.89526100	0.02660500
H	1.66022400	3.51494400	0.93117900
C	2.98599700	2.23250800	-0.21077600
C	3.97547500	2.25157900	0.78134500
C	3.26872300	1.61019000	-1.43614300
C	5.21297600	1.64010700	0.56711600
H	3.78626200	2.77054800	1.71788200
C	4.50482800	0.99874100	-1.64985800
H	2.52334900	1.65518800	-2.22317400
C	5.47959700	1.00458100	-0.64735500
H	5.97557300	1.68043200	1.33986700
H	4.71830500	0.53689300	-2.61030900
H	6.44734500	0.54346800	-0.82213300
C	0.20885600	4.50612600	-1.00436200
C	-0.38231600	5.00726800	0.18203200
C	-0.13501500	5.12347300	-2.23035800
C	-1.26584300	6.07994900	0.13003700
H	-0.15296700	4.56086100	1.14375500
C	-1.01823500	6.19466500	-2.27338100
H	0.32230200	4.73094200	-3.13275400
C	-1.58912000	6.68036300	-1.09224400
H	-1.70497600	6.45684300	1.04956000
H	-1.26046000	6.65809300	-3.22539300
H	-2.27633000	7.52056300	-1.12133400

J

Cu	-1.25566800	0.11599200	-0.64376300
C	-5.15894400	1.08908900	1.41125400
C	-5.74831300	-0.15878200	1.44320900
C	-5.05146000	-1.27222300	0.92017500
C	-3.76183800	-1.03594300	0.38224600
C	-3.86887300	1.22266800	0.86184500
C	-5.58963200	-2.60315200	0.91061300
C	-3.01360900	-2.13794300	-0.18021500
C	-3.57563000	-3.43949400	-0.17402900
C	-4.88099800	-3.64307600	0.38800500
C	-2.80773900	-4.48313900	-0.73742100
H	-3.20579400	-5.49369300	-0.75147400
C	-1.56288200	-4.20503100	-1.26651800
C	-1.08906300	-2.88195100	-1.23261000
H	-6.57861700	-2.76559700	1.32861800
H	-5.66907700	1.96330200	1.80056600
H	-6.74087400	-0.29646100	1.86262900
H	-3.37272600	2.18854700	0.82885100
H	-5.29582800	-4.64649000	0.38371300
H	-0.95092300	-4.98330100	-1.70866000
H	-0.11729400	-2.63023500	-1.64570500
N	-3.18896700	0.19410200	0.36525600
N	-1.78709800	-1.87649600	-0.70794900
C	0.20159000	1.48028200	-2.38624300
C	-0.16220400	1.72467500	-1.13453900
C	-0.63023900	3.04690900	-0.63958000
C	-0.82794000	3.25764300	0.73708400
C	-0.89372000	4.11955600	-1.51258500
C	-1.26876700	4.48929400	1.22388700
H	-0.62708400	2.44140200	1.42654800
C	-1.33177200	5.34948900	-1.02517900
H	-0.75347600	3.98855000	-2.58252600
C	-1.52350300	5.54352200	0.34515900
H	-1.40608700	4.62630300	2.29307000
H	-1.52446100	6.16062800	-1.72138000
H	-1.86250200	6.50347100	0.72161500
Cu	1.33275100	0.70312800	-0.23600200
C	2.81421200	0.09415800	3.85144700
C	4.02793600	-0.52353000	3.62368200
C	4.43834300	-0.78778300	2.29763200
C	3.56258000	-0.39885600	1.25276100
C	2.01318300	0.44729100	2.75127000
C	5.68857100	-1.41638500	1.97569100
C	3.94273800	-0.64264300	-0.12100700
C	5.18908800	-1.25565300	-0.40251100
C	6.04961600	-1.63911300	0.68076600
C	5.52027600	-1.45381300	-1.76272500
H	6.46751000	-1.91858700	-2.02117400
C	4.63882300	-1.04836100	-2.74503000
C	3.42083000	-0.45125400	-2.36657800
H	6.34611000	-1.70708000	2.78938700
H	2.47240100	0.31382400	4.85672000
H	4.67307800	-0.80590400	4.45066500
H	1.05699000	0.93937600	2.89894700
H	6.99996800	-2.10959100	0.44700300
H	4.86766700	-1.17983600	-3.79686700
H	2.70743800	-0.11579600	-3.11426100
N	2.36945500	0.21117800	1.49054700

N	3.07981500	-0.25892700	-1.09547000
O	0.55022200	1.19561900	-3.47744100

K

O	-1.64969000	-3.25224600	-0.02792300
Cu	-0.48079900	-0.11676800	-0.00443600
N	1.41178800	-1.31951400	0.00556400
C	2.48773200	-0.49789700	0.00449100
C	3.82162100	-0.97761200	0.01213500
C	4.00117200	-2.37956000	0.02165600
C	2.89535400	-3.20683600	0.02291500
C	1.60720900	-2.63328700	0.01441800
C	2.24105700	0.92645800	-0.00524700
C	3.33805700	1.82527600	-0.00818200
C	4.67615000	1.30352000	-0.00038600
C	4.90814300	-0.03946400	0.00952300
C	3.04867600	3.20744500	-0.01871600
C	1.73208800	3.62574800	-0.02570900
C	0.70930900	2.66175300	-0.02150800
N	0.94741600	1.35092900	-0.01135200
C	-2.01687000	-2.11475400	-0.01709800
C	-2.29495200	-0.84312100	-0.00282800
C	-3.59235800	-0.17581500	0.00382700
C	-4.80740900	-0.89737400	-0.03973900
C	-6.03858800	-0.24841600	-0.03241000
C	-6.10866200	1.14692600	0.01860300
C	-4.92068300	1.87968900	0.06231000
C	-3.68829200	1.22986100	0.05504200
H	0.71127400	-3.24921700	0.01424700
H	3.00072300	-4.28644500	0.03000200
H	5.00714400	-2.79045900	0.02772900
H	-0.33438500	2.95979200	-0.02667800
H	1.47435100	4.67917000	-0.03420300
H	3.86336900	3.92623000	-0.02127400
H	5.50421200	2.00652500	-0.00253800
H	5.92470400	-0.42267100	0.01547600
H	-2.76856900	1.80865400	0.09167300
H	-4.95365100	2.96602400	0.10296600
H	-7.07032000	1.65199300	0.02409800
H	-6.95206100	-0.83728700	-0.06707700
H	-4.77304800	-1.98320900	-0.08045800

L

C	-1.70922600	2.46961300	-0.71721100
C	-2.89094900	1.91552600	-0.18770700
C	-3.95408900	2.77565400	0.14816800
C	-3.82624400	4.15358300	-0.00337800
C	-2.64133300	4.69095200	-0.51135800
C	-1.58664200	3.84603500	-0.87431500
C	-3.08663200	0.48672900	0.02344500
N	-2.15610500	-0.41460900	0.07575800
C	-2.55168900	-1.78565000	0.20535300
C	-1.83741900	-2.61337900	1.08290500
C	-2.19935000	-3.95113600	1.22873600
C	-3.26124300	-4.47713900	0.48867900
C	-3.95993100	-3.65810500	-0.39998000
C	-3.60999700	-2.31578600	-0.54641800
H	-4.12112400	0.16695800	0.17539100
H	-1.02235500	-2.19508400	1.66757500
H	-1.65232400	-4.58247700	1.92195300
H	-3.53584900	-5.52141400	0.59738500
H	-4.77429400	-4.06542200	-0.99098800
H	-4.13139600	-1.69093000	-1.26513000
H	-4.87703300	2.35662900	0.54015100
H	-4.64948800	4.80673700	0.26741400
H	-2.54312800	5.76462400	-0.63843600
H	-0.67657400	4.26367500	-1.29364700
H	-0.90357300	1.81778800	-1.04722000
Cu	-0.20137100	-0.16193600	0.01757200
C	2.53310000	0.76753700	3.36934800
C	3.76015900	0.66099500	2.74509500
C	3.81890800	0.33329900	1.37110200
C	2.58915300	0.12747800	0.69801300
C	1.36482700	0.54444900	2.61830700
C	5.05156400	0.20261700	0.64655400
C	2.59416100	-0.20846400	-0.70801300
C	3.82907600	-0.33054700	-1.39192600
C	5.05641500	-0.11598200	-0.67840100
C	3.78056400	-0.66332200	-2.76507400
H	4.70590600	-0.76665000	-3.32433700
C	2.55814700	-0.85427200	-3.37811600
C	1.38409000	-0.71058700	-2.61671400
H	5.98450600	0.36296500	1.17828900
H	2.45499100	1.01779300	4.42143500
H	4.68134100	0.82585700	3.29646100
H	0.38592900	0.62064300	3.08208600
H	5.99334000	-0.21363500	-1.21825400
H	2.48792400	-1.11204300	-4.42891700
H	0.40877200	-0.85554700	-3.07146200
N	1.38714300	0.23361200	1.32439400
N	1.39634200	-0.39684900	-1.32328100

$TS_{K\text{-}M}$

O	-1.43681300	-1.96623000	-2.50745700
Cu	0.27995100	-0.09164900	-0.46655100
N	2.07270000	-1.15693900	-0.72409600
C	3.14407500	-0.46409300	-0.25593200
C	4.46331500	-0.97851400	-0.30609500
C	4.63800800	-2.26174100	-0.87323000
C	3.54247600	-2.95035300	-1.35566800
C	2.26991300	-2.35785000	-1.26252300
C	2.91256300	0.85326700	0.29306600
C	4.01057600	1.61287600	0.76778000
C	5.33242200	1.05476900	0.71518600
C	5.54968100	-0.18838600	0.20089500
C	3.73683600	2.90632700	1.26831100
C	2.43869900	3.37676200	1.26894400
C	1.41235000	2.54786200	0.77986300
N	1.63655900	1.32205100	0.31382400
C	-1.55893300	-1.07068800	-1.75407000
C	-1.72892700	-0.17862100	-0.78025800
C	-2.35294800	1.16570800	-0.98714800
C	-2.94443900	1.53837800	-2.20767900
C	-3.53158100	2.79329100	-2.36443000
C	-3.55190800	3.70400200	-1.30580200
C	-2.97590000	3.34563100	-0.08621300
C	-2.37978300	2.09327200	0.06993800
H	1.38954400	-2.86841000	-1.64174500

H	3.64595900	-3.93180700	-1.80476400
H	5.63414300	-2.69103200	-0.93031700
H	0.38207800	2.89062000	0.75876900
H	2.19865900	4.36834600	1.63620400
H	4.55184600	3.52020400	1.64081400
H	6.16069900	1.64857800	1.08960800
H	6.55347500	-0.60017700	0.15979200
H	-1.92948200	1.82918100	1.02258900
H	-2.99032000	4.04160700	0.74788300
H	-4.01329600	4.67853100	-1.42964900
H	-3.97824200	3.05759300	-3.31834600
H	-2.94543800	0.84094600	-3.04153400
N	-2.80433800	-1.54642300	1.35158900
C	-1.96710400	-2.75137700	1.64352100
H	-2.29241300	-3.17115400	2.60599200
C	-2.85434900	-0.64303900	2.54231900
H	-3.45843600	-1.12690700	3.32345400
C	-4.18113400	-1.93623500	0.91054300
H	-4.60115100	-2.62706600	1.65557600
H	-4.06348300	-2.49486100	-0.02022100
C	-5.14097500	-0.77148500	0.67462600
H	-4.72146000	-0.02649400	-0.00643900
H	-6.05257700	-1.16610200	0.21694600
H	-5.43544100	-0.27187600	1.60048700
C	-1.48732300	-0.25563400	3.10738400
H	-1.62698500	0.53483400	3.85057600
H	-0.98491400	-1.08621200	3.60881100
H	-0.82045200	0.12919000	2.32898000
H	-3.38362100	0.25877900	2.23177500
H	-0.94257300	-2.39429600	1.77611300
C	-1.98223800	-3.84791000	0.57828000
H	-1.73146300	-3.47394800	-0.41787400
H	-1.23172300	-4.59627500	0.84961100
H	-2.94281700	-4.36410600	0.51867200
H	-2.20887800	-0.86817800	0.29899800

M

O	2.01891300	-3.65025300	-0.16106100
Cu	0.39107000	-0.80917200	-0.47809700
N	-0.44234300	1.11872400	-0.37490600
C	-1.77235000	1.03950000	-0.10459200
C	-2.59358500	2.18836900	0.00780800
C	-1.97463700	3.44765600	-0.16784500
C	-0.62118700	3.51321200	-0.43346200
C	0.11360400	2.31726900	-0.52937900
C	-2.35391700	-0.27170200	0.07820100
C	-3.73764500	-0.38767700	0.35977100
C	-4.54137000	0.79786100	0.45893200
C	-3.99239600	2.03373700	0.29135000
C	-4.25954400	-1.68999300	0.53355100
C	-3.42176600	-2.78263400	0.42833900
C	-2.06061700	-2.57205100	0.14766100
N	-1.53950200	-1.35814100	-0.02358000
C	2.16121600	-2.55531400	-0.51794300
C	2.35176400	-1.27794100	-0.93828200
C	3.21485200	-0.31206800	-0.18088900
C	3.80820100	0.74587500	-0.88514800
C	4.61245300	1.66898900	-0.21519800
C	4.83262700	1.54647500	1.15782100
C	4.24255400	0.49342200	1.86082400
C	3.43322200	-0.42924200	1.20011400
H	1.18066200	2.33313200	-0.73142600
H	-0.11869400	4.46438500	-0.56879800
H	-2.57048400	4.35242600	-0.08962700
H	-1.37718300	-3.41119800	0.06200200
H	-3.79222700	-3.79318300	0.55829700
H	-5.31608200	-1.81882600	0.74994100
H	-5.59999200	0.68925900	0.67365400
H	-4.60694900	2.92519300	0.37083400
H	2.97939000	-1.24200300	1.76267300
H	4.41226200	0.38735200	2.92774000
H	5.46281700	2.26215900	1.67578100
H	5.07519500	2.47785100	-0.77241200
H	3.65553500	0.83781000	-1.95731400
H	2.34744900	-1.20288200	-2.02844500

N

O	-0.34335300	0.15212300	3.32060200
Cu	0.60403100	-0.13278700	0.04695000
N	2.34983800	0.90994200	-0.63187700
C	3.48150900	0.26090100	-0.24930900
C	4.77622800	0.74489900	-0.56819900
C	4.85849200	1.94834300	-1.30536200
C	3.70056900	2.60175900	-1.67646000
C	2.45962600	2.04562000	-1.31509100
C	3.34914100	-0.96157100	0.51541000
C	4.51675400	-1.65504800	0.92525400
C	5.80977700	-1.13818600	0.57724900
C	5.93446100	0.01396800	-0.13845600
C	4.34324100	-2.84209600	1.67244600
C	3.06871200	-3.27841600	1.97326300
C	1.96949100	-2.52414800	1.52534400
N	2.09909900	-1.40306800	0.82095200
C	-0.63568600	0.55801600	2.27017100
C	-0.97579600	1.00182500	1.04777700
C	-0.77377700	2.41391400	0.61185900
C	0.08112200	3.30045300	1.28676200
C	0.23840100	4.60906500	0.83417600
C	-0.44460200	5.05268000	-0.30165100
C	-1.29526100	4.17584200	-0.97765400
C	-1.46231000	2.86577800	-0.52577300
H	1.53110900	2.54032300	-1.58423000
H	3.73091900	3.52928100	-2.23739700
H	5.83341900	2.34836100	-1.56913500
H	0.95546500	-2.84308100	1.74615100
H	2.90189000	-4.18358100	2.54663900
H	5.21552300	-3.39797700	2.00408700
H	6.69018300	-1.68552800	0.90009500
H	6.91609700	0.40073400	-0.39527200
H	-2.14107300	2.19414500	-1.04428400
H	-1.84125500	4.51247900	-1.85399600
H	-0.32228000	6.07359200	-0.64927100
H	0.89426800	5.28547300	1.37406900
H	0.62040400	2.97252400	2.17235400
H	-1.80125200	0.42760300	0.59689300
C	-5.20244000	0.32226900	0.48789500
C	-5.20607600	-0.89003100	-0.22789600
C	-6.42352300	-1.56341200	-0.42992700

C	-7.61252100	-1.04283100	0.07567800
C	-7.59690600	0.15925500	0.78532100
C	-6.39035100	0.84074600	0.98872500
C	-3.98808700	-1.48223100	-0.77394700
N	-2.81256000	-0.97260800	-0.64672300
C	-1.69803100	-1.65820200	-1.15744200
C	-0.70518800	-0.90812400	-1.83412900
C	0.40942400	-1.55639200	-2.38776400
C	0.57821400	-2.93445600	-2.22986200
C	-0.37858000	-3.66707900	-1.52395400
C	-1.50387700	-3.04018700	-0.98528200
H	-4.12972400	-2.41587100	-1.33527900
H	-0.89966700	0.13889700	-2.05343000
H	1.12985100	-0.97978000	-2.95944900
H	1.44122700	-3.43210500	-2.65998500
H	-0.25421600	-4.73850100	-1.39584100
H	-2.23633100	-3.61642000	-0.42808700
H	-6.43271300	-2.49818300	-0.98465700
H	-8.54740900	-1.57050600	-0.08387100
H	-8.52232300	0.56846600	1.17916600
H	-6.38330700	1.77698200	1.53841500
H	-4.26642500	0.84912300	0.63844600

TS_{N-O}

O	-1.93141300	0.00284200	1.69804600
Cu	0.63611000	0.34822600	-0.24484800
N	2.52405700	0.68897900	-1.09057900
C	3.51081700	0.13569000	-0.33537600
C	4.88219100	0.25124100	-0.67528600
C	5.20372600	0.97682200	-1.84494000
C	4.19280300	1.53905500	-2.59864800
C	2.85993600	1.37071200	-2.18237700
C	3.13422500	-0.58833200	0.85956500
C	4.14018000	-1.17355100	1.66785600
C	5.51853100	-1.04140100	1.28880100
C	5.87426300	-0.35737200	0.16539600
C	3.72046400	-1.86379800	2.82816200
C	2.37500100	-1.94012600	3.12837800
C	1.44795600	-1.32587200	2.26596400
N	1.81385800	-0.67234100	1.16714900
C	-1.72848400	0.35677500	0.59361600
C	-1.24351700	1.25071900	-0.34065700
C	-0.90410000	2.64872500	0.07443400
C	-0.68491800	3.02581700	1.41074300
C	-0.36037800	4.34447600	1.72760900
C	-0.24605200	5.30881800	0.72437400
C	-0.46590800	4.94326900	-0.60526200
C	-0.79134400	3.62724600	-0.92888700
H	2.04435000	1.80653000	-2.75136800
H	4.40717200	2.10540400	-3.49807100
H	6.24390300	1.08764800	-2.13751200
H	0.38280100	-1.35958100	2.47504100
H	2.02322800	-2.45868300	4.01334600
H	4.46217400	-2.32480200	3.47407500
H	6.27578100	-1.49666200	1.91991500
H	6.91920000	-0.25881200	-0.11254700
H	-0.96962500	3.35468200	-1.96640800
H	-0.39136800	5.68564000	-1.39454400
H	0.00445200	6.33437200	0.97629300
H	-0.20094400	4.61861500	2.76624800
H	-0.78007600	2.29240200	2.20591200
H	-1.58945700	1.10285600	-1.35631900
C	-4.88468500	0.46791600	-0.13925900
C	-4.84483300	-0.93530600	-0.25551300
C	-6.03193400	-1.67056000	-0.06998700
C	-7.22239300	-1.02626700	0.25335300
C	-7.24560100	0.36431600	0.37777000
C	-6.07751300	1.10831900	0.17459400
C	-3.64329000	-1.69431100	-0.57947200
N	-2.42625700	-1.26367400	-0.60757400
C	-1.38412700	-2.18778800	-0.90110100
C	-0.47997300	-1.89635100	-1.93525800
C	0.55445500	-2.78744200	-2.22560600
C	0.71052800	-3.95723200	-1.47732700
C	-0.18023300	-4.23868400	-0.43975300
C	-1.22561100	-3.36028900	-0.14885600
H	-3.81875600	-2.74738200	-0.82831200
H	-0.62961200	-1.00886600	-2.54168400
H	1.23025600	-2.57250900	-3.04788800
H	1.51521000	-4.64830800	-1.70758900
H	-0.06635700	-5.14653300	0.14503700
H	-1.91118900	-3.56876600	0.66701300
H	-6.01286100	-2.75229000	-0.17320300
H	-8.12892700	-1.60391600	0.40208200
H	-8.17378600	0.87113300	0.62354800
H	-6.10256500	2.19034400	0.25649100
H	-3.99160800	1.05406100	-0.31861600

O

O	-1.59880800	0.06679600	1.84040000
Cu	0.65590700	0.22643400	-0.49133700
N	2.47304900	0.03443800	-1.45362900
C	3.53449500	0.08031200	-0.60243900
C	4.87524500	0.01167600	-1.05818200
C	5.08538500	-0.10899300	-2.45034400
C	3.99998400	-0.15444500	-3.30256900
C	2.70521700	-0.07862300	-2.75973600
C	3.27236600	0.20749600	0.81515500
C	4.35803000	0.26579800	1.72424500
C	5.70253900	0.19324600	1.22638200
C	5.95021000	0.07054600	-0.10787400
C	4.04541900	0.39630500	3.09715400
C	2.72417400	0.45980200	3.49340700
C	1.71286000	0.39192800	2.51536200
N	1.97968100	0.26859700	1.21910200
C	-1.67892600	-0.10479300	0.62698300
C	-1.36842100	0.66967200	-0.50907600
C	-1.16583700	2.14344600	-0.45833100
C	-1.02314800	2.87370700	0.73971000
C	-0.85715700	4.25895300	0.71016400
C	-0.82255200	4.94957500	-0.50201500
C	-0.96230300	4.23798800	-1.69677100
C	-1.13004700	2.85635500	-1.67552000
H	1.83283300	-0.10809200	-3.40505900
H	4.12767900	-0.24409100	-4.37558900
H	6.09932200	-0.16258400	-2.83633200
H	0.66027800	0.43555500	2.78214700
H	2.45445800	0.56224400	4.53884400

H	4.84906000	0.44685500	3.82639200
H	6.52240300	0.23999500	1.93693100
H	6.97001100	0.01808200	-0.47705200
H	-1.24731000	2.31472100	-2.61149500
H	-0.94588700	4.76165100	-2.64834000
H	-0.69243600	6.02720000	-0.51740400
H	-0.75739600	4.80162900	1.64612300
H	-1.05833500	2.35050300	1.68776000
H	-1.75131700	0.28607300	-1.45195500
C	-4.63315600	0.51932400	0.64985600
C	-4.65942700	-0.78237000	0.09625100
C	-5.87126100	-1.27514600	-0.44345300
C	-7.00974900	-0.48070800	-0.47437600
C	-6.96414900	0.81071800	0.05801000
C	-5.78191600	1.29994000	0.62527100
C	-3.54696600	-1.70403800	0.04320300
N	-2.27035800	-1.49933600	0.21501800
C	-1.33657900	-2.59064700	0.09031500
C	-1.35760600	-3.39643400	-1.05306300
C	-0.45694300	-4.45686600	-1.15572600
C	0.45597600	-4.70475200	-0.12886700
C	0.47087700	-3.88798900	1.00527700
C	-0.42241100	-2.82429300	1.12360100
H	-3.80800100	-2.73513600	-0.19355100
H	-2.05019700	-3.18149800	-1.86119800
H	-0.46725600	-5.08345900	-2.04185900
H	1.15206600	-5.53343700	-0.21072400
H	1.17118700	-4.08753200	1.81023200
H	-0.43993700	-2.19449400	2.00520300
H	-5.90584600	-2.28226700	-0.85002000
H	-7.92957300	-0.86573300	-0.90176900
H	-7.85407600	1.43255900	0.04460600
H	-5.75969700	2.29414800	1.05914700
H	-3.74481500	0.90012800	1.13506500

TS_{O-P}

O	0.75009900	0.72355800	-0.72902000
Cu	-1.03478400	0.28347900	-0.17423500
N	-2.63521300	-0.75548800	0.48821600
C	-3.77695100	-0.02025500	0.59355700
C	-4.99619300	-0.58023800	1.05117800
C	-4.99532900	-1.94950600	1.40046000
C	-3.83179600	-2.68329000	1.28216600
C	-2.66788800	-2.04616000	0.81900700
C	-3.73264600	1.37681600	0.21883400
C	-4.90877700	2.16192400	0.31582500
C	-6.12470200	1.56245300	0.78749600
C	-6.16632600	0.24710300	1.14010500
C	-4.81409100	3.51942400	-0.06822500
C	-3.60708500	4.01948700	-0.51456800
C	-2.49415700	3.15859000	-0.57422100
N	-2.55243600	1.88039000	-0.21952900
C	1.78305000	0.03908600	-0.99189600
C	3.08696000	0.53017900	-1.30151800
C	3.63620900	1.81199000	-0.94693400
C	3.03031700	2.66762100	0.00477000
C	3.63403200	3.86955900	0.35440400
C	4.84792500	4.24906000	-0.22846300
C	5.46190500	3.41524700	-1.16932800
C	4.86509900	2.21240700	-1.52336600
H	-1.73991300	-2.59750900	0.70740000
H	-3.79990500	-3.73605700	1.53947600
H	-5.91149700	-2.41158400	1.75687100
H	-1.52980000	3.51961000	-0.92135400
H	-3.50366200	5.05549500	-0.81790600
H	-5.69302800	4.15494300	-0.00928400
H	-7.01512100	2.18004800	0.85679700
H	-7.08986000	-0.20023900	1.49485600
H	5.34502700	1.56297700	-2.25083800
H	6.40385400	3.70717200	-1.62315900
H	5.31259000	5.19071100	0.04768000
H	3.15934700	4.51801800	1.08479900
H	2.08354300	2.38153200	0.44682800
H	3.64231800	-0.06241600	-2.02141700
C	4.85319800	-1.95818400	1.25831100
C	3.56935500	-1.48031900	0.91591000
C	2.79625700	-0.84361200	1.91445100
C	3.29886400	-0.69520000	3.20030900
C	4.57700000	-1.16788000	3.52392900
C	5.35035000	-1.80034200	2.54772000
C	3.09917400	-1.71173600	-0.43355000
N	1.84877600	-1.35653700	-0.91939500
C	0.89595600	-2.28922300	-1.40436100
C	0.05282800	-1.95459800	-2.47564600
C	-0.84944100	-2.89960200	-2.95779000
C	-0.90601300	-4.17955300	-2.39565800
C	-0.06012900	-4.50950100	-1.33563300
C	0.83283700	-3.56425000	-0.82642700
H	3.61902500	-2.48382700	-1.00322200
H	0.12405500	-0.97473100	-2.93605800
H	-1.49535900	-2.64329800	-3.79189700
H	-1.60080500	-4.91533200	-2.78795000
H	-0.09533800	-5.50204300	-0.89705500
H	1.47449900	-3.80623100	0.01516400
H	1.80088600	-0.48153900	1.67729300
H	2.69190300	-0.21360600	3.96107400
H	4.96176300	-1.04886000	4.53183700
H	6.33978800	-2.17338300	2.79291400
H	5.45698500	-2.45274600	0.50199800

P

O	0.27419400	0.97747800	-1.04302200
Cu	-1.43850300	0.30470600	-0.41606800
N	-2.78553900	-0.87450000	0.53106800
C	-4.02686700	-0.32152800	0.61967500
C	-5.10010900	-0.97739000	1.27328500
C	-4.84161400	-2.24601400	1.83983000
C	-3.57946200	-2.79617000	1.73606700
C	-2.57404100	-2.07523000	1.06894500
C	-4.24646400	0.97749600	0.02015100
C	-5.53027700	1.57282600	0.09932400
C	-6.59287600	0.88096200	0.77219500
C	-6.38639400	-0.34252100	1.33499800
C	-5.69412700	2.84169100	-0.50298000
C	-4.62198500	3.44191300	-1.13248300
C	-3.38514000	2.76965300	-1.15893900
N	-3.20124600	1.57728800	-0.60313100
C	1.42138500	0.50407200	-1.09672400

C	2.79767100	1.11797100	-1.30881900
C	3.30394200	2.19310500	-0.38152200
C	2.56427400	2.63688300	0.72159500
C	3.07396600	3.62926700	1.56165500
C	4.32711300	4.18709700	1.30979300
C	5.06860800	3.75393800	0.20787700
C	4.55791100	2.76778200	-0.63369600
H	-1.57450400	-2.48377400	0.96308300
H	-3.35099600	-3.76844700	2.15806000
H	-5.64002800	-2.77690200	2.35038400
H	-2.52142000	3.21473600	-1.64509700
H	-4.71609600	4.41432000	-1.60316700
H	-6.66274100	3.33190800	-0.46424100
H	-7.56915800	1.35352700	0.82361300
H	-7.19498100	-0.86039600	1.84178700
H	5.13852900	2.43952200	-1.49209900
H	6.04212000	4.18781100	0.00098200
H	4.72239000	4.95903600	1.96295500
H	2.48816400	3.96749000	2.41125800
H	1.57932400	2.22398500	0.91792600
H	2.89125400	1.44885200	-2.35060800
C	5.54733800	-1.22573600	-0.42145200
C	4.26857900	-0.76476300	-0.08460000
C	3.92346500	-0.64696000	1.27002800
C	4.84134400	-0.98483900	2.26302200
C	6.11723200	-1.43808600	1.91703800
C	6.46949300	-1.55661300	0.57260700
C	3.32164100	-0.38653500	-1.19067100
N	1.88293100	-0.76346700	-1.02435800
C	1.22650800	-2.02412400	-1.06004500
C	0.11778200	-2.20236000	-1.90088700
C	-0.51940800	-3.44312100	-1.94827100
C	-0.04677900	-4.50734700	-1.17648300
C	1.06583200	-4.32590500	-0.35095100
C	1.70303600	-3.08636800	-0.28252500
H	3.69897300	-0.75827700	-2.14844100
H	-0.21941800	-1.38741700	-2.53283500
H	-1.37115400	-3.58170600	-2.60723200
H	-0.53450400	-5.47582300	-1.22833300
H	1.44315300	-5.15215800	0.24355200
H	2.56152700	-2.94086100	0.36252100
H	2.93647800	-0.29240100	1.55031900
H	4.56275500	-0.89097600	3.30832000
H	6.83052100	-1.69960100	2.69256200
H	7.45677900	-1.91309700	0.29559800
H	5.82359000	-1.32914400	-1.46799900

TS$_{U1}$

O	0.13825200	-0.74452400	1.68998600
C	0.38307300	-0.16938500	0.65501900
C	1.34525700	0.02714200	-0.28999200
C	2.67913200	-0.55800400	-0.21089800
C	3.09920400	-1.39664400	0.84564300
C	4.38399800	-1.93346200	0.86239000
C	5.29151500	-1.65736800	-0.16425400
C	4.89169300	-0.82880900	-1.21554600
C	3.60892300	-0.28789200	-1.23814400
H	3.31088500	0.35593600	-2.06270500
H	5.58330500	-0.60092000	-2.02293800
H	6.29239100	-2.07897600	-0.14444700
H	4.68069200	-2.57472700	1.68885300
H	2.40646400	-1.61786800	1.65049900
H	1.12277000	0.67786400	-1.12525800
C	-1.66977300	-2.18252300	-0.37659000
C	-2.58913800	-1.15307800	-0.08932300
C	-3.95058900	-1.47817000	0.08043100
C	-4.38198000	-2.79740500	-0.00177000
C	-3.45968700	-3.81057500	-0.27679000
C	-2.11101900	-3.49774500	-0.47287300
C	-2.23730800	0.25487000	0.00584600
N	-1.05421400	0.77500700	0.10592000
C	-0.90166200	2.18949100	0.05469000
C	-0.08233800	2.82597200	0.99802400
C	0.06602400	4.20978600	0.95518900
C	-0.57803300	4.96291600	-0.03086500
C	-1.37697000	4.32469300	-0.98027500
C	-1.54044900	2.93978400	-0.94260800
H	-3.08207500	0.94855500	-0.00853500
H	0.40863900	2.23563800	1.76300300
H	0.69097500	4.70159000	1.69429700
H	-0.44829000	6.04036600	-0.06439000
H	-1.86453900	4.90066500	-1.76112000
H	-2.13060800	2.43479900	-1.70130600
H	-4.66597900	-0.68590000	0.28614900
H	-5.43118400	-3.03599100	0.14212000
H	-3.79240700	-4.84224400	-0.34543700
H	-1.39847900	-4.28455300	-0.70005700
H	-0.62830800	-1.94465200	-0.55386900

Q

O	-0.07236200	-0.59283600	1.82147800
C	-0.08464700	-0.45208900	0.59833900
C	-0.98307900	-0.61479600	-0.43289400
C	-2.33253600	-1.13231100	-0.27665800
C	-2.88024800	-1.50811300	0.97238300
C	-4.18008500	-1.99947000	1.06013300
C	-4.97856200	-2.13120200	-0.07990200
C	-4.45329900	-1.76230300	-1.32135300
C	-3.15391000	-1.27360400	-1.41793900
H	-2.75648800	-0.99153800	-2.39072900
H	-5.05936000	-1.85697300	-2.21912000
H	-5.99242400	-2.51364400	-0.00245700
H	-4.57598000	-2.28247700	2.03258700
H	-2.26688000	-1.40843500	1.86050600
H	-0.66597000	-0.34483200	-1.43381300
C	0.94464400	3.51363500	-1.04020300
C	0.71164900	2.41898900	-0.18267500
C	-0.22304100	2.55477100	0.86843400
C	-0.91321200	3.74989200	1.02378000
C	-0.69798600	4.81972300	0.14580800
C	0.23364300	4.70033000	-0.88650900
C	1.53869600	1.24045200	-0.39105300
N	1.31994200	0.01512700	-0.00388100
C	2.36406400	-0.96119400	-0.04127800
C	2.05848700	-2.27142600	-0.42868900
C	3.07417600	-3.22243700	-0.47815800
C	4.38670400	-2.87714900	-0.14440400
C	4.68203600	-1.57261900	0.25478200

C	3.67285400	-0.61263000	0.31550000
H	2.47261300	1.40683500	-0.93088800
H	1.03602300	-2.51901900	-0.69206100
H	2.83876200	-4.23756900	-0.78262600
H	5.17307900	-3.62475200	-0.18359300
H	5.69464200	-1.30382900	0.53983100
H	3.88809000	0.38770600	0.67795400
H	-0.38061800	1.73720600	1.56335500
H	-1.62452200	3.85115700	1.83748500
H	-1.24827800	5.74692400	0.27590800
H	0.41120200	5.52993900	-1.56380100
H	1.67732800	3.42141100	-1.83784800

TS$_{U2}$

O	0.26914000	-1.43289200	1.58449100
C	0.25840100	-0.98468000	0.43924500
C	-0.75227500	-1.06624800	-0.58067000
C	-2.12736700	-1.42864400	-0.36860200
C	-2.68782200	-1.59085800	0.92351300
C	-4.03929000	-1.88100700	1.07343300
C	-4.86897900	-2.01820500	-0.04442200
C	-4.33204900	-1.86644100	-1.32762600
C	-2.98260400	-1.57997900	-1.48764200
H	-2.56895600	-1.45857500	-2.48583100
H	-4.96929100	-1.97519100	-2.20078700
H	-5.92307900	-2.24795000	0.08209300
H	-4.45159700	-2.00703700	2.07083800
H	-2.03680600	-1.51015600	1.78552600
H	-0.40082900	-1.04151600	-1.60625700
C	-0.56000300	2.89647100	-1.16817600
C	-0.06852200	1.90983500	-0.28598700
C	-0.38228000	2.01893100	1.08972900
C	-1.16947600	3.06796000	1.54584800
C	-1.66056100	4.03321700	0.65782600
C	-1.34909900	3.94240000	-0.70087800
C	0.78768700	0.87137600	-0.82204800
N	1.32386600	-0.16152400	-0.10188800
C	2.69819200	-0.47663300	-0.07173000
C	3.12367600	-1.77174300	0.26904400
C	4.48111400	-2.07439300	0.25400100
C	5.42675800	-1.10885700	-0.10666600
C	5.00180800	0.17897500	-0.43279200
C	3.64597400	0.50421900	-0.40161200
H	1.25416500	1.07622400	-1.78812300
H	2.39344100	-2.51887300	0.55613400
H	4.80332900	-3.07634700	0.52203700
H	6.48383400	-1.35602800	-0.11805000
H	5.72726100	0.94415100	-0.69338000
H	3.32282000	1.52028300	-0.60395100
H	-0.00359000	1.27914900	1.78715400
H	-1.39825300	3.14090000	2.60516600
H	-2.27253000	4.85161200	1.02492000
H	-1.71998400	4.68956900	-1.39656300
H	-0.31981000	2.82941000	-2.22634700

TS$_{K-R}$

C	-0.68471600	0.80996600	-1.50080000
C	-1.15607900	1.58966600	-0.54298700
C	-1.85595900	2.88169200	-0.72204400
C	-2.50976500	3.48518700	0.36838900
C	-1.86324500	3.56350200	-1.95344100
C	-3.14699900	4.71971400	0.23336100
H	-2.51726100	2.98373100	1.33276300
C	-2.51372400	4.78782300	-2.09092000
H	-1.35122400	3.12839200	-2.80736800
C	-3.15764100	5.37483100	-0.99866700
H	-3.63660600	5.16850900	1.09324400
H	-2.50894400	5.29027700	-3.05385800
H	-3.65646700	6.33295400	-1.10620900
Cu	-2.37755600	0.02443400	0.10572200
C	-6.73577000	0.98872600	0.57407200
C	-7.31233800	-0.23079400	0.28152800
C	-6.48744500	-1.32875700	-0.05081500
C	-5.08539300	-1.11381000	-0.05956600
C	-5.33364000	1.10404800	0.54525500
C	-7.00832400	-2.62643600	-0.37561300
C	-4.20290400	-2.20329600	-0.41540100
C	-4.75281600	-3.47194100	-0.73067100
C	-6.17609600	-3.65545800	-0.69844100
C	-3.85111600	-4.50495900	-1.07192000
H	-4.23497500	-5.49073100	-1.31895900
C	-2.49535700	-4.24662800	-1.08927100
C	-2.04345100	-2.95342300	-0.76768200
H	-8.08443900	-2.77140700	-0.35911200
H	-7.34050200	1.85347100	0.82429600
H	-8.39117400	-0.35696800	0.29845600
H	-4.85100500	2.05317100	0.75808100
H	-6.57803400	-4.63438100	-0.94233300
H	-1.77811100	-5.01786900	-1.34787400
H	-0.98455700	-2.71636400	-0.77444600
N	-4.52968000	0.08766400	0.24771400
N	-2.86663500	-1.96195900	-0.44252900
O	-0.11234700	0.06397200	-2.21873000
C	2.09968900	2.45448600	-0.79634600
C	1.23896800	2.70975100	0.28510000
C	1.28525700	3.96997000	0.89697200
C	2.18931400	4.94157400	0.46417900
C	3.05203200	4.67160300	-0.59874400
C	2.99981700	3.42701000	-1.23209800
C	0.28707100	1.68369400	0.80994000
N	0.78801700	0.44971400	1.09933200
C	-0.02829000	-0.45322400	1.76872600
C	0.42529200	-1.78634600	1.93118600
C	-0.32999700	-2.73940800	2.59852400
C	-1.58586700	-2.41617500	3.13527200
C	-2.05828400	-1.11772800	2.99009400
C	-1.31366600	-0.14464100	2.29939900
H	-0.39697600	2.12018500	1.53947100
H	1.39997400	-2.04937600	1.52473900
H	0.06362000	-3.74605100	2.71148000
H	-2.16725900	-3.16149900	3.66821200
H	-3.01465900	-0.83347500	3.42064900
H	-1.66300400	0.88217400	2.33567700
H	0.61317800	4.18861900	1.72217500
H	2.21545300	5.90969900	0.95526900
H	3.75148000	5.42871000	-0.94022600
H	3.65045600	3.21868400	-2.07661700
H	2.03761400	1.50856200	-1.32608600

Cu	2.60281000	-0.07104400	0.52553900
C	3.77502200	-2.09072100	-3.31465100
C	5.10441800	-2.37387200	-3.07149400
C	5.68408400	-1.99431000	-1.83943900
C	4.84741600	-1.33396900	-0.90503500
C	3.02345900	-1.43101400	-2.32307300
C	7.05837000	-2.24261900	-1.50593400
C	5.39224300	-0.91647800	0.36784500
C	6.75430700	-1.17436300	0.66267400
C	7.57135600	-1.84952800	-0.30614000
C	7.23999200	-0.74177800	1.91742500
H	8.27852900	-0.92064200	2.18099700
C	6.38831400	-0.09605700	2.79161700
C	5.05019400	0.11379000	2.41032500
H	7.68479200	-2.75089000	-2.23295700
H	3.30342900	-2.36445400	-4.25215900
H	5.71183400	-2.88161800	-3.81544900
H	1.97795900	-1.18369500	-2.48484200
H	8.61238300	-2.04005000	-0.06283400
H	6.73040800	0.24916800	3.76098600
H	4.35558200	0.61713400	3.07604300
N	3.54080300	-1.06549400	-1.15384000
N	4.56299800	-0.28276900	1.23759800

R

C	0.64006600	0.77252900	1.03045600
C	0.46264200	1.93031500	0.40345700
C	1.17763900	3.14573900	0.85063300
C	1.08081900	4.34051300	0.11480300
C	1.98058700	3.14725000	2.01007200
C	1.77154900	5.48520100	0.51729000
H	0.45082400	4.38868600	-0.76643200
C	2.66522300	4.29202300	2.40752200
H	2.06059500	2.24594000	2.61340100
C	2.56883400	5.47045900	1.66114200
H	1.67517700	6.39539900	-0.06753300
H	3.26823300	4.26658700	3.31095400
H	3.09730900	6.36528700	1.97478000
Cu	2.68365400	-0.49050400	-1.37030200
C	5.66941700	2.00147300	0.82680400
C	6.45717100	1.02208000	1.39693700
C	6.14368100	-0.33970100	1.18028200
C	5.01772300	-0.62673800	0.36909200
C	4.57085700	1.61815100	0.03413000
C	6.90681300	-1.41958000	1.73964000
C	4.65377300	-2.00461900	0.12062000
C	5.42927500	-3.04584800	0.68926300
C	6.56478700	-2.71744100	1.50391100
C	5.03241600	-4.37430500	0.41487100
H	5.60215200	-5.19994600	0.83173200
C	3.92723800	-4.60637900	-0.37993500
C	3.21949500	-3.50785200	-0.90131100
H	7.76720200	-1.17986000	2.35733800
H	5.87553200	3.05487500	0.97936400
H	7.31362000	1.28292300	2.01228700
H	3.92869900	2.36744100	-0.41751700
H	7.14842100	-3.52768800	1.93051400
H	3.59939700	-5.61433200	-0.60900800
H	2.34819400	-3.64977300	-1.53373900
N	4.25509800	0.34708700	-0.19330400
N	3.56903500	-2.24811700	-0.65949300
O	0.84276400	-0.23491300	1.60265800
C	-2.39236200	2.73575800	0.74435900
C	-1.81421700	2.64258300	-0.52737000
C	-2.47634700	3.23235900	-1.61400300
C	-3.69161100	3.89338500	-1.43614200
C	-4.26409300	3.97580400	-0.16347700
C	-3.61092600	3.39673400	0.92532800
C	-0.51218800	1.88081100	-0.78198200
N	-0.82125800	0.48904100	-1.12804300
C	-0.00375700	-0.17336400	-1.97553900
C	-0.32979000	-1.52206700	-2.34635700
C	0.35627700	-2.21002600	-3.32935300
C	1.46388600	-1.64294300	-3.99190200
C	1.86497600	-0.36593100	-3.61661500
C	1.19655400	0.36245900	-2.58537200
H	-0.02461800	2.38626700	-1.62786200
H	-1.20197300	-1.97206800	-1.87698500
H	0.01993200	-3.20702700	-3.60384300
H	1.98161100	-2.18001200	-4.77891500
H	2.68233400	0.12269400	-4.14195800
H	1.42522700	1.41933400	-2.48638300
H	-2.03248500	3.17371500	-2.60512600
H	-4.18481900	4.35624300	-2.28630100
H	-5.20357900	4.50156300	-0.02062600
H	-4.03985500	3.47215400	1.92052200
H	-1.87485100	2.31253700	1.59982600
Cu	-2.50404100	-0.22671700	-0.42302100
C	-2.76908600	-2.62962600	3.40542200
C	-4.10896000	-2.96297600	3.41326000
C	-4.94433600	-2.51476000	2.36481400
C	-4.34249600	-1.73624200	1.34426800
C	-2.26439500	-1.84943100	2.34670300
C	-6.34863900	-2.80612000	2.29712500
C	-5.15370400	-1.24576100	0.25210900
C	-6.53858800	-1.54504800	0.22112000
C	-7.11322800	-2.34021900	1.26970400
C	-7.28981500	-1.03162200	-0.86031200
H	-8.35430500	-1.24002900	-0.92038100
C	-6.66128500	-0.26900500	-1.82456900
C	-5.27998100	-0.02650200	-1.70969900
H	-6.79264700	-3.40520300	3.08668200
H	-2.10349800	-2.95480900	4.19766800
H	-4.53037900	-3.56216300	4.21558000
H	-1.21633400	-1.56452500	2.31508000
H	-8.17537600	-2.56256300	1.22808700
H	-7.21012800	0.14241100	-2.66448300
H	-4.75649100	0.57292500	-2.44770900
N	-3.02427500	-1.41709800	1.34577700
N	-4.54508000	-0.50196700	-0.70795100

S

C	-1.69162200	0.66580600	1.66713500
C	-2.57675200	0.15694100	0.81608700
C	-3.95047800	-0.14415800	1.27043500
C	-4.85882000	-0.79392600	0.41363500
C	-4.40422200	0.21407600	2.55754000
C	-6.16333400	-1.06789700	0.82759500

H	-4.54320200	-1.10034100	-0.57722800
C	-5.70268200	-0.06886200	2.96688600
H	-3.73236100	0.72646100	3.24201900
C	-6.59567800	-0.71117000	2.10385300
H	-6.84174800	-1.56953300	0.14270600
H	-6.02175900	0.22037800	3.96462500
H	-7.61053700	-0.92803000	2.42354000
O	-0.95110700	1.11659100	2.45721100
C	-1.79512400	-2.52475500	-0.10194500
C	-1.84011500	-1.47538400	-1.02527400
C	-1.64582000	-1.77119900	-2.38449600
C	-1.41403300	-3.07844700	-2.80817900
C	-1.37100800	-4.12179500	-1.87536100
C	-1.56142800	-3.83953100	-0.52252200
C	-2.08335200	-0.01293000	-0.63231300
N	-0.88375400	0.76977600	-0.90910800
C	-1.05324800	2.09899700	-1.25436100
C	0.04825000	2.81224200	-1.80161700
C	-0.04858300	4.14927100	-2.16805400
C	-1.25342700	4.84783300	-2.02556200
C	-2.35262600	4.16438400	-1.50493600
C	-2.26582000	2.82607600	-1.12117400
H	-2.90697100	0.34294000	-1.27476000
H	0.97657000	2.26901600	-1.96436400
H	0.82118200	4.64674700	-2.59272300
H	-1.33202500	5.89018100	-2.31927700
H	-3.30119500	4.68241700	-1.38071400
H	-3.13891200	2.35423900	-0.68459800
H	-1.66788000	-0.95930400	-3.10621900
H	-1.27320100	-3.28703600	-3.86544700
H	-1.20133500	-5.14355300	-2.20448600
H	-1.53883100	-4.64184600	0.21046400
H	-1.95462200	-2.31386800	0.95049300
Cu	0.83717600	0.02674300	-0.55013600
C	3.85877400	3.10878100	1.18437000
C	4.91581400	2.27125200	1.48245300
C	4.82002000	0.89479600	1.17828700
C	3.61598700	0.44789800	0.57505200
C	2.71155700	2.56906700	0.56868200
C	5.86877200	-0.04832400	1.44449500
C	3.45346600	-0.95432700	0.26186200
C	4.51284500	-1.85749900	0.53221700
C	5.72326100	-1.36606300	1.12923400
C	4.31382000	-3.21573700	0.20160500
H	5.10485100	-3.93552200	0.39312900
C	3.11378300	-3.60943000	-0.35816600
C	2.11186300	-2.64923200	-0.57975300
H	6.78612600	0.31093800	1.90256400
H	3.90045700	4.16958800	1.40734100
H	5.81993700	2.65551200	1.94717300
H	1.86961300	3.19984500	0.29446800
H	6.52295000	-2.07317700	1.33063400
H	2.92693800	-4.64438500	-0.62299800
H	1.15318400	-2.93163200	-1.00215700
N	2.59505500	1.28167700	0.27042400
N	2.27059900	-1.35843000	-0.27905800

TS$_{S-T}$

C	-1.78259500	1.12827300	1.18463600
C	-2.65518700	0.25402500	0.64439100
C	-3.89445900	-0.15959500	1.31259700
C	-4.78398800	-1.04381700	0.67036000
C	-4.24745900	0.30887000	2.59631500
C	-5.97211200	-1.43825200	1.28547700
H	-4.54023100	-1.42995400	-0.31421400
C	-5.43341700	-0.08938000	3.20412800
H	-3.58362200	0.99496300	3.11567800
C	-6.30745800	-0.96692000	2.55472800
H	-6.63856400	-2.12104900	0.76468800
H	-5.67852800	0.28950700	4.19302400
H	-7.23294000	-1.27610700	3.03150200
O	-1.21767300	1.89317700	1.88606600
C	-1.47587500	-2.43374600	0.12563000
C	-1.78786100	-1.58268600	-0.94210300
C	-1.71709600	-2.09325000	-2.24744400
C	-1.33957100	-3.41569900	-2.48112500
C	-1.02889200	-4.25648800	-1.40642500
C	-1.09899400	-3.76049900	-0.10341500
C	-2.16109000	-0.11494200	-0.73438000
N	-0.96267000	0.74491400	-0.90598600
C	-1.11138400	1.94926000	-1.59062700
C	0.04727000	2.63338200	-2.03517000
C	-0.02920900	3.85631600	-2.69265700
C	-1.26704000	4.45221800	-2.95315000
C	-2.42187000	3.79455200	-2.52606900
C	-2.35587800	2.57574300	-1.85276300
H	-2.91485700	0.12369900	-1.49995600
H	1.01663400	2.16759700	-1.86712300
H	0.88789800	4.34087300	-3.02039100
H	-1.32868000	5.40220900	-3.47540600
H	-3.39704300	4.24125000	-2.70636600
H	-3.27661000	2.12620000	-1.49728800
H	-1.96007500	-1.44283600	-3.08431100
H	-1.30065300	-3.79522500	-3.49895000
H	-0.75095400	-5.29185700	-1.58492500
H	-0.87367400	-4.40915500	0.73899000
H	-1.54845700	-2.05411100	1.14013000
Cu	0.76966700	0.01147300	-0.49233900
C	3.19401900	2.92174700	2.17941400
C	4.39778000	2.24825100	2.24464200
C	4.53605500	1.00155300	1.59238600
C	3.40528000	0.51347500	0.88973500
C	2.12877200	2.35177700	1.45155900
C	5.74327800	0.22522600	1.61282700
C	3.47961900	-0.76555600	0.21721000
C	4.68530800	-1.50991300	0.26578500
C	5.81466700	-0.97759800	0.97588800
C	4.70704700	-2.75934300	-0.39248900
H	5.61455900	-3.35660900	-0.37769300
C	3.57265700	-3.20642700	-1.04080800
C	2.41913300	-2.40232500	-1.03765300
H	6.60283800	0.61558200	2.15051800
H	3.05591000	3.87673700	2.67506100
H	5.23963500	2.66098700	2.79411300
H	1.16366300	2.84537100	1.38206300
H	6.73123100	-1.56015700	0.99810200
H	3.55398500	-4.16382200	-1.54988500
H	1.50841800	-2.72905200	-1.52902400

N	2.23636200	1.18933400	0.82263200
N	2.37123100	-1.21461600	-0.43344900

T

C	-1.79656100	1.38650200	-0.33930900
C	-2.78541000	0.54617400	0.14732700
C	-3.88302400	0.74048300	1.06029800
C	-4.76849600	-0.30876100	1.39387900
C	-4.11302900	2.00501300	1.65580300
C	-5.82581300	-0.10471600	2.27868200
H	-4.62086700	-1.29145700	0.95323400
C	-5.17142900	2.19827500	2.53631400
H	-3.44687400	2.82496400	1.40442700
C	-6.03856400	1.14752300	2.85891100
H	-6.49034500	-0.93265700	2.51523200
H	-5.32515200	3.18102900	2.97650300
H	-6.86469600	1.30431500	3.54668700
O	-1.36763200	2.54566800	-0.27957900
C	-1.27796400	-1.78140500	1.41139400
C	-1.81998300	-1.88992200	0.12089200
C	-1.90412900	-3.16027000	-0.46430400
C	-1.43482900	-4.29272700	0.20656000
C	-0.87953900	-4.16935900	1.48211500
C	-0.80755600	-2.90965500	2.08456000
C	-2.29031800	-0.65508000	-0.62466800
N	-1.13284100	0.22471400	-1.14435800
C	-0.98841100	0.35586700	-2.57505100
C	-0.86762500	-0.79326800	-3.37340300
C	-0.70225700	-0.67845100	-4.75247500
C	-0.64121600	0.58071200	-5.35507500
C	-0.75477700	1.72190000	-4.56008800
C	-0.93030800	1.62052100	-3.17866700
H	-2.91378600	-0.96665200	-1.47424000
H	-0.90203800	-1.77350000	-2.90755100
H	-0.61923000	-1.57758800	-5.35736400
H	-0.50952600	0.66897400	-6.42950800
H	-0.71601700	2.70733200	-5.01673000
H	-1.02570600	2.50259700	-2.55679900
H	-2.34983900	-3.26275600	-1.45135500
H	-1.51403400	-5.27043600	-0.26141300
H	-0.52552400	-5.04991600	2.01130800
H	-0.40141600	-2.80904600	3.08757100
H	-1.26279900	-0.80507700	1.88847800
Cu	0.67384900	-0.05569200	-0.34934100
C	2.56059800	3.75582800	1.09375000
C	3.82887400	3.31478000	1.41460600
C	4.15427500	1.94722300	1.25519500
C	3.13489700	1.09881100	0.75969200
C	1.60885900	2.83523500	0.60482200
C	5.43972300	1.39160600	1.57155700
C	3.39937300	-0.31276900	0.58629300
C	4.67642300	-0.82991500	0.91970200
C	5.68999000	0.06089600	1.41152400
C	4.87810800	-2.21799700	0.74962100
H	5.84396500	-2.65266600	0.99236800
C	3.84391000	-3.00539200	0.28273700
C	2.60989400	-2.40279500	-0.02386700
H	6.21353100	2.05530500	1.94699300
H	2.28253800	4.79770300	1.21159900
H	4.58098900	4.00273000	1.79160000
H	0.59029500	3.11931300	0.33999100
H	6.66470400	-0.35058700	1.65742300
H	3.96545100	-4.07471900	0.14861000
H	1.77354700	-2.99406000	-0.38323300
N	1.89800900	1.54793100	0.44384400
N	2.39342500	-1.09730600	0.11447700

TS_{T-U}

C	1.20877500	-0.48282600	0.82667600
C	2.12957000	0.50180400	0.30260600
C	2.58280300	1.71575500	1.02852400
C	3.17776500	2.79124500	0.34361200
C	2.45626200	1.81014100	2.42868800
C	3.62971400	3.91813600	1.03104000
H	3.26785500	2.75722300	-0.73874900
C	2.91326400	2.93757500	3.11181700
H	1.99321800	0.99358200	2.97461200
C	3.50354600	3.99690600	2.41929000
H	4.07892500	4.73823500	0.47794700
H	2.80232900	2.98927500	4.19138000
H	3.85757600	4.87323400	2.95335900
O	0.97745400	-1.06811000	1.87154100
C	-0.12766300	2.47118400	-0.67233400
C	0.60485300	1.65716800	-1.55324200
C	0.59394700	1.96704800	-2.91980200
C	-0.14083100	3.05260400	-3.40372900
C	-0.87471600	3.84639100	-2.52079700
C	-0.86386500	3.55429100	-1.15405600
C	1.38316500	0.45956700	-1.05944400
N	0.49809800	-0.64938900	-0.47773500
C	0.41141800	-1.93944200	-1.13139300
C	0.38571900	-2.01703800	-2.53138700
C	0.28534400	-3.26033200	-3.15743700
C	0.20294500	-4.43090300	-2.40187100
C	0.21858500	-4.34817400	-1.00819100
C	0.31901000	-3.11287700	-0.36599800
H	1.97752200	0.05857100	-1.88597700
H	0.42742600	-1.11202700	-3.12812900
H	0.26827800	-3.30927900	-4.24216400
H	0.12212700	-5.39520400	-2.89312400
H	0.15131000	-5.25158300	-0.40909200
H	0.32767000	-3.04613200	0.71529900
H	1.17235500	1.35925200	-3.61225600
H	-0.13180900	3.28104500	-4.46541800
H	-1.43825400	4.69716000	-2.89189400
H	-1.41314500	4.18239800	-0.45903600
H	-0.08736200	2.28427200	0.39697600
Cu	-1.47186900	-0.17730400	-0.16453100
C	-3.15330900	-1.32002000	3.80472500
C	-4.50014100	-1.12028800	3.57625000
C	-4.93348800	-0.64928200	2.31519500
C	-3.93744200	-0.40400700	1.33824700
C	-2.23456400	-1.04834200	2.77225400
C	-6.31271300	-0.41397000	1.99362500
C	-4.32475700	0.07916100	0.03025600
C	-5.69541800	0.30649200	-0.25030800
C	-6.67796100	0.04523800	0.76368000
C	-6.02418300	0.78759700	-1.53790200

H	-7.06434800	0.97189700	-1.79107600
C	-5.02026000	1.02031800	-2.45695700
C	-3.68688700	0.76446900	-2.08994600
H	-7.06306500	-0.60865800	2.75401300
H	-2.79251300	-1.68057600	4.76173000
H	-5.23280000	-1.32042100	4.35295300
H	-1.16583400	-1.19328200	2.90551300
H	-7.72326900	0.22228700	0.52913400
H	-5.24105700	1.39282400	-3.45106500
H	-2.87381800	0.94396800	-2.78621900
N	-2.61660700	-0.60272200	1.57871800
N	-3.34664700	0.30326500	-0.88871600
N	4.44020500	-1.01722200	0.02942300
C	5.55778800	-0.37632500	0.79907800
H	5.61029600	0.65931000	0.45885800
C	4.02027900	-2.35266700	0.56960300
H	3.16698100	-2.67233600	-0.03638300
C	4.76659700	-1.11360500	-1.42859600
H	3.90126000	-1.58546600	-1.90541000
H	5.61711600	-1.79259500	-1.55676500
C	5.07506200	0.22159900	-2.10288900
H	6.01874500	0.65328700	-1.76203200
H	5.16458700	0.05655600	-3.18056000
H	4.28355100	0.95757300	-1.93953600
C	5.09095400	-3.44384900	0.58133200
H	4.66494700	-4.34426100	1.03353200
H	5.42748500	-3.71465900	-0.42263700
H	5.96642000	-3.16432000	1.17371700
H	3.63626500	-2.17767700	1.57604000
H	6.49773100	-0.86603400	0.51393200
C	5.39078300	-0.39527600	2.31564200
H	5.45807500	-1.40094800	2.73749000
H	6.19820200	0.19770300	2.75478400
H	4.44669400	0.05878600	2.62446000
H	3.36183000	-0.29661000	0.14386800

U

O	1.04331900	-0.04350900	-3.07104900
Cu	-0.45203300	0.30424700	-0.13270600
N	-1.77632400	0.10533100	1.43559900
C	-2.91965000	-0.52287800	1.04640800
C	-4.00084300	-0.74291500	1.93661200
C	-3.85691900	-0.27874800	3.26388800
C	-2.69056900	0.35488200	3.64364800
C	-1.66788300	0.52542400	2.69405300
C	-3.02333600	-0.97854000	-0.32373300
C	-4.20274500	-1.63784800	-0.75097500
C	-5.27649000	-1.84466900	0.17901400
C	-5.17985100	-1.41479800	1.46819500
C	-4.25774300	-2.06168200	-2.09898500
C	-3.18558100	-1.82176900	-2.93468000
C	-2.05478300	-1.15801500	-2.42262800
N	-1.97495100	-0.75174600	-1.15792400
C	1.62810700	0.26545000	-2.06294200
C	2.97204800	-0.05759200	-1.42322800
C	3.30003100	-1.48520500	-1.04627600
C	2.48104600	-2.55495100	-1.42840200
C	2.82148700	-3.86402300	-1.07758200
C	3.98118600	-4.11640700	-0.34526000
C	4.80756700	-3.05408300	0.02992700
C	4.47126300	-1.74799700	-0.32125600
H	-0.73318800	1.00561600	2.96464800
H	-2.55006100	0.72023100	4.65475200
H	-4.66611700	-0.42706500	3.97315500
H	-1.19334400	-0.94758900	-3.04980500
H	-3.19937300	-2.13312500	-3.97330700
H	-5.14523400	-2.57102400	-2.46346000
H	-6.17190400	-2.35167700	-0.16751100
H	-5.99677200	-1.57342500	2.16552600
H	5.12127200	-0.92792200	-0.02807200
H	5.71737800	-3.24286300	0.59154900
H	4.24640900	-5.13420600	-0.07618700
H	2.18257900	-4.68584100	-1.38701400
H	1.59218800	-2.37329600	-2.02446100
H	3.76399300	0.33762400	-2.07283500
C	1.93679300	-0.38499300	1.66809500
C	2.60019900	0.73130500	1.13373800
C	3.28664500	1.58774700	2.00581300
C	3.30365900	1.34606500	3.38080100
C	2.63071200	0.24050500	3.90253100
C	1.94890300	-0.62505600	3.04278800
C	2.60424500	1.03509900	-0.33914300
N	1.24391300	1.17270200	-1.01432300
C	0.59456000	2.45794100	-1.21544300
C	-0.11440200	2.72770800	-2.39436800
C	-0.72015900	3.97512100	-2.55108400
C	-0.62895500	4.94550400	-1.55168700
C	0.07764000	4.66495900	-0.38085700
C	0.68842200	3.42360700	-0.20386000
H	3.17202600	1.95176600	-0.52101100
H	-0.17826100	1.97947900	-3.17413300
H	-1.26075300	4.18746200	-3.46828800
H	-1.10178500	5.91307200	-1.68534800
H	0.15907700	5.41386700	0.40081500
H	1.23379900	3.20735100	0.70829800
H	3.81510800	2.44984000	1.60609200
H	3.84299200	2.01893900	4.04017300
H	2.64755300	0.04645600	4.97063500
H	1.44409200	-1.50086600	3.43950100
H	1.44463600	-1.09679100	1.01097700

TS$_{5\text{-}v}$

O	-0.50918300	-0.57817000	-0.56098600
Cu	1.40555500	0.74543100	0.23261800
N	3.03354000	0.08396600	-0.90784900
C	3.66245000	-0.98962400	-0.35901400
C	4.79953400	-1.59074100	-0.95623000
C	5.27889200	-1.02625400	-2.15966800
C	4.63419300	0.06674800	-2.70313300
C	3.50877400	0.59158700	-2.04270800
C	3.14191300	-1.52915400	0.87883100
C	3.76684100	-2.65989100	1.46363700
C	4.91277000	-3.24527300	0.82739300
C	5.40990600	-2.73033500	-0.33171800
C	3.21676600	-3.15604300	2.66716100
C	2.11635600	-2.53298100	3.22105900
C	1.57169600	-1.40978000	2.57079400
N	2.06473200	-0.92111900	1.43754500

C	-1.63104600	-0.57953100	-0.13507300
C	-2.56420900	-0.18577700	0.73167800
C	-4.03734600	-0.25947900	0.73717900
C	-4.80874300	0.18568200	-0.35000000
C	-6.20306900	0.14267300	-0.30410600
C	-6.85549100	-0.31840000	0.84087200
C	-6.10177000	-0.73008400	1.94262600
C	-4.70865600	-0.70067000	1.89102900
H	2.97486800	1.44469100	-2.44989300
H	4.97762000	0.52416900	-3.62437900
H	6.14962000	-1.45811700	-2.64477000
H	0.71598300	-0.88827500	2.98910100
H	1.67117600	-2.88911900	4.14358200
H	3.66633900	-4.02178700	3.14500800
H	5.37938300	-4.10888300	1.29172700
H	6.27903800	-3.17661200	-0.80552400
H	-4.12843900	-1.03282300	2.74782000
H	-6.59973600	-1.07957800	2.84251500
H	-7.94021700	-0.34394300	0.87977300
H	-6.78037300	0.48830900	-1.15697400
H	-4.30829800	0.58989800	-1.22409900
H	-2.09317400	0.33657100	1.56804800
N	-2.51338500	-2.33178400	-1.47733200
C	-1.25392400	-3.02656200	-1.83289200
H	-0.57154800	-2.26872700	-2.22432800
C	-3.44283100	-3.17602000	-0.68221300
H	-4.31372300	-2.55959900	-0.45700700
C	-3.17935600	-1.81643200	-2.69952300
H	-4.17794400	-1.48584900	-2.40397700
H	-3.31395500	-2.63441800	-3.42460500
C	-2.45246600	-0.66319500	-3.39065400
H	-1.46469200	-0.95335800	-3.75973600
H	-3.04051000	-0.33828200	-4.25461100
H	-2.32981500	0.19452400	-2.72335100
C	-3.90410700	-4.48870200	-1.33210800
H	-4.57334400	-5.01061300	-0.64102100
H	-4.46175100	-4.31841200	-2.25746800
H	-3.07291800	-5.16355600	-1.55717800
H	-2.95955600	-3.38332900	0.27571400
H	-1.44484400	-3.73783000	-2.65180100
C	-0.55744300	-3.75183500	-0.68161000
H	-1.12299600	-4.61480800	-0.32116100
H	0.40986200	-4.12226300	-1.03595700
H	-0.37054400	-3.08023300	0.15931800
C	0.19650900	2.37152400	-2.24807200
C	0.17396800	3.50928100	-1.41869800
C	0.19133500	4.78760400	-2.00776400
C	0.28055300	4.92966100	-3.39018600
C	0.32492900	3.79517800	-4.20388500
C	0.27273400	2.51973100	-3.63100300
C	0.11190100	3.43971400	0.03830400
N	0.49078800	2.44716400	0.77832800
C	0.26689400	2.56595300	2.18858000
C	1.31523400	2.27621400	3.07279800
C	1.11820800	2.39779400	4.44726100
C	-0.12727100	2.78375800	4.95142200
C	-1.17666500	3.05248800	4.07163400
C	-0.98506600	2.94770500	2.69267900
H	-0.26555700	4.33834500	0.53621200
H	2.28203900	1.98409400	2.67434400
H	1.94082600	2.19455700	5.12624600
H	-0.27858000	2.86956000	6.02279100
H	-2.15058100	3.34070900	4.45525900
H	-1.80859100	3.13108600	2.00915000
H	0.14962200	5.66969800	-1.37420900
H	0.30642400	5.92051000	-3.83230700
H	0.38059900	3.90363800	-5.28284300
H	0.26930400	1.63976100	-4.26730300
H	0.10423700	1.38137100	-1.81306100

V

O	-0.51543400	0.11308600	-1.22867500
Cu	0.99564300	0.31077800	0.24066100
N	2.94119200	0.23341600	-0.66729900
C	3.53037000	-0.98673500	-0.55954900
C	4.82311200	-1.25634100	-1.07952200
C	5.50261300	-0.19697900	-1.72127100
C	4.89457500	1.03844500	-1.82064200
C	3.60766000	1.20932300	-1.27839200
C	2.80085700	-2.04343400	0.10952100
C	3.38592500	-3.33190900	0.22106000
C	4.69469200	-3.56695400	-0.31999900
C	5.38479100	-2.57047300	-0.94151900
C	2.63353700	-4.33411800	0.87377400
C	1.38116100	-4.03021700	1.36984900
C	0.88859200	-2.72084500	1.21713400
N	1.57227900	-1.75564600	0.61062100
C	-1.71433100	-0.26407900	-1.04848100
C	-2.42416200	-0.37119600	0.10643200
C	-3.84571900	-0.66382600	0.44193900
C	-4.84713600	0.31716800	0.31910100
C	-6.16368800	0.06405600	0.71186600
C	-6.50686100	-1.17345800	1.25731000
C	-5.52194000	-2.15153500	1.41472500
C	-4.20965700	-1.89675400	1.01566200
H	3.10239600	2.16861800	-1.34397100
H	5.38869200	1.87317200	-2.30583200
H	6.49531700	-0.36578400	-2.12889400
H	-0.08963300	-2.44764000	1.60150900
H	0.77913900	-4.77689000	1.87632800
H	3.04898200	-5.33243300	0.97914500
H	5.12599600	-4.55882800	-0.22142700
H	6.37568700	-2.75508100	-1.34591300
H	-3.44835800	-2.66199200	1.14241400
H	-5.77611300	-3.11342400	1.85098700
H	-7.52860400	-1.37080900	1.56732400
H	-6.91681400	0.83954100	0.60445500
H	-4.58054900	1.29889900	-0.06282700
H	-1.80396700	-0.12462400	0.96774200
N	-2.41161900	-0.63461500	-2.46649000
C	-1.32850200	-0.76208800	-3.52679200
H	-0.73415100	0.14338200	-3.45374900
C	-3.19509100	-1.93329800	-2.35045400
H	-3.96732000	-1.75829300	-1.60617200
C	-3.35701400	0.49304000	-2.86039900
H	-4.19612500	0.43473400	-2.16744900
H	-3.72708900	0.26016100	-3.86187500
C	-2.74433900	1.88813700	-2.83875600

H	-1.94369300	2.00816900	-3.57214700
H	-3.53238400	2.60204900	-3.09687400
H	-2.35929700	2.14933400	-1.85158400
C	-3.82617100	-2.45423600	-3.64042700
H	-4.35493600	-3.37997300	-3.39400800
H	-4.56696500	-1.76780500	-4.05762100
H	-3.09645100	-2.69459500	-4.41704800
H	-2.50383700	-2.66427700	-1.93024600
H	-1.84840700	-0.77236300	-4.48760500
C	-0.41639000	-1.97553400	-3.38472600
H	-0.92906300	-2.92496200	-3.55732900
H	0.36986500	-1.89063700	-4.14144400
H	0.06221500	-1.98892200	-2.40482400
C	0.47841100	3.31939600	-0.80670100
C	0.58311900	3.78641000	0.51808700
C	0.85988300	5.14761000	0.74379600
C	1.07287200	6.01714200	-0.32322300
C	0.98969000	5.54151900	-1.63353300
C	0.68384500	4.19684100	-1.86959700
C	0.40744200	2.93402700	1.69388100
N	0.51235400	1.64615900	1.73523200
C	0.23610800	1.02254300	2.99387600
C	1.17353300	0.13477800	3.53978900
C	0.92184600	-0.47124400	4.76916700
C	-0.27253000	-0.21982800	5.45097300
C	-1.21358000	0.64937100	4.89842700
C	-0.96294100	1.27455000	3.67577000
H	0.20103200	3.46760800	2.62787800
H	2.09932500	-0.05217000	3.00577500
H	1.66239500	-1.13989300	5.19798600
H	-0.46804100	-0.70111300	6.40406700
H	-2.14856800	0.84199200	5.41564300
H	-1.70329600	1.93466900	3.23406600
H	0.92047200	5.51855800	1.76369900
H	1.29718200	7.06218900	-0.13415200
H	1.14763100	6.21816300	-2.46808400
H	0.59162600	3.83547300	-2.88992100
H	0.19859400	2.28771000	-1.00118300

TS$_{V\text{-}W}$

O	-1.19719900	1.63760100	-0.56093400
Cu	1.77762300	-0.62562500	-0.16648600
N	3.67823400	-1.14582200	0.49214400
C	4.62167500	-0.18400600	0.31482600
C	5.98245900	-0.37718000	0.66043900
C	6.34123300	-1.62723100	1.21374500
C	5.37289300	-2.59524000	1.39244100
C	4.04729800	-2.31257800	1.01506800
C	4.20419600	1.07848800	-0.25400100
C	5.16275400	2.10184900	-0.46344500
C	6.53333300	1.87092100	-0.10312400
C	6.92552600	0.68267700	0.43685500
C	4.70127000	3.31292800	-1.02745200
C	3.36390300	3.45153600	-1.34369300
C	2.48865200	2.37684800	-1.09412800
N	2.89357200	1.22721300	-0.56652900
C	-2.24203500	1.09710900	-0.32577100
C	-2.53481500	-0.33590800	-0.34126200
C	-3.79700100	-0.96290200	0.17589800
C	-4.11293700	-1.01061600	1.54596900
C	-5.25336800	-1.67825800	1.99848800
C	-6.09206400	-2.33398900	1.09629700
C	-5.77487500	-2.32680900	-0.26376900
C	-4.63862200	-1.65573900	-0.71548500
H	3.26447300	-3.05450800	1.13842800
H	5.61535200	-3.56485300	1.81301900
H	7.37468200	-1.81425800	1.49137300
H	1.42920000	2.44971400	-1.32484700
H	2.98201500	4.36844300	-1.77953700
H	5.40340300	4.12217000	-1.20734300
H	7.25512800	2.66490100	-0.27052200
H	7.96420200	0.51621800	0.70646600
H	-4.39666300	-1.66556200	-1.77541800
H	-6.40963100	-2.84776200	-0.97442500
H	-6.97470600	-2.85756600	1.45014900
H	-5.47629400	-1.69816900	3.06133600
H	-3.44660800	-0.55030200	2.26760900
H	-2.38005400	-0.63233000	-1.38760500
N	-3.49344700	2.25794200	-0.06553600
C	-2.91226700	3.60942600	-0.44104000
H	-1.96701000	3.68932600	0.09117900
C	-4.66705800	1.89557200	-0.95737400
H	-5.00790800	0.91547100	-0.63187700
C	-3.94077800	2.30361400	1.38516400
H	-4.46760700	1.36911700	1.57106800
H	-4.66575500	3.11813100	1.46124200
C	-2.82218900	2.50491900	2.39989000
H	-2.32945900	3.47466200	2.29745100
H	-3.26701000	2.47206300	3.39885700
H	-2.06341100	1.72107700	2.35088900
C	-5.83548700	2.88080400	-0.95173600
H	-6.57724700	2.51487100	-1.66770400
H	-6.33157000	2.93950900	0.01969600
H	-5.55364700	3.88945500	-1.26218500
H	-4.26388700	1.78258200	-1.96545400
H	-3.59787000	4.35647200	-0.03254000
C	-2.68235700	3.85486600	-1.92978100
H	-3.61017200	3.92137400	-2.50198700
H	-2.17295500	4.81800400	-2.03027200
H	-2.03952700	3.09471800	-2.37563400
C	0.06987400	0.17777500	1.98918600
C	-0.68506100	-0.96593600	1.67142500
C	-1.04051800	-1.83607200	2.71291400
C	-0.65871600	-1.57747700	4.03132800
C	0.08952300	-0.43892700	4.33404600
C	0.45178700	0.43717700	3.30806100
C	-1.06039900	-1.31994600	0.25587600
N	-0.01856200	-1.25592200	-0.64795800
C	-0.19829000	-1.81399000	-1.91207200
C	0.77931200	-1.55898700	-2.90626100
C	0.67295700	-2.08089600	-4.18770700
C	-0.42110800	-2.87933800	-4.54528500
C	-1.39637700	-3.14339900	-3.58728400
C	-1.29815400	-2.62280600	-2.29357500
H	-1.62438900	-2.25955200	0.26145200
H	1.62561600	-0.92605900	-2.64636600
H	1.44643700	-1.86105300	-4.91867000
H	-0.50367000	-3.28753000	-5.54744900

H	-2.24640500	-3.77273400	-3.83816200
H	-2.06022600	-2.89857200	-1.57269500
H	-1.61210600	-2.73162000	2.48548100
H	-0.93986500	-2.27068200	4.81871400
H	0.39028600	-0.23737500	5.35781300
H	1.03351200	1.32685200	3.53344800
H	0.34428000	0.87411100	1.20370000

W

O	2.48029800	-0.10820900	-2.15477000
Cu	-1.54456200	0.31292700	0.11895700
N	-3.12325700	-1.14379400	0.65063100
C	-4.34077000	-0.70092000	0.25552000
C	-5.53804600	-1.41521100	0.51151200
C	-5.42397500	-2.63732600	1.21273700
C	-4.17754600	-3.07956400	1.60850200
C	-3.04682200	-2.29676300	1.30314600
C	-4.40041200	0.55512900	-0.45870200
C	-5.65590000	1.05098500	-0.89431300
C	-6.84801900	0.30032800	-0.61650300
C	-6.79145900	-0.88220800	0.05806400
C	-5.66591800	2.28044500	-1.58963200
C	-4.47593700	2.94370400	-1.81585700
C	-3.28051900	2.37501300	-1.34311400
N	-3.23453800	1.21829600	-0.68496500
C	2.75313200	0.33691100	-1.08282000
C	2.67484000	-0.35129100	0.25110500
C	3.67559300	-1.50408700	0.32035800
C	3.80060300	-2.46969500	-0.69011500
C	4.70758400	-3.52160000	-0.54977900
C	5.49374300	-3.63080700	0.59895000
C	5.37157900	-2.67824300	1.61158700
C	4.47287900	-1.62036200	1.46984700
H	-2.05114300	-2.62224200	1.58928500
H	-4.05629200	-4.01349900	2.14641200
H	-6.31725500	-3.21545500	1.43176400
H	-2.33057200	2.87692000	-1.50038400
H	-4.44881700	3.88975600	-2.34525700
H	-6.60886000	2.69197900	-1.93820600
H	-7.80000600	0.69697600	-0.95648600
H	-7.69861200	-1.44255400	0.26422000
H	4.38510900	-0.88228000	2.26397800
H	5.97645800	-2.75427300	2.51016200
H	6.19580000	-4.45222000	0.70395000
H	4.79479700	-4.26047200	-1.34072800
H	3.18980200	-2.40176300	-1.58341400
H	2.94806100	0.35405400	1.03393500
N	3.38337000	1.82819300	-1.10630300
C	3.26818300	2.38546200	-2.51770500
H	3.66663000	1.62088900	-3.18069200
C	2.65524000	2.71550900	-0.09491800
H	2.83478000	2.28776300	0.88976200
C	4.85677900	1.69768600	-0.71612600
H	4.86548900	1.28121000	0.29255300
H	5.24401400	2.71636600	-0.66169300
C	5.71084200	0.84850500	-1.65116500
H	5.82293100	1.29355700	-2.64189700
H	6.70923700	0.78115300	-1.20968300
H	5.33327300	-0.17100100	-1.75577400
C	3.06383000	4.18504100	-0.08935400
H	2.45279200	4.67084500	0.67679800
H	4.10880900	4.33970300	0.19059100
H	2.87524300	4.69710000	-1.03554300
H	1.59180600	2.59095500	-0.29362000
H	3.93441400	3.25056200	-2.54987200
C	1.85799400	2.76379000	-2.95888300
H	1.44391300	3.60124700	-2.39406000
H	1.91308000	3.07036200	-4.00755000
H	1.17277700	1.91752800	-2.89782000
C	-0.04778500	-1.54242600	-1.49020000
C	0.55836500	-1.83226500	-0.25634300
C	0.58272700	-3.16820400	0.16944900
C	0.02141300	-4.18538200	-0.60612800
C	-0.57629400	-3.88210000	-1.82993100
C	-0.60731500	-2.55665800	-2.26953200
C	1.15457000	-0.75793700	0.63953300
N	0.32229900	0.42082100	0.73192900
C	0.51710200	1.25680000	1.82569200
C	-0.18673700	2.49377000	1.86711400
C	-0.02507300	3.40468500	2.90222700
C	0.85329400	3.14204700	3.96281400
C	1.54813600	1.93579400	3.95479300
C	1.39634400	1.01116000	2.91502700
H	1.31145700	-1.24034200	1.61715300
H	-0.87797000	2.71281400	1.05521600
H	-0.59398800	4.33127200	2.88633000
H	0.97703400	3.85250200	4.77339500
H	2.22122200	1.69409200	4.77403600
H	1.93628800	0.07284000	2.98906300
H	1.04824900	-3.41519900	1.12002600
H	0.05341500	-5.21207400	-0.25327500
H	-1.01159100	-4.66996000	-2.43749100
H	-1.06411600	-2.31092500	-3.22410200
H	-0.07031400	-0.52029100	-1.85283000

TS$_{W-U}$

O	-1.31400400	-0.22389000	1.60993600
Cu	1.17521900	0.13188700	-0.16762400
N	3.14312100	0.10218000	-1.00226800
C	4.09079000	-0.07112400	-0.04505200
C	5.47560600	-0.12608300	-0.34240200
C	5.85336600	0.00632800	-1.69789700
C	4.88063200	0.17945200	-2.66221400
C	3.53004300	0.22231600	-2.26841800
C	3.65210100	-0.20897000	1.32636500
C	4.61264200	-0.39662500	2.35181400
C	6.00747700	-0.44177400	2.01415000
C	6.42097200	-0.31219400	0.72221700
C	4.13142900	-0.53590800	3.67341100
C	2.77210500	-0.48883900	3.91294800
C	1.89346500	-0.29749400	2.83032700
N	2.31799000	-0.15859700	1.57697900
C	-1.91005700	0.25272100	0.67725400
C	-2.66762100	-0.44708200	-0.46327000
C	-3.49996800	-1.67741400	-0.17833500
C	-3.13045600	-2.66137300	0.75164400
C	-3.92160000	-3.79676600	0.93254500
C	-5.08887500	-3.97231800	0.18758100

C	-5.46868700	-2.99951000	-0.73841400
C	-4.68228100	-1.86119000	-0.91324800
H	2.74196100	0.35909400	-3.00286100
H	5.13802700	0.28289700	-3.71053500
H	6.90502500	-0.03032300	-1.96737600
H	0.81779600	-0.25929000	2.97405200
H	2.37347500	-0.59691500	4.91573800
H	4.83606600	-0.68139600	4.48727600
H	6.72968300	-0.58411700	2.81244000
H	7.47787300	-0.34955400	0.47573300
H	-4.98661400	-1.10806300	-1.63716700
H	-6.37644400	-3.12361600	-1.32119700
H	-5.69977700	-4.85848800	0.33019000
H	-3.62311300	-4.54707300	1.65875600
H	-2.23250400	-2.53391800	1.34375700
H	-3.32049000	0.29963200	-0.92303100
N	-2.92074900	1.72482600	1.34458800
C	-2.17811900	2.21874900	2.54568300
H	-1.95219900	1.33608300	3.14266100
C	-3.12767800	2.79289000	0.31210900
H	-3.65161700	2.32522100	-0.52472600
C	-4.25646100	1.15182200	1.75456500
H	-4.68539000	0.69371700	0.86006800
H	-4.90255800	1.99234800	2.02528800
C	-4.25060100	0.14568100	2.90356700
H	-3.98413000	0.60645800	3.85770100
H	-5.26707100	-0.24562600	3.00471600
H	-3.58540800	-0.69810700	2.72059700
C	-3.89393500	4.04229600	0.75890100
H	-3.91671900	4.73768600	-0.08527300
H	-4.93058000	3.84064700	1.03862200
H	-3.40565300	4.55717700	1.59050200
H	-2.14540300	3.07973200	-0.05262900
H	-2.86160100	2.84808600	3.12675100
C	-0.88092900	2.96195400	2.24859700
H	-1.03499600	3.90577300	1.72081300
H	-0.39586100	3.19541000	3.20135500
H	-0.19716300	2.34322900	1.66475200
C	0.03595100	-2.37156200	-0.41042500
C	-0.61977500	-1.78466000	-1.50780500
C	-0.50353700	-2.40605400	-2.75884700
C	0.24799600	-3.57160800	-2.92062400
C	0.90037500	-4.13960100	-1.82581400
C	0.78943900	-3.53683300	-0.57127000
C	-1.42462400	-0.50025300	-1.41329000
N	-0.67450700	0.65297400	-0.81162000
C	-0.64973400	1.86283100	-1.56877200
C	0.30062500	2.84449700	-1.21450600
C	0.36656300	4.06971100	-1.87309900
C	-0.50561200	4.35541100	-2.92640400
C	-1.44420300	3.39375500	-3.30003100
C	-1.52377500	2.17010700	-2.63260000
H	-1.75109400	-0.27978300	-2.42977000
H	0.99783000	2.62781400	-0.40862500
H	1.11292500	4.79843900	-1.56967700
H	-0.44858100	5.30415600	-3.44998800
H	-2.12905300	3.59300100	-4.11952700
H	-2.28637300	1.46682100	-2.94873800
H	-1.00708800	-1.97289100	-3.61965100
H	0.32169700	-4.03358000	-3.90064600
H	1.48298000	-5.04787100	-1.94681400
H	1.27961400	-3.98093100	0.29053000
H	-0.06607900	-1.93689800	0.57812500

TS_{K-S}

C	1.25362400	-0.16980800	-1.48353900
C	1.53494900	0.33512800	-0.28431900
C	1.76003600	1.78876300	-0.08269700
C	1.76390300	2.33413100	1.21647600
C	2.00032200	2.66690100	-1.15807200
C	1.99457400	3.69455600	1.42841200
H	1.59035500	1.68475300	2.06869000
C	2.21029700	4.02786900	-0.94638700
H	2.03001200	2.27227300	-2.17025200
C	2.21233100	4.55214200	0.34874300
H	2.00287600	4.08319000	2.44344300
H	2.38893500	4.67900300	-1.79782100
H	2.38975900	5.61076900	0.51362500
Cu	-0.45913300	-0.17228400	0.41584400
C	-3.04885800	3.46118000	0.92916600
C	-4.22029100	3.05441600	0.32122800
C	-4.29281300	1.76770200	-0.25615700
C	-3.13774000	0.94742800	-0.17183500
C	-1.95674000	2.57505800	0.96822900
C	-5.46745800	1.26966100	-0.91444500
C	-3.15905800	-0.37257000	-0.76290300
C	-4.33649800	-0.83093200	-1.40702900
C	-5.48874200	0.02330400	-1.46451400
C	-4.30576900	-2.12685700	-1.96904900
H	-5.18814600	-2.51345600	-2.47173300
C	-3.15492500	-2.88303100	-1.87099200
C	-2.03423100	-2.34195900	-1.21226300
H	-6.34289600	1.91086000	-0.96551300
H	-2.95686200	4.44573700	1.37504900
H	-5.08494100	3.71099100	0.27680300
H	-1.01812600	2.86847900	1.42942900
H	-6.38213400	-0.34531500	-1.96071300
H	-3.09869100	-3.88147300	-2.29101600
H	-1.11708900	-2.91199600	-1.10465400
N	-1.99644000	1.35419300	0.44286800
N	-2.03371200	-1.12536700	-0.67825200
O	1.06764600	-0.75341500	-2.48579600
C	4.30093000	-1.76809000	-1.00919100
C	3.93745900	-0.77607000	-0.08742100
C	4.83855000	0.26501700	0.17466400
C	6.07312300	0.32022600	-0.47442600
C	6.42314700	-0.66679900	-1.39727500
C	5.53267900	-1.71169200	-1.65957500
C	2.59632400	-0.82702100	0.60622700
N	2.04415300	-2.08633700	0.66137300
C	0.92984500	-2.26432600	1.39148700
C	0.29425800	-3.55233600	1.34334900
C	-0.86734700	-3.82495900	2.03405800
C	-1.50221500	-2.84123700	2.82910500
C	-0.91736100	-1.59048600	2.92117000
C	0.25961700	-1.26587900	2.19835200
H	2.64197600	-0.24586800	1.54271800
H	0.79028700	-4.31744600	0.75225600

H	-1.29911100	-4.82215500	1.97498500
H	-2.41053700	-3.07458600	3.37613400
H	-1.35346000	-0.83222400	3.56882100
H	0.80519800	-0.37513800	2.50158500
H	4.57293100	1.03472100	0.89471500
H	6.76245900	1.13133900	-0.25517900
H	7.38475500	-0.62710000	-1.90199800
H	5.80355900	-2.48995900	-2.36850300
H	3.60906300	-2.58585800	-1.17736400

TS$_{R-T}$

C	0.32947200	0.46593000	0.76823600
C	0.35527500	1.74537000	0.31354200
C	1.03276700	2.87147100	0.95161500
C	1.08579900	4.12599400	0.30952300
C	1.66828300	2.74416600	2.20617400
C	1.75419000	5.20243100	0.89433400
H	0.58638700	4.26272600	-0.64512400
C	2.33102300	3.82306500	2.78406600
H	1.63306300	1.79116500	2.72672200
C	2.38330400	5.06017200	2.13256700
H	1.77390500	6.15985100	0.38090800
H	2.80464700	3.70043800	3.75447400
H	2.89365900	5.90208200	2.59035000
Cu	2.73718000	-0.70405200	-1.40949000
C	5.13582600	2.53038000	0.48035700
C	6.11915600	1.79229300	1.10529500
C	6.10529400	0.38185200	1.00233900
C	5.06336900	-0.20551500	0.24329000
C	4.14465700	1.85779800	-0.25938300
C	7.08419500	-0.45952100	1.63076700
C	4.99703100	-1.64532900	0.12439400
C	5.97758700	-2.44627800	0.76212500
C	7.02419600	-1.81596600	1.51528100
C	5.86478900	-3.84772600	0.61871300
H	6.59766100	-4.49456400	1.09256100
C	4.82577400	-4.37779600	-0.12038800
C	3.90078300	-3.50306300	-0.71859400
H	7.87746500	0.00986100	2.20492800
H	5.09970100	3.61085700	0.55645500
H	6.90019900	2.27877400	1.68263800
H	3.35352600	2.41537100	-0.74818000
H	7.76851800	-2.44419600	1.99523100
H	4.71261900	-5.44874700	-0.24721600
H	3.07295500	-3.88253000	-1.31044700
N	4.10900600	0.53456400	-0.38146800
N	3.98124800	-2.18124200	-0.60123000
O	0.56629100	-0.46626700	1.47371600
C	-2.42584900	2.86335800	0.10295200
C	-1.71627800	2.49982100	-1.04997500
C	-2.22795400	2.87271500	-2.30174800
C	-3.42687500	3.57980600	-2.40202000
C	-4.13081800	3.93046100	-1.24624400
C	-3.62590600	3.57292000	0.00516300
C	-0.42704000	1.68989000	-0.97372900
N	-0.72260200	0.21732000	-0.95247800
C	-0.01037000	-0.64520000	-1.75994900
C	-0.31915300	-2.03135500	-1.69616700
C	0.30652100	-2.96851200	-2.50808000
C	1.31303500	-2.59363100	-3.40852300
C	1.68377000	-1.24787100	-3.46604300
C	1.05916300	-0.26922100	-2.64360600
H	0.17214800	1.97179500	-1.84836500
H	-1.10692200	-2.34287900	-1.01548300
H	-0.00357200	-4.00819800	-2.44659800
H	1.78098800	-3.32217800	-4.06189700
H	2.41220200	-0.91673100	-4.20207500
H	1.23989500	0.77678000	-2.86781700
H	-1.67792600	2.61280000	-3.20363000
H	-3.80282500	3.87166700	-3.37867000
H	-5.05609900	4.49419300	-1.32121600
H	-4.15612700	3.86102600	0.90841800
H	-2.01973400	2.61156000	1.07798500
Cu	-2.54662500	-0.25377100	-0.33765600
C	-2.98023100	-2.38738200	3.63386500
C	-4.33507000	-2.65283900	3.63441600
C	-5.12919300	-2.23091900	2.54335300
C	-4.47119600	-1.54874300	1.48971300
C	-2.41602900	-1.70135800	2.53981500
C	-6.54471500	-2.45720000	2.46433500
C	-5.23704500	-1.08781700	0.35215200
C	-6.63455600	-1.32051000	0.31112000
C	-7.26653700	-2.01978800	1.39438500
C	-7.34094200	-0.83919000	-0.81428200
H	-8.41321800	-0.99937500	-0.88240100
C	-6.66017000	-0.16822900	-1.81051500
C	-5.27103400	0.01422200	-1.68456300
H	-7.03227500	-2.98348300	3.27963300
H	-2.34640700	-2.69516700	4.45842400
H	-4.80036900	-3.17916100	4.46313000
H	-1.35445500	-1.47014700	2.50392800
H	-8.33751600	-2.19208400	1.34409500
H	-7.17446800	0.21745300	-2.68377400
H	-4.70728900	0.54487400	-2.44490500
N	-3.13881300	-1.29647900	1.50017500
N	-4.57691600	-0.43498800	-0.64143600

TS$_{T-U'}$

C	1.21199200	-1.13163900	0.77966900
C	2.35284300	-0.48179500	0.15695300
C	3.73113700	-1.03271000	0.31693400
C	4.70759000	-0.79101400	-0.66706200
C	4.10166000	-1.78809500	1.44609200
C	6.00747300	-1.27956700	-0.52509500
H	4.44660300	-0.21899100	-1.55420200
C	5.40252800	-2.27187100	1.58431800
H	3.35860300	-2.00665400	2.20738400
C	6.36489300	-2.01859000	0.60374200
H	6.74119700	-1.08332200	-1.30203800
H	5.66250500	-2.85966400	2.46040000
H	7.37572400	-2.39849600	0.71423200
O	0.86964000	-1.51738000	1.88298900
C	0.64053800	1.65160100	-1.79955700
C	1.40987700	0.53248900	-2.15380900
C	1.98965600	0.50457700	-3.43002300
C	1.81470200	1.56363800	-4.32456600
C	1.04290800	2.66723200	-3.96061400
C	0.45262700	2.70701700	-2.69396600

C	1.63276300	-0.62887600	-1.21679300
N	0.39620800	-1.14874300	-0.47169200
C	-0.18222800	-2.40141600	-0.94988800
C	-0.47380100	-2.52245100	-2.31780700
C	-1.03880300	-3.69909400	-2.80679900
C	-1.32393200	-4.75913700	-1.94315800
C	-1.03199200	-4.63290700	-0.58490900
C	-0.46256400	-3.46230400	-0.07887000
H	2.05923600	-1.46167700	-1.78594900
H	-0.24849300	-1.70280100	-2.99302300
H	-1.25281500	-3.78606000	-3.86782600
H	-1.76274700	-5.67461900	-2.32669000
H	-1.23828200	-5.45494200	0.09405800
H	-0.22715200	-3.37353300	0.97397200
H	2.58168300	-0.35741700	-3.72751100
H	2.27566500	1.52065200	-5.30673000
H	0.90182400	3.48946300	-4.65550200
H	-0.14648600	3.56474900	-2.39998200
H	0.18441800	1.69928100	-0.81383600
Cu	-1.39195200	-0.20197500	-0.25332600
C	-3.32908500	-1.11651800	3.67907600
C	-4.59268400	-0.60481200	3.45953100
C	-4.88851000	0.02516100	2.22828300
C	-3.84721900	0.09393200	1.27012200
C	-2.35659100	-1.00009700	2.66652800
C	-6.17402100	0.58330600	1.91699800
C	-4.09443800	0.72528100	-0.00841000
C	-5.37560600	1.26689600	-0.28116600
C	-6.40729400	1.17901100	0.71374600
C	-5.57083500	1.87395400	-1.54240800
H	-6.54039100	2.29749600	-1.78834200
C	-4.52788500	1.92188900	-2.44614600
C	-3.28965900	1.35945600	-2.08892100
H	-6.96139300	0.52017700	2.66211100
H	-3.07494100	-1.60583600	4.61296600
H	-5.36433400	-0.68056300	4.22040000
H	-1.34781600	-1.38461100	2.79395100
H	-7.38273800	1.59775700	0.48559800
H	-4.64671700	2.38101000	-3.42123600
H	-2.44729800	1.38774200	-2.77321500
N	-2.61045500	-0.41310000	1.50070300
N	-3.07592700	0.77623800	-0.91083200
N	2.83688100	1.89286800	1.54219200
C	1.87041700	1.86828800	2.69104800
H	2.18759100	2.61174500	3.42939000
C	4.23694800	1.65117600	2.04704200
H	4.85494600	1.43638300	1.17523100
C	2.72207000	3.13832500	0.72063900
H	2.81999400	4.00695500	1.38272800
H	1.71127300	3.15160600	0.31211600
C	3.72145400	3.21699600	-0.43029500
H	3.66932700	2.32970100	-1.06603900
H	3.47255000	4.08298400	-1.04980100
H	4.75156300	3.34182600	-0.08884500
C	4.85510600	2.77456000	2.87994200
H	5.85864100	2.46070700	3.18163400
H	4.96225700	3.70681000	2.31863400
H	4.29593700	2.98626800	3.79520600
H	4.19420100	0.72611200	2.62466800
H	1.97186300	0.87785100	3.14308300
C	0.40913700	2.10901500	2.32331400
H	0.05214400	1.39917700	1.57235800
H	-0.19734700	1.96044100	3.22149200
H	0.22197900	3.12612000	1.96956600
H	2.54274900	0.88622300	0.82724600
